# Free Choice in Quantum Theory: A *p*-adic View

**DOI:** 10.3390/e25050830

**Published:** 2023-05-22

**Authors:** Vladimir Anashin

**Affiliations:** 1 Faculty of Computational Mathematics and Cybernetics, Lomonosov Moscow State University, Leninskie Gory 1, 119991 Moscow, Russia; vladimir.anashin@cs.msu.ru or anashin@iisi.msu.ru; 2 Federal Research Center ‘Information and Control’ Russian Academy of Sciences, 119333 Moscow, Russia

**Keywords:** automaton, letter-to-letter transducer, sequential Mealy machine, *p*-adic 1-Lipschitz map, interpretation of quantum mechanics, Planck’s scale, experimenter’s free choice

## Abstract

In this paper, it is rigorously proven that since observational data (i.e., numerical values of physical quantities) are rational numbers only due to inevitably nonzero measurements errors, the conclusion about whether Nature at the smallest scales is discrete or continuous, random and chaotic, or strictly deterministic, solely depends on experimentalist’s free choice of the metrics (real or *p*-adic) he chooses to process the observational data. The main mathematical tools are *p*-adic 1-Lipschitz maps (which therefore are continuous with respect to the *p*-adic metric). The maps are exactly the ones defined by sequential Mealy machines (rather than by cellular automata) and therefore are causal functions over discrete time. A wide class of the maps can naturally be expanded to continuous real functions, so the maps may serve as mathematical models of open physical systems both over discrete and over continuous time. For these models, wave functions are constructed, entropic uncertainty relation is proven, and no hidden parameters are assumed. The paper is motivated by the ideas of I. Volovich on *p*-adic mathematical physics, by G. ‘t Hooft’s cellular automaton interpretation of quantum mechanics, and to some extent, by recent papers on superdeterminism by J. Hance, S. Hossenfelder, and T. Palmer.

## 1. Introduction

The main goal of the current paper is to prove some of results which were announced without proofs in [1], namely, to prove rigorously mathematical statements which show that an experimentalist’s conclusions about whether Nature on the smallest of scales is discrete or continuous [2], random and chaotic, or strictly deterministic [3] solely depends on the experimentalist’s free choice of the metrics he chooses to process the measurement data which basically are rational numbers due to inevitably nonzero measurement errors. It should be stressed that the said statements are not types of free-will theorems in quantum mechanics since the statements are about how the data obtained during experiments are postprocessed rather than about how an experimentalist chooses the measurement setting during experiments. This is a crucial difference between results of the current paper and, for example, a Conway–Kochen strong free will theorem [4]. *In order to distinguish between these two faces of experimentalist’s freedom, in this paper, the two terms "free choice" and “free will” are used, and they are **not interchangeable***.

There is some resemblance between the meanings of terms used in the invariant set theory [3,5] (within which a *p*-adic metric is briefly mentioned) and in the current paper; however, the current paper discusses a mathematical model for postprocessing of measurement data rather than broader physical theories.

The paper is inspired by the ideas of I. Volovich who, in collaboration with V. Vladimirov in the 1980s laid the cornerstone of contemporary *p-adic mathematical physics* [6]. The paper is motivated also by the ideas of G. ‘t Hooft who initiated the development of the *cellular automaton interpretation of quantum mechanics* [7] which is based on a suggestion that on some basic level there is no intrinsic randomness in nature.

More formally, *the paper introduces a wide class of functions, each of which can be regarded as a continuous (and sometimes as a chaotic, having positive entropy) real function over continuous real time with respect to real metric and which simultaneously is strictly deterministic (and a nonchaotic, having zero entropy) causal function over discrete time with respect to the p-adic metric for every p>1*. By the common definition, *causal functions* are the mappings which can be performed by automata but only those automata which are the so-called letter-to-letter transducers (or, sequential Mealy machines whose sets of states are not necessarily finite) over a *p*-letter alphabet rather than by cellular automata on which G. ‘t Hooft’s interpretation is based. These classes of automata differs both from algorithmic and physical points of view. From the algorithmic point of view, letter-to-letter transducers can be judged as the least powerful computers compared to cellular automata which are the most powerful ones. Any algorithm (i.e., any general recursive function) can be implemented on a suitable cellular automaton since the class of all cellular automata is Turing-complete [8,9], whereas algorithms which can be implemented by the transducers are necessarily primitive recursive functions, and moreover, constitute a small class of primitive recursive functions; see the end of Section 3.3. From a physical point of view, the sequential machines are models of *open systems* whereas cellular automata are models of *isolated systems*. In contrast to a sequential machine, a cellular automaton updates its states according only to a fixed local rule which does not depend on input, whereas the next state of a sequential machine depends both on input information and on a current state; the sequential machine produces output information which also depends both on input information and on the current state. Throughout this paper, the term ***automaton** refers to a sequential Mealy machine with a potentially infinite number of states*; for a formal definition of the latter machine see Definition 2. In what follows, types of automata different from the said Mealy machines are mentioned with respective adjectives, e.g., “cellular automaton” and “push-down automaton”.

The paper is organised as follows:

- In Section 2, we recall a formal definition of causal function over discrete time (cf., Definition 1). The very term “causality” is based on the notion of time; this is why in the paper, “time” as a measurable physical entity is a central theme: time may be either discrete (e.g., Planck time) or continuous (e.g., real time) at respective “ends of scale”. In this paper, we generally advocate that these cases are indistinguishable by measurements and actually are subject to an experimentalist’s free choice of metric with respect to which he processes the numerical values of the experimental physical data. After the formal definition of causality over discrete time, we introduce as postulates statements of I. Volovich on indistinguishability using measurements of physical quantities between rational and irrational values and of G. ‘t Hooft on the nonexistence of randomness in Nature; then, we formalise the notion a “physical law” as a function which is consistent with these postulates, cf., Conditions 1.
- In Section 3, we review some notions and facts from *p*-adic analysis and from automata theory which will be needed further in the paper.
- In Section 4, we introduce one of the main notions of the paper; that is, the real causal functions which are the functions that are continuous both with respect to a real metric and to the *p*-adic metric; i.e., causal functions which reside simultaneously in two worlds, Archimedean and non-Archimedean. The main results described in these sections are as follows:
  – Theorem 5 completely describes the class of functions that satisfy Conditions 1; i.e., those which are completely consistent both with Volovich postulates and with ‘t Hooft causality postulate. We interpret this theorem to be a manifestation of the observer’s freedom to conclude whether Nature on the smallest of scales is discrete or continuous since the conclusion depends solely on the observer’s free choice of metric with respect to which the observer processes the measured numerical data.
  – In Section 4.4, we argue that the observer’s conclusion as whether Nature is basically random and chaotic or totally predictable and deterministic also depends solely on the observer’s free choice of metric with respect to which the observer processes the measured numerical data; namely, we show that maps which are chaotic with respect to the real metric are strictly deterministic and predictable with respect to *p*-adic metric, irrespective to which common definition of chaos is used.
  – In Section 4.5, we argue that Conditions 1 may be too restrictive from the physical point of view and relax the conditions, letting them hold only for some prime *p* rather than for all primes. This way we introduce a notion of a *p*-consistent function, show that the class of *p*-consistent functions is much wider than the class of completely consistent ones (Theorem 7), prove hologram-like property (Theorem 8) which shows that global behaviour of *p*-consistent functions is completely defined by their local behaviour, and then prove that wide classes of physically important functions (such as continuous real functions, real functions that vanish at infinity, *n*-th power integrable functions, wave functions) can be uniformly approximated by infinitely differentiable *p*-consistent functions; see Theorem 9. This theorem is yet one more piece of evidence supporting the notion that observer’s conclusion on discreteness, continuity, and reversibility of time solely depends on the observer’s free choice of metric. Finally, in this subsection, we prove Theorem 10 which yields that smooth *p*-consistent functions related to systems having a *finite number of states* are necessarily affine; this theorem may demonstrate where the *linearity* of operators used in quantum theory is rooted.
- In Section 5, we argue that “continuous” and “discrete” models of physical world “meet each other in the middle of the scales”, and the wave function is the “meeting point”. The specifics of this section are as follows:
  – In Section 5.1, we formalise what is meant by “measurements at each end of the scale” by introducing two observers, *Big-endian* and *Little-endian*, that perform measurements at respective ends, macro and micro.
  – In Section 5.2, we introduce a *p*-adic model of the instrument which measure and indicates time, a *p-adic clock*, and a respective notion of *p*-adic time, which is time by the Little-endian’s clock. Then, we outline (Theorem 11), which proves that there exists a unique clock which is the same for Little-endian and for Big-endian, the *universal clock*. We argue that the known effect in quantum theory of indistinguishability of which of two event happens earlier than does another one may be rooted in the fact that *p*-adic time cannot be ordered, i.e., that in contrast to the ring of integers Z, the ring $Z_{p}$ of *p*-adic integers cannot be ordered. Therefore, the existence or nonexistence of the “time arrow” is again subject to free choice of the metric by the experimentalist.
  – Section 5.3 describes the base on which the construction of wave function is founded. The section describes, in formal terms, the process of finding cluster points for experimental points in Euclidean space and constructing a smooth line (or surface) on which these cluster points fall. In the subsection, we mostly refer to results which were published earlier in [10,11] and interpret these as the models of physical systems having either discrete or continuous spectra. Based on these results, we argue that chaos is either immanent to continuous time models or emerges as a result of sufficiently long evolution of a physical system in discrete time models.
  – In Section 5.4, we construct two types of wave functions, the sharp one for Little-endian, with respect to discrete time, and the fuzzy one for Big-endian, with respect to continuous time. The fuzzy wave function can be approximated by sharp wave functions with any desirable accuracy, so this is again a subject to the free choice of the experimentalist regarding the type of wave function which depends on the experimentalist’s free choice of metric. We show then in (Theorem 18), that under a reasonable finiteness assumption, the fuzzy 1-dimensional wave function is actually a sharp *N*-dimensional wave function over discrete 2-adic time. Here, as an extra mathematical tool, we use β-expansions of numbers; the β-expansions were originally introduced in [12,13].
  – In Section 5.5, we formally derive a time-energy uncertainty relation in *entropic* form. Here, we use yet one more extra mathematical tool, the theory of prefix codes. All necessary notions, results, and proper references of this theory are given in the subsection. We stress that *no hidden variables are assumed*, and the uncertainty relation holds both for the Big-endian and for the Little-endian.
- We conclude in Section 6. Here, we state that basically the results of the paper may be treated as information–theoretic and remark that the paper highlights J. Wheeler’s “it from bit” doctrine [14] since the final results on wave functions, especially Theorem 18, show that “it” is “from bit” indeed: both sharp and fuzzy wave functions actually turn out to be 2-adic 1-Lipschitz functions, i.e., automata functions over the alphabet {0,1}.

## 2. Formalisation

I. V. Volovich, in his numerous papers, books, talks, etc., has stated, many times, the following postulates (further referred to as *Volovich postulates*) on which *p*-adic mathematical physics is founded:

(i) Only rational numbers can be observed; irrational numbers cannot.
(ii) Distances smaller than Planck length cannot be measured.
(iii) Fundamental physical laws should be invariant with respect to a change of number field.

According to Ostrowski’s theorem, every nontrivial absolute value on the rational numbers Q is equivalent to either the usual real absolute value or a *p*-adic absolute value (c.f., e.g., [15] [Theorem 10.1]). Then, to ensure the limits of convergent sequences over a field belong to the field; *the mentioned number fields must be the fields $Q_{p}$ of p-adic numbers or the field R of real numbers* since these fields are the only completions of the field Q with respect to absolute values on Q. Of course, the fields can be complete extensions of the fields $Q_{p}$ and R like, e.g., the fields of complex *p*-adic numbers $C_{p}$ or a field of “ordinary” complex numbers C, but $Q_{p}$ and R are the only “smallest“ fields which satisfy the third Volovich postulate.

G. ‘t Hooft in his book *The Cellular Automaton Interpretation of Quantum Mechanics* [7] makes the following claim (further referred to as the *‘t Hooft causality postulate*) which is fundamental for the cellular automaton interpretation of quantum mechanics:

It may well be that, at its most basic level, there is no randomness in Nature, no fundamentally statistical aspect to the laws of evolution. Everything, up to the most minute detail, is controlled by invariable laws. Every significant event in our universe takes place for a reason, it was caused by the action of physical law, not just by chance. This is the general picture conveyed by this book.

To be consistent with this postulate, a physical system must be *causal*; that is, the “effect”, which is the reaction of the system to a “cause”, i.e., to an impact the system has been exposed, must be a function of the “cause” and of the ”state“ of the system. However, the very notion of causality is based on the notion of “time” which must be a totally ordered set since the “effect” cannot happen earlier than can the “cause” whose function the “effect” is. It is impossible to experimentally distinguish rational numbers from real numbers (cf. Volovich first postulate); therefore it is reasonable to assume that “time” is a totally ordered *countable* set. It is well known that any totally ordered countable set *T* is order-isomorphic to a subset of Q (c.f., e.g., [16]) with respect to the natural order ≤ on Q. Time *T* is called *continuous* if the ordering of elements in *T* is dense; i.e., given $t_{1},t_{2}\in T$ there exists $t_{3}\in T$ such that $t_{1}<t_{3}<t_{2}$. Time *T* is called *discrete*, and if given any $t_{1},t_{2}\in T$, there is not more than a finite $t_{3}\in T$ such that $t_{1}<t_{3}<t_{2}$.

“Continuous” physical models are based on the assumption that any temporal/spatial interval can be divided into smaller intervals ad infinitum. The “discrete” models assume that spacetime should somehow be “quantized” at the smallest of scales; i.e., there exist the smallest spatial/temporal intervals which can not be divided into smaller ones, [2]. In the latter case, it would be reasonable to try to construct a mathematical theory assuming that total amount of these “indivisible” values can be increased ad infinitum. In the both cases, as well as in respective physical theories, the ”infinity“ simply stands for a value which is extremely small (or extremely large) compared to a given value so that calculations involving the notion of infinity result in values which agree with respective measured values up to a small real number, the error. Therefore, if theories of either type adequately describe physical reality at respective “ends of scale”, the theories must “meet one another somewhere in the middle of the scale”.

The discreteness implies that the indivisible intervals are respective units:, i.e., take values of 1; moreover, both “cause” and “effect” are sequences of “elementary causes” and “elementary effects” which happen at discrete time instants 0,1,2,….

Actually, the "time unit" is the longest temporal interval within which it is impossible for an observer to determine whether any two events are simultaneous or not; i.e., which of the two events happens earlier/later than another one does. In other words, an “event” is like a film consisting of frames where each frame is a static picture, but the sequence of the pictures produces a movie on a screen which the audience of the cinema sees as dynamical process. Thus, the “elementary event” (“elementary cause”, “elementary effect”) is an event that lasts exactly one time unit similar to a momentary splash for which the moment when it begins is undistinguishable from the moment when it finishes.

We recall a notion of causal function over discrete time in terms of general system theory, c.f., e.g., [17,18].

**Definition** **1** (Causality over discrete time)**.** Causal functions over discrete time *$N_{0}=\left\{0,1,2,\ldots \right\}$ are exactly the functions f which satisfy the following conditions:*

(i) *The domain (the “causes”) and range (the “effects”) of f are, accordingly, all sequences $a={\left(a_{i}\right)}_{i\in N_{0}}$ and $b={\left(b_{i}\right)}_{i\in N_{0}}$ over respective sets 𝒜, the “elementary causes”, and ℬ, the “elementary effects”;*
(ii) *If $f\left(a\right)={\left(b_{i}\right)}_{i\in N_{0}}$, then $b_{i}$ does not depend on $a_{i+1},a_{i+2},\ldots$, for all $i\in N_{0}$.*

*In other words, the function f is causal if and only if there exists a sequence ${\left(\varphi_{i}^{f}\right)}_{i=0}^{\infty }$ of maps $\varphi_{i}^{f}:{𝒜}^{i+1}\to ℬ$, $\left(i\in N_{0}\right)$, such that*

(1) $$f\left(a\right)={\left(\varphi_{i}^{f}\left(a_{0},\ldots ,a_{i}\right)\right)}_{i\in N_{0}}$$

It is reasonable to assume that both sets 𝒜 of “elementary causes” and ℬ of “elementary effects” contain at least two elements and, moreover, that the sets are finite since no physical objects are known which have been proven to be infinite in some natural meaning: *Infinity is a mathematical rather than a physical notion which is used in mathematical calculations in order to find good estimates of physical values since the values can be measured with a nonzero error only*. From this ***finiteness assumption***, it follows that the *causal functions are exactly the mappings which are produced by a special class of automata, the letter-to-letter transducers* (or, *sequential machines*) *which transform input sequences $a={\left(a_{i}\right)}_{i\in N_{0}}$ of elementary causes into output sequences $b={\left(b_{i}\right)}_{i\in N_{0}}$ of elementary effects so that (ii) is satisfied* (cf., e.g., [19], a classical monograph on automata theory). Note that condition (ii) is just a Lipschitz condition with a constant 1 with respect to the natural non-Archimedean metric *d* on sequences. The metric *d* can be defined as follows: given two sequences, $c={\left(c_{i}\right)}_{i\in N_{0}}$ and $c^{\prime }={\left(c_{i}^{\prime }\right)}_{i\in N_{0}}$, over the same finite set, $d\left(c,c^{\prime }\right)=p^{-n}$, where $n=\max\{i\in N:c_{i}=c_{i}^{\prime }\}$ if such *n* exists, and $d(c,c^{\prime })=0$ if $c_{i}=c_{i}^{\prime }$ for all $i\in N_{0}$ (here p>1 is arbitrary real number). In this paper, we mostly consider the case when 𝒜 and ℬ are a finite *p*-element set $F_{p}$ where *p* is a prime number (the latter restriction is more a technical one imposed in order to not overload statements). This way, we may assume that $F_{p}$ is a finite *p*-element field and that the infinite sequences ${\left(c_{i}\right)}_{i\in N_{0}}$ (where $c_{i}\in F_{p}$) constitute the space $Z_{p}$ of *p*-adic integers under a natural one-to-one correspondence between the infinite sequences and canonical representations of *p*-adic integers $\sum_{i=0}^{\infty }c_{i}p^{i}$. In the case when p>1 is not a prime number, the sequences also may be put in one-to-one correspondence with the space $Z_{p}$ of *p*-adic integers since the latter spaces are defined for all p=2,3,4,…, and not necessarily only for prime *p*; see, e.g., [20].

Physical models, loosely speaking, describe functions *f* which are “physical laws” that express dependencies of physical quantities on other physical quantities; therefore, if time is one of these quantities, it is reasonable to assume causality, i.e., the functions *f* are causal. Let us express more formally the conditions the functions *f* must meet in order to be consistent both with Volovich postulated and ‘t Hooft causality postulate.

In order to be consistent with Volovich postulates, the following conditions should be satisfied.

- As only rational numbers can be measured, the *functions f, i.e., the closed forms of physical laws which can be experimentally verified, must be mappings of rational numbers to rational numbers; i.e., the functions f must take rational values when values of variables are rational*.
- In order to study functions *f* when values of variables are “very large” or ”very small” with respect to some reasonable metric, one has to expand the laws from the field of rational numbers Q to a bigger field which is complete with respect to that metric; therefore, this bigger field can only be the field of real numbers R and/or *p*-adic fields $Q_{p}$ for primes p=2,3,5,7,11,…; however, in order to be invariant with respect to the change of the number field, a *restriction to Q of any such expansion of f to a bigger field F⊃Q must be the same irrespective to which field F was used for in the expansion, whether F=R or $F=Q_{p}$*.

Further, to be consistent also with the ‘t Hooft causality postulate, the functions *f* should be causal; however, as it has been argued before, the "time" with respect to which the functions are causal must be order-isomorphic to a subset of Q. However, since, according to the Volovich postulates, no temporal interval smaller than Planck’s time can be measured, the temporal intervals can only be multiples of Planck’s time; therefore, the “time“ over which the functions *f* are causal must be order-isomorphic to a subset of Z. Thus, up to order isomorphism, the time scale is either $N_{0}=\left\{0,1,2,\ldots \right\}$ or Z={0,±1,±2,…} depending on whether the “beginning of time”’ exists or does not exist. According to the contemporary physical picture of the universe, it is reasonable to assume that the “beginning of time” exists; thus, the time scale must be $N_{0}$, up to order isomorphism. However, causal functions over the discrete time $N_{0}$ can be treated as *p*-adic 1-Lipschitz functions whose domain and range are *p*-adic integers $Z_{p}$ rather than the whole field $Q_{p}$, c.f., the reasoning which follows Definition 1; thus, as the “common part" of $Z_{p}$ and R (which we further denote via $Z_{p}\cap Q$) are *rational p-adic integers, i.e., the irreducible fractions, whose denominators are coprime to p*, to be consistent with Volovich postulates, the causal functions must take values from $Z_{p}\cap Q$ rather than from the whole Q; moreover, the functions must be expandable to the whole field R since $Z_{p}\cap Q$ is a dense subset of R. Finally, we can specify the formal properties the functions *f* must share in order to be consistent both with Volovich postulates and with the ‘t Hooft causality postulate, as follows:

**Condition** **1** (Complete consistency)**.** *A* *(univariate) continuous real function* f:R→R *which is consistent with both Volovich postulates and the ‘t Hooft causality postulate must share the properties listed below.*

(i) *For every prime p, the restriction ${f|}_{N_{0}}$ must be a causal function over discrete time $N_{0}$; i.e., the restriction ${f|}_{N_{0}}$ must satisfy a p-adic Lipschitz condition with a constant 1. That is, for all $m,n\in N_{0}$, there must hold the inequality*
  $$d_{p}{\left(f\right|}_{N_{0}}\left(m\right),f{|}_{N_{0}}\left(n\right))\leq d_{p}\left(m,n\right),$$
  *where $d_{p}$ is the p-adic metric.*
(ii) *Since $N_{0}$ is a dense subset in $Z_{p}$, by (i), for every prime p, there exists a unique extension of ${f|}_{N_{0}}$ to the function $f_{p}:Z_{p}\to Z_{p}$ which satisfies a Lipschitz condition with a constant of 1 with respect to the p-adic metric $d_{p}$. Therefore, to be invariant with respect to the change of the field, the function f:R→R must act on the set $Z_{p}\cap Q$ of all p-adic rational integers exactly as the function $f_{p}$ does; that is, for every prime p, the restriction ${f|}_{Z_{p}\cap Q}$ on rational p-adic integers $Z_{p}\cap Q$ must coincide with the restriction $f_{p}{|}_{Z_{p}\cap Q}$ on $Z_{p}\cap Q$:*
  $${f|}_{Z_{p}\cap Q}\left(r\right)=f_{p}{|}_{Z_{p}\cap Q}\left(r\right),\;\mathrm{for}\;\mathrm{all}\;r\in Z_{p}\cap Q.$$

**Note** **1.** *Condition 1 (ii) immediately implies that* $f\left(Z_{p}\cap Q\right)\subset Z_{p}\cap Q$ *for* all *prime* *p*; *thus, necessarily*

$$f\left(Z\right)\subset \underset{p\;\mathrm{prime}}{⋂}\left(Z_{p}\cap Q\right)=Z.$$

The questions which immediately arise are whether there exist functions *f* which satisfy the conditions, and if such functions do exist, what are these functions. In Section 4, we show that *functions which meet Conditions 1 do exist and constitute a class of all polynomials over Q of a special type* (the class contains, e.g., all polynomials over Z); see Theorem 5. Moreover, the functions turn out to be causal with respect to all finite alphabets and not necessarily with respect to *p*-symbol alphabets for prime *p*. This implies in particular that *the answer to the commonly asked question about p-adic mathematical physics concerning what p should be chosen by an experimenter in order to make the theory consistent with the observations is as follows: the choice of p is absolutely free if causality, discreteness at Planck’s scale, and invariance with respect to the change of the number field are assumed*.

We stress that the functions *f* which satisfy Conditions 1 are causal for all *p*-symbol alphabets and for all prime *p*, and hence, *for all finite alphabets*. In our view, the latter property appears to be too restrictive (and somewhat nonphysical, cf., the reasoning concerning the finiteness assumption above) since Planck’s scale includes a finite number of physical quantities (time, length, etc.) rather than an infinite number. Thus, it is reasonable to assume that Conditions 1 hold only for a finite set of primes; this implies that the functions *f* are causal with respect to finite alphabets, the prime power decompositions of the number of elements of which include only powers of primes from that set. The study of this class of functions can be reduced to cases containing only one prime, *p*. We show that if in the statement of Conditions 1, a prime *p* is fixed and that “for every prime *p*” is replaced by “for the prime *p*” then *there exist functions f that satisfy the Conditions, which are continuous real functions on R but which are not rational functions over Z; i.e., are not of the form u(x)/v(x) where u(x),v(x)∈Z[x] are polynomials with integer coefficients*; see Theorem 7. Note also that under such a restatement of the Conditions, f(Z) is *not necessarily* a subset of Z but only a subset of $Z_{p}\cap Q$, cf., Note 1.

## 3. Preliminaries

We review some notions and facts from *p*-adic analysis and from automata theory which will be needed further in the paper.

### 3.1. A Few Words about Words

An *alphabet* is just a finite nonempty set 𝒜; further in the paper, typically 𝒜={0,1,…,p−1}, where p>1 is an integer (mostly, but not always, *p* is a prime). Elements of 𝒜 are called *symbols*, or *letters*. By this definition, a *word of length n over alphabet 𝒜* is a finite sequence (stretching from right to left) $\alpha_{n-1}\cdots \alpha_{1}\alpha_{0}$, where $\alpha_{n-1},\ldots ,\alpha_{1},\alpha_{0}\in A$. The number *n* is called the *length* of the word $w=\alpha_{n-1}\cdots \alpha_{1}\alpha_{0}$ and is denoted via Λ(w). The *empty word* ϕ is a sequence of length 0; that is, the one that contains no symbols. Given a word $w=\alpha_{n-1}\cdots \alpha_{1}\alpha_{0}$, any word $v=\alpha_{k-1}\cdots \alpha_{1}\alpha_{0}$, k≤n, is called a *prefix* of the word *w*, whereas any word $u=\alpha_{n-1}\cdots \alpha_{i+1}\alpha_{i}$, 0≤i≤n−1 is called a *suffix* of the word *w*. Every word $\alpha_{j}\cdots \alpha_{i+1}\alpha_{i}$ where n−1≥j≥i≥0 is called a *subword* of the word $w=\alpha_{n-1}\cdots \alpha_{1}\alpha_{0}$. Given words $a=\alpha_{n-1}\cdots \alpha_{1}\alpha_{0}$ and $b=\beta_{k-1}\cdots \beta_{1}\beta_{0}$, the *concatenation* ab is the following word (of length n+k):

$$ab=\alpha_{n-1}\cdots \alpha_{1}\alpha_{0}\beta_{k-1}\cdots \beta_{1}\beta_{0}.$$

Given a word *w*, its *k*-times concatenation is denoted via ${\left(w\right)}^{k}$

$${\left(w\right)}^{k}=\underset{k\;\mathrm{times}}{\underset{︸}{ww\ldots w}}.$$

We denote using 𝒲=𝒲(𝒜) the set of all nonempty words over 𝒜={0,1,…,p−1} and using ${𝒲}_{\phi }$ the set of all words including the empty word ϕ. In the sequel, the set of all *n*-letter words over the alphabet 𝒜, we denote as ${𝒲}_{n}$; thus, $𝒲=\cup_{n=1}^{\infty }{𝒲}_{n}$. To every word $w=\alpha_{n-1}\cdots \alpha_{1}\alpha_{0}$, we put into the correspondence a non-negative integer $\mathrm{num}\left(w\right)=\alpha_{0}+\alpha_{1}\cdot p+\cdots +\alpha_{n-1}\cdot p^{n-1}$. Thus, num maps the set 𝒲 of all the nonempty finite words over the alphabet 𝒜 onto the set $N_{0}=\left\{0,1,2,\ldots \right\}$ of all non-negative integers. We will also consider a map ρ of the set *𝒲* into the real unit half-open interval [0,1); the map ρ is defined as follows: given $w=\beta_{r-1}\ldots \beta_{0}\in 𝒲$, put

(2) $$\rho \left(w\right)=\mathrm{num}\left(w\right)\cdot p^{-\Lambda (w)}=\frac{\beta_{0}+\beta_{1}p+\cdots +\beta_{r-1}p^{r-1}}{p^{r}}=0.\beta_{r-1}\ldots \beta_{0}\in \left[0,1\right).$$

We also use the notation 0. w for $0.\beta_{r-1}\ldots \beta_{0}$.

Along with finite words, we also consider one-side infinite words over the alphabet 𝒜; these are the infinite sequences of the form $\ldots \alpha_{2}\alpha_{1}\alpha_{0}$ where $\alpha_{i}\in 𝒜$, $i\in N_{0}$. In this paper, we may write one-side infinite words either stretching from left to right or from right to left when convenient, i.e., both $\alpha_{0}\alpha_{1}\alpha_{2}\ldots$ and $\ldots \alpha_{2}\alpha_{1}\alpha_{0}$ denote the same word. For finite words, we may also use both notations, left and right, and the order of indices of letters in the word shows which of the two notations is used. For infinite words, notions of prefix, suffix, and subwords are defined in the same way as they are for finite words; note that suffixes is are always infinite words whilst prefixes and subwords are always finite words. Let an infinite word *w* be eventually periodic; that is, let

$$w=\ldots \beta_{t-1}\beta_{t-2}\ldots \beta_{0}\beta_{t-1}\beta_{t-2}\ldots \beta_{0}\alpha_{r-1}\alpha_{r-2}\ldots \alpha_{0}$$

for $\alpha_{i}\beta_{j}\in 𝒜$; then, the subword $\beta_{t-1}\beta_{t-2}\ldots \beta_{0}$ is called a *period* of the word *w*, and the suffix $\alpha_{r-2}\ldots \alpha_{0}$ is called the preperiod of the word *w*. Note that a preperiod may be an empty word, while a period cannot. We ultimately write the periodic word *w* as $w={\left(\beta_{t-1}\beta_{t-2}\ldots \beta_{0}\right)}^{\infty }\alpha_{r-1}\alpha_{r-2}\ldots \alpha_{0}$.

### 3.2. p-adic Integers

We briefly recall some very basic facts about *p*-adic integers referring the reader to any monograph on *p*-adic analysis (e.g., to [20]) for deeper introduction to the subject. Let p>1 be an integer. A *p-adic integer*$z\in Z_{p}$ can be uniquely represented by a *canonical form* $z=\sum_{i=0}^{\infty }\zeta_{i}p^{i}$, where $\zeta_{i}\in \left\{0,1,\ldots ,p-1\right\}$, (i=0,1,2,…). Thus, to every infinite sequence $z={\left(\zeta_{i}\right)}_{i=0}^{\infty }$, we put into a correspondence a *p*-adic integer represented by a respective canonical form. The sequences z may also be treated as (one-side) infinite words over the alphabet {0,1,…,p−1}; thus, we now can expand a mapping num to the set $W_{p}$ of all infinite sequences over {0,1,…,p−1} so that $\mathrm{num}\left(z\right)=\sum_{i=0}^{\infty }\zeta_{i}p^{i}\in Z_{p}$. The so defined mapping $\mathrm{num}:W_{p}\to Z_{p}$ is one-to-one; thus, in what follows, we will not distinguish when necessary between *p*-adic integers, (one-side) infinite sequences over {0,1,…,p−1}, and infinite words over the alphabet {0,1,…,p−1}.

The sequences z which contain only finitely many nonzero terms correspond to non-negative integers from $N_{0}=\left\{0,1,2,\ldots \right\}$ represented by their base-*p* expansions; the sequences z which contain only finitely many terms not equal to p−1 correspond to negative integers −N={−1,−2,−3,…}. The sequences z which are ultimately periodic correspond to rational *p*-adic integers $z\in Z_{p}\cap Q$; i.e., to rational numbers which can be represented by irreducible fractions u/v whose denominators *v* are coprime to *p*. Any $z\in Z_{p}\cap Q$ can be represented as $z=c+d/(p^{t}-1)$ where c∈Z={0,±1,±2,…}, t∈N, $d\in \{0,1,\ldots ,p^{t}-2\}$.

The rational *p*-adic integers constitute a subring $Z_{p}\cap Q$ of $Z_{p}$ which is a dense subset of $Z_{p}$ with respect to the *p*-adic metric. The metric is induced by the *p*-adic absolute value ${\left|z\right|}_{p}$ which is equal to $p^{-{\mathrm{ord}}_{p}z}$, where ${\mathrm{ord}}_{p}z$ is the length of the longest zero-prefix (the prefix which consists of zeros only) of z if z≠0, and ${\left|0\right|}_{p}=0$ by definition.

Given n∈N={1,2,3,…} and a canonical expansion $z=\sum_{i=0}^{\infty }\alpha_{i}p^{i}$ for $z\in Z_{p}$, we further denote $z\;\mathrm{mod}\;p^{n}=\sum_{i=0}^{n-1}\alpha_{i}p^{i}\in N_{0}$. The mapping $\;\mathrm{mod}\;p^{n}:z\mapsto z\;\mathrm{mod}\;p^{n}$ can be treated as a ring epimorphism of $Z_{p}$ onto the residue ring $Z/p^{n}Z$, under a natural representation of elements of the residue ring by the least non-negative residues $\left\{0,1\ldots ,p^{n}-1\right\}$. Given n∈N, the base-*p* expansion of *n* is a finite word over $F_{p}$ whose length is $\lfloor \log_{p}n\rfloor +1$. As the base-*p* expansion of 0 is a one-letter word (namely, 0), in what follows, we assume that $\lfloor \log_{p}0\rfloor =0$. We stress that when considering words corresponding to numbers, for numbers 0,1,2,…, we distinguish their base-*p* expansions from their canonical *p*-adic representations: the latter are treated as infinite words rather than as finite words. We also stress that the mapping $\mathrm{num}:𝒲\to N_{0}$ is a surjection but not one-to-one, whereas the mapping $\mathrm{num}:W_{p}\to Z_{p}$ is one-to-one. In what follows, it always will be clear from the context what domain of num is considered.

A *probability measure* μ on $Z_{p}$ can be defined as follows: elementary μ-measurable sets are balls $B_{p^{-r}}\left(a\right)=\left\{b\in Z_{p}:b\equiv a\;\left(\mathrm{mod}\;p^{r}\right)\right)\}\subset Z_{p}$, where $a\in Z_{p}$, r∈N; put $\mu \left(B_{p^{-r}}\left(a\right)\right)=p^{-r}$. As the balls are simultaneously open and closed in topology induced by the *p*-adic absolute value ${\left|\cdot \right|}_{p}$ and as every two balls are either disjoint or one of them contains another one, the balls constitute a base of sigma-algebra which define a sigma-additive measure μ on $Z_{p}$. Actually, this measure μ is a Haar measure normalised so that $\mu (Z_{p})=1$. The measure μ is a Borel measure; that is, every open subset is μ-measurable (hence, every closed subset is μ-measurable as well). The measure μ is regular; that is, for any μ-measurable subset $A\subset Z_{p}$

$$\mu \left(A\right)=\sup\left\{\mu \left(S\right):S\subset A,S\;\mathrm{is}\;\mathrm{closed}\;\mathrm{in}\;Z_{p}\right\}=\inf\left\{\mu \left(S\right):S⊃A,S\;\mathrm{is}\;\mathrm{open}\;\mathrm{in}\;Z_{p}\right\}$$

Thus, $Z_{p}$ is a totally disconnected compact metric space whose metric is induced by the *p*-adic absolute value ${\left|\cdot \right|}_{p}$ and a probability space with respect to the measure μ. Note that the probability measure agrees with the metric; i.e., any function $Z_{p}\to Z_{p}$ that is continuous with respect to the metric is measurable: $f^{-1}\left(S\right)$ is μ-measurable once $S\subset Z_{p}$ is μ-measurable. Also note that the *p*-adic metric $d_{p}\left(a,b\right)={\left|a-b\right|}_{p}$ (where $a,b\in Z_{p}$) is *non-Archimdean*; that is, the triangle inequality holds for that metric in a stronger form:

$${\left|a-b\right|}_{p}\leq {\max\{|a-c|}_{p}{,|c-b|}_{p}\},\;\mathrm{for}\;\mathrm{all}\;a,b,c\in Z_{p}$$

In a similar way, the metric and probability measure can be defined for spaces $Z_{p}^{n}=\underset{n}{\underset{︸}{Z_{p}\times \cdots \times Z_{p}}}$, but in this paper, this *n*-dimensional space is mentioned only briefly in appropriate places; in order not to overload the exposition, we limit our “working space” to $Z_{p}$.

### 3.3. Systems, Transducers, Automata, Sequential Machines

Terminology in automata theory is somewhat diverse; in order to avoid a misunderstanding of the basic notions, we state them below.

**Definition** **2** (System, transducer, automaton, sequential machine)**.** *A (discrete)* system *(or a system with discrete time $N_{0}=\left\{0,1,2,\ldots \right\}$) is a 5-tuple A=〈ℐ,𝒮,𝒪,S,O〉 where*

- *ℐ is a nonempty finite set, the input alphabet;*
- *𝒪 is a nonempty finite set, the output alphabet;*
- *𝒮 is a nonempty (possibly, infinite) set of (epistemic) states;*
- *S:ℐ×𝒮→𝒮 is a state transition function;*
- *O:ℐ×𝒮→𝒪 is an output function.*

*The system is called* autonomous *if neither S nor O depend on input letters* (*that is, if S:𝒮→𝒮, O:𝒮→𝒪*); *otherwise, the system is called nonautonomous. A* subsystem *$A^{\prime }$ of A is a system $\langle ℐ,{𝒮}^{\prime },𝒪,S,O\rangle$ such that $\emptyset \neq {𝒮}^{\prime }\subset 𝒮$ and $S\left(\chi ,s^{\prime }\right)\in {𝒮}^{\prime }$ for all $\chi \in ℐ,s^{\prime }\in {𝒮}^{\prime }$. A subsystem is called* minimal *if it has no subsystems other than itself. An* initial automaton (*or in other terminology, a* letter-to-letter transducer *[21], a* Mealy sequential machine *[19], an* initial synchronous automaton *[22]*) *$A(s_{0})$ is a system where one of the states, $s_{0}\in 𝒮$, is fixed; $s_{0}$ is called the* initial state.

In what follows, the term *automaton* stands for an initial automaton; the subsystems of the automata are also called *subautomata*. A noninitial state s∈𝒮 is called *reachable* (or, *accessible*) if there exists a finite sequence $\chi_{0},\chi_{1},\ldots ,\chi_{N-1}\in ℐ$ such that $S(\chi_{N-1},s_{N-1})=s$, where $s_{i}=S\left(\chi_{i-1},s_{i-1}\right)$, i=1,2,…,N−1; i.e., if there exists a *path from the initial state $s_{0}$ to s* of finite *length N*.

An automaton A determines a unique map $f_{A}:\ldots \chi_{2}\chi_{1}\chi_{0}\mapsto \ldots \xi_{2}\xi_{1}\xi_{0}$ from the set W(ℐ) of all (one-side) infinite words over the alphabet *ℐ* to the set W(𝒪) of all (one-side) infinite words over the alphabet *𝒪*, as follows: at time instant i=0, the automaton, being in the state $s_{0}$, accepts the first input letter $\chi_{0}$, updates its state to a newer state $s_{1}=S\left(\chi_{0},s_{0}\right)$, and produces an output letter $\xi_{0}=O\left(\chi_{0},s_{0}\right)$; at the next time instant i=1, the automaton accepts $\chi_{1}$, updates its state to $s_{2}=S\left(\chi_{1},s_{1}\right)$, and produces an output letter $\xi_{1}=O\left(\chi_{1},s_{1}\right)$ etc. Therefore, $\xi_{i}=\varphi_{i}\left(\chi_{0},\ldots ,\chi_{i}\right)$, where $\varphi_{i}:{ℐ}^{i}\to 𝒪$ is a uniquely determined sequence of maps. The mapping $f_{A}$ is called an *automaton function* of the automaton A; clearly, the mapping is causal. It is well known that the converse is also true: *every causal mapping f:ℐ→𝒪 is an automaton function of a suitable automaton $A_{f}$* (see, e.g., [19] [Chapter IV, Theorem 8.2]). This is why for the rest of this paper we use the terms *causal function, automaton map, automaton function, automatic function, and 1-Lipschitz function* as synonyms.

For instance, take a prime number *p* and consider an automaton whose input (respectively, output) alphabet is *m*-tuple $\left(\alpha_{1},\ldots ,\alpha_{m}\right)\in F_{p}^{m}=ℐ$ (respectively, *n*-tuple from $F_{p}^{n}=𝒪$); then, the *automaton function is a map $Z_{p}^{m}\to Z_{p}^{n}$ which satisfies a Lipschitz condition with a constant 1*(further, 1-Lipschitz for brevity)*with respect to the p-adic metric* which is defined by the *p*-adic absolute value $|\left(z_{1},\ldots ,z_{k}\right){|}_{p}=\max\{|z_{1}{|}_{p},\ldots ,|z_{k}{|}_{p}\}$ on $Z_{p}^{k}$ (here $z_{j}=\sum_{i=0}^{\infty }\alpha_{ji}p^{i}\in Z_{p}$, $\alpha_{ji}\in F_{p}$, j=1,2,…,k). Moreover, *every 1-Lipschitz map $f:Z_{p}^{m}\to Z_{p}^{n}$ is an automaton function of a suitable automaton $A_{f}$*. Note that it is convenient sometimes to consider automata whose input/output alphabets’ cardinalities #ℐ, #𝒪 are *multiplicatively dependent* (i.e., such that #ℐ, #𝒪 are powers of some integer p>1) as automata having multiple inputs/outputs; i.e., to consider the 1-Lipschitz map $f:Z_{p}^{m}\to Z_{p}^{n}$ as an automaton function of an automaton having *m* input channels and *n* output channels, each channel over a *p*-symbol alphabet. That is, the automaton function in this case is a multivariate map over infinite words over a *p*-symbol alphabet. In what follows, we will refer to such a case as to *multivariate*.

It is clear that a composition of automaton functions is an automaton function of an automaton which is a sequential composition of respective automata. For automata (and for their functions), the Cartesian product and Kronecker product can also be defined, but we do not need these constructions within the scope of the current paper.

Given *f*, the automaton $A_{f}$ is not unique in the meaning of Definition 2:. There are infinitely many different automata (i.e., the ones whose sets of epistemic states are different, whose state transition functions are different, whose output functions are different) whose automaton function is *f*. Therefore, an *observer can only make guesses about the “internal structure“ of the system by observing pairs of “causes and effects”*, i.e., pairs (z,f(z)), $z\in Z_{M}$; moreover, the *equivalent states are indistinguishable for the observer*. However, given *f* there exists a unique automaton whose automaton function is *f* and whose set of states *𝒮* is the “smallest”. Call the two states $s_{i},s_{j}\in 𝒮$ of the automaton A *equivalent*; if whenever $s_{i},s_{j}$ are taken as initial states, the word mappings performed by either of the two initial automata are equal to one to another; i.e., if the input words are equal one to another, then the corresponding output words are also equal one to another. Factorising the state set of the automaton A by the equivalence relation, we obtain an automaton having no equivalent nonequal states whose automaton function is $f_{A}$. An automaton function $f_{A}$ is called *finite* if it can be produced by an automaton whose set of states is finite; that is, the factor set by the equivalence relation is finite.

**Figure 1:**
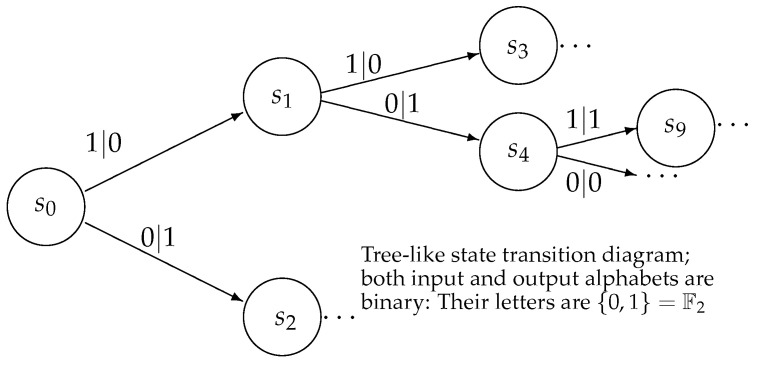
State transition diagram of a 2-adic automaton. Label α|β of the arrow that goes from the state $s_{i}$ to the state $s_{j}$ means that if the automaton is in the state $s_{i}$ and obtains α as the input symbol, it changes its state to $s_{j}$ and produces β as the output symbol.

It is convenient to represent automata by their *state transition diagrams* (or *Moore diagrams*), which are directed graphs (the *digraphs*) whose vertices are states and whose arrows are state transitions, with the arrows labelled by inputletter|outputletter. Given an automaton function $f:Z_{p}^{m}\to Z_{p}^{n}$, there exists an automaton whose automaton function is *f* and whose state transition diagram is an infinite tree such that each vertex (i.e., a state) has exactly $p^{m}$ outgoing arrows which go to $p^{m}$ different vertices, cf., Figure 1 which depicts a state transition diagram of an automaton whose automaton function is $f:Z_{2}\to Z_{2}$.

**Figure 2:**
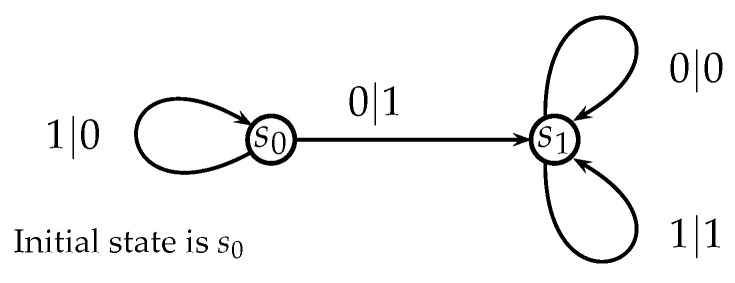
Reduced state transition diagram of the 2-adic odometer.

The automaton function of the automaton whose state transition diagram is depicted by Figure 1 is f(z)=z+1 ($z\in Z_{2}$), the 2-adic *odometer*. The *reduced* state transition diagram (which is obtained by factorisation with the equivalence relation defined earlier) is a digraph having only two vertices, cf., Figure 2. The automaton whose state transition diagram is depicted as in Figure 2 has the same automaton function f(z)=z+1 on $Z_{2}$; thus, the function *f* is a finite automaton function since it is produced by an automaton having only two states. Note that a *finite automaton is minimal if and only if its state transition diagram is a strongly connected digraph*; i.e., given any two vertices, there is a path connecting the vertices. The 2-adic odometer, therefore, has the only minimal subautomaton, the one whose set of states consists of the only state $s_{1}$.

Recall that a *path* in a digraph is a (finite or infinite) sequence of arrows ${\vec{a}}_{0},{\vec{a}}_{1},\ldots$ such that for every pair ${\vec{a}}_{j},{\vec{a}}_{j+1}$ of the arrows there is a state *s* such that the arrow ${\vec{a}}_{j}$ goes to *s* and ${\vec{a}}_{j+1}$ goes from *s*. In a state transition diagram of an automaton having input alphabet 𝒜, to every path there corresponds a word $\chi_{0}\chi_{1}\ldots$ over 𝒜 where $\chi_{j}$ are input letters, the ones which occupy the first positions in the label α|β of the arrow: if 𝒜={0,1,…,p−1} then to every path ${\vec{a}}_{0}{\vec{a}}_{1}\ldots$ that starts from the initial state $s_{0}$, there corresponds the *p*-adic integer $\chi_{0}+\chi_{1}p+\cdots \chi_{k-1}p^{k-1}+\cdots$ where $\chi_{j}|\cdot$ is a label which marks the arrow ${\vec{a}}_{j}$, j=0,1,2,…. Simply speaking, the word $\chi_{0}\chi_{1}\ldots$ is an input word such that when an automaton is fed by that word, the automaton updates it states $s_{0}\to s_{1}\to s_{2}\to \cdots$ where $s_{j}$ is a state from which the arrow ${\vec{a}}_{j}$ starts and $s_{j+1}$ is a state to which the arrow ${\vec{a}}_{j}$ goes; thus, the states $s_{j},s_{j+1}$ are connected by the arrow ${\vec{a}}_{j}$ which goes from $s_{j}$ to $s_{j+1}$ and which is labelled as $\chi_{j}|\cdot$.

The statement of the following proposition is well known; see, e.g., [10]:

**Proposition** **1** (Finite and nonfinite automata functions)**.** *Both addition $+:Z_{p}^{2}\to Z_{p}$ and multiplication $\cdot :Z_{p}^{2}\to Z_{p}$ are automata functions; addition is a finite automaton function, whereas multiplication is not. A constant map $f:Z_{p}\to Z_{p}$ is a finite automaton function if and only if $f\left(z\right)=const\in Z_{p}\cap Q$ for all $z\in Z_{p}$. An affine map f(z)=az+b, $\left(z\in Z_{p}\right)$ is a finite automaton function if and only if $a,b\in Z_{p}\cap Q$.*

Automata functions of automata whose input/output alphabets are $F_{p}$ can be explicitly represented via *Mahler series*. Recall that if p>1 is an integer (which is not necessarily a prime), then every function $f:N_{0}\to Z_{p}$ (or, respectively, $f:N_{0}\to Z$) has the only *Mahler expansion*; that is, has a unique representation via the so-called *Mahler* (*interpolation*) *series* [20]:

(3) f(x)=∑i=0∞aixi,

where $a_{i}\in Z_{p}$ (respectively, $a_{i}\in Z$), i=0,1,2,…, and

xi=x(x−1)⋯(x−i+1)i!

for i=1,2,…;

x0=1,

by definition. The following reciprocity relations hold:

(4) ai=∑j=0i(−1)jijf(i−j),i=0,1,2,…

The function $f:Z_{p}\to Z_{p}$ represented by series (3) is continuous with respect to the *p*-adic metric if and only if $a_{i}$ tends *p*-adically to 0 as *i* tends to infinity.

To represent functions of several variables, one may use interpolation series of the following form:

(5) f(x1,…,xn)=∑(i1,…,in)∈N0nai1,⋯,inx1i1x2i2⋯xnin;

Here, $a_{i_{1},\ldots ,i_{n}}\in Z_{p}$. As the map $f:Z_{p}^{n}\to Z_{p}$ is an automaton function (of the automaton having *n* inputs and one output over a *p*-symbol alphabet $F_{p}$), the following Theorem 1 completely describes the automaton functions. Note that $\left\lfloor \log_{p}i\right\rfloor$ is the smallest integer which does not exceed $\log_{p}i$; thus, $\left\lfloor \log_{p}i\right\rfloor$ is reduced by 1 number of digits in the base-*p* expansion of $i\in N_{0}$; thus, $\lfloor \log_{p}0\rfloor =0$.

**Theorem** **1** ([23] [Theorem 3.53])**.** *A function $f:Z_{p}^{n}\to Z_{p}$ represented by the Mahler expansion (5) is 1-Lipschitz (with respect to the p-adic metric) if and only if*

$$|a_{i_{1},\ldots i_{n}}{|}_{p}\leq p^{-\nu (i_{1},\ldots ,i_{n})},$$

*where $\nu \left(i_{1},\ldots ,i_{n}\right)=\max\left\{\left\lfloor \log_{p}i_{k}\right\rfloor :k=1,2,\ldots ,n\right\}.$*

*In particular, a univariate function $f:Z_{p}\to Z_{p}$ represented by the Mahler expansion (3) is 1-Lipschitz if and only if*

$$|a_{i}{|}_{p}\leq p^{-\lfloor \log_{p}i\rfloor }$$

*for all i=1,2,…. In other words, a function $f:Z_{p}\to Z_{p}$ is automatic if and only if it can be represented as*

(6) f(x)=∑i=0∞ciplogpixi,

*for suitable $c_{i}\in Z_{p}$; i=0,1,2,….*

**Note** **2.** *The series (6) converges uniformly on* $Z_{p}$. *Given a 1-Lipschitz function*$f:Z_{p}\to Z_{p}$*, the representation (2) is unique.*

There are explicit representations of automaton functions in other terms (e.g., via van der Put series, digital derivatives) which are not needed within the scope of the paper; an interested reader is referred to an expository paper [24]. Additionally, it is worth noting that *Moore sequential machines* are initial automata whose output function depends only on states, cf. Definition 2, but it is well known that the latter machines are equivalent to Mealy machines in the following meaning: *under the assumption that an output of a Moore machine at initial state is an empty symbol (i.e., no output), then the classes of causal functions represented by Mealy machines and by Moore machines coincide*; however, to represent a causal function via a state transition diagram of a Moore machine, one needs more states compared to the diagram of the respective Mealy machine. This is why in the rest of the paper, the example state transition diagrams are given for Mealy machines although, from a physical point of view, it might be more natural to deal with Moore machines since they appear to be defined on Markov chains whilst Mealy machines are not, as the output of Moore machines formally depends only on states rather than on arrows reaching the states; however, this view is misleading since Mealy machines do exactly what Moore machines do.

Finally, automaton functions is the concept which illuminates the sharp difference between the two approaches, the ‘t Hooft’s one based on cellular automata and ours based on letter-to-letter transducers: *the class of functions computed by the transducers is much smaller than the class of functions computed by cellular automata*. To exemplify this, consider one more type of transducer, the *letter-to-word transducer* (or, *asynchronous initial automata*, [22]) whose output function is $ℐ\times 𝒮\to {𝒲}_{\phi }$ rather than ℐ×𝒮→𝒪 and where ${𝒲}_{\phi }$ is the set of all finite words (including the empty word ϕ) over the output alphabet *𝒪*, c.f., Definition 2. In the case when ℐ={0,1,…,p−1}, an asynchronous initial automaton, produces a map $Z_{p}\to Z_{p}$ that can be constructed by an analogy with the synchronous case; then, the maps $Z_{p}\to Z_{p}$, which are automaton functions of nondegenerate synchronous initial automata, constitute the class of all functions that are continuous with respect to the *p*-adic metric, c.f., [22] [Theorem 2.4]. Therefore, these functions are defined by the maps $N_{0}\to N_{0}$ as $N_{0}$ is dense in $Z_{p}$ with respect to the *p*-adic metric. The automata functions of initial synchronous automata are all of the form (6), so if *f* is an automaton function of a synchronous automaton such that $f:N_{0}\to N_{0}$, then necessarily $c_{i}p^{\left\lfloor \log_{p}i\right\rfloor }\in Z$ for all $i\in N_{0}$ as the value of $a_{i}$ for every *i* can be calculated by using (4). Therefore, from the algorithmic point of view, *f* is a *primitive recursive function*. In a similar way, it can be shown that the functions $N_{0}\to N_{0}$ which are automaton functions of nondegenerate asynchronous automata are also a primitive recursive function since they can be uniquely expanded to continuous *p*-adic functions $Z_{p}\to Z_{p}$ and thus are of the form (3). However, a class of cellular automata is Turing-complete; therefore, the automaton functions of cellular automata (which can be defined for these automata as well) constitute the class of all *general recursive functions*; hence, they are not even everywhere defined on $N_{0}$, let alone *p*-adic continuity or 1-Lipschizness. In other words, one may say that the class of automata functions of initial synchronous automata is the smallest class of automata functions, whereas the class of automata functions of cellular automata is the largest one.

### 3.4. On the Dynamics of Causal Functions

Here, we briefly recall some facts about the dynamics of automaton functions following [23]; i.e., on the dynamics of the *p*-adic 1-Lipschitz functions. The dynamics arises quite naturally since the automaton function of a sequential composition of automata is a composition of automaton functions. In addition, we recall from [25] a few general notions and facts from dynamical system theory which will be needed in subsequent steps.

A map F:S→Y from a measure space S into a measure space Y endowed with probability measures μ and ν, respectively, is said to be *measure-preserving* if $\mu (F^{-1}\left(S\right))=\nu \left(S\right)$ for each measurable subset S⊂Y; in the case when S=Y and μ=ν, a measure-preserving map *F* is said to be *ergodic* if given a measurable subset *S* such that $F^{-1}\left(S\right)=S$, either μ(S)=1 or μ(S)=0; the map *F* is called *weak mixing* if for any two measurable sets A,B, there exists a sequence $n_{k}\to \infty$ over $N_{0}$ such that $\mu (F^{-n_{k}}\left(A\right)\cap B)\to \mu \left(A\right)\mu \left(B\right)$ as k→∞. If $n_{k}=k$, the weak mixing is called strong mixing. Weak mixing implies ergodicity but is a stronger condition than is ergodicity: the map *F* is weak mixing if and only if the map (x,y)↦(F(x),F(y)) of S×S into S×S is ergodic.

**Example** **1** (Trivial although important)**.** *Let S be a finite set, #S=N, which is endowed with a uniform probability measure μ: given A⊂S, #A=M, we put $\mu \left(A\right)=\frac{M}{N}$. A transformation f on S is measure-preserving if and only if f is bijective, i.e., if f is a permutation on S. The map f is ergodic if and only if this permutation consists of a single cycle, i.e., if it is* transitive *on S.*

**Definition** **3** (Topological transitivity)**.** *Given a topological space X and a continuous mapping f:X→X, the mapping f (as well as the respective dynamical system) is called* topologically transitive *if there exists a dense orbit of f; that is, if there exists x∈X such that the set of iterations $\left\{f^{i}\left(x\right)\;i\in N_{0}\right\}$ is everywhere dense in X. A dynamical system is called* minimal *if every orbit is dense.*

There is another (generally, nonequivalent to the above) definition of topological transitivity: the map *f* is called topologically transitive if for every pair of nonempty open sets U,V⊂X, there exists a non-negative integer *ℓ* such that $f^{\ell }\left(U\right)\cap V\neq \emptyset$. However, as in the sequel we deal with spaces $X=Z_{p}^{n}$, n∈N, the two definitions are equivalent since the spaces have no isolated points and are separable and of second category.

**Definition** **4** (Unique ergodicity)**.** *A mapping f:S→S is called* uniquely ergodic *if there exists a unique f-invariant probability measure μ on S; i.e., such that f is ergodic with respect to μ.*

**Proposition** **2** ([25] [Corollary 4.3.6])**.** *A minimal isometry of a compact metric space is uniquely ergodic.*

Given a 1-Lipschitz function $f:Z_{p}\to Z_{p}$, a map $f\;\;\mathrm{mod}\;p^{k}:z\mapsto f\left(z\right)\;\mathrm{mod}\;p^{k}$ is a well-defined map of the residue ring $Z/p^{k}Z$ into itself, cf., Section 3.2. This map is called an *induced function modulo $p^{k}$*. The function induced modulo $p^{k}$ by a 1-Lipschitz function $F:Z_{p}^{n}\to Z_{p}^{n}$ can be defined by analogy.

**Definition** **5** (Bijectivity and transitivity modulo $p^{k}$)**.** *A 1-Lipschitz function $F:Z_{p}^{n}\to Z_{p}^{n}$ is said to be a* bijective modulo $p^{k}$*(respectively,* a transitive modulo $p^{k}$*) whenever the induced function $F\;\;\mathrm{mod}\;p^{k}:{\left(Z/p^{k}Z\right)}^{n}\to Z/p^{k}{Z)}^{n}$ is bijective (respectively, transitive).*

In what follows, if the measure is not specified explicitly, measure preservation and ergodicity are defined with respect to the Haar probability measure on $Z_{p}^{n}$, cf., Section 3.2. The following Theorem and Proposition are proven in [23] [Chapter 4].

**Theorem** **2** (Main ergodic theorem for 1-Lipschitz *p*-adic dynamics)**.** *A 1-Lipschitz function $F:Z_{p}^{n}\to Z_{p}^{n}$ is measure-preserving *(*or, accordingly, ergodic*) *if and only if it is bijective,* (*or, accordingly, transitive*) *modulo $p^{k}$ for all k=1,2,3,….*

**Proposition** **3.** *A function $F:Z_{p}^{n}\to Z_{p}^{n}$ is measure-preserving and 1-Lipschitz if and only if it is an isometry of $Z_{p}^{n}$ onto itself. A measure-preserving 1-Lipschitz function F is ergodic if and only if it has a dense orbit; moreover, all orbits of ergodic 1-Lipschitz function $F:Z_{p}^{n}\to Z_{p}^{n}$ are dense.*

The space $Z_{p}^{n}$ is a probability space and a metric (and thus topological) space. Therefore, for a continuous function $Z_{p}^{n}\to Z_{p}^{n}$, one can define a *metric entropy* (related to the probability) and a *topological entropy* (related to the topology). In general, given *F*, these entropies may differ. However, *for 1-Lipschitz functions F, both entropies coincide and are 0*. Indeed, it is known that if G:X→X is an isometry of a compact metric space X onto itself, then the topological entropy of *G* is 0, cf., e.g., [26] [Exercise 6.3]. Yet, the variational principle for the topological entropy necessitates that the topological entropy of a continuous transformation *G* of a compact metric space X is a supremum of all metric entropies of *G* with respect to *G*-invariant measures on X, cf., [26] [Theorem 6.8.1]; this proves the claim. Moreover, from Proposition 3 it follows that given a 1-Lipschitz ergodic map $F:Z_{p}\to Z_{p}$, the map F×F:(x,y)↦(F(x),F(y)) of $Z_{p}^{2}$ to $Z_{p}^{2}$ is never ergodic since an orbit which starts from $\left(z,z\right)\in Z_{p}^{2}$ is never dense in $Z_{p}^{2}$, so *F is never weak mixing*.

We summarize as follows:

- A function $F:Z_{p}^{n}\to Z_{p}^{n}$ is measure-preserving and 1-Lipschitz if and only if it is isometric.
- A 1-Lipschitz function $F:Z_{p}^{n}\to Z_{p}^{n}$ is isometric if and only if it is bijective; i.e., if and only if the respective automaton is *time-reversible*: an automaton A whose automaton function is *F* is called *time-reversible* if there exists an automaton B whose automaton function is *G* and such that $G=F^{-1}$, i.e., the composition G(F) is an identity map $Z_{p}^{n}\to Z_{p}^{n}$. The time-reversibility is also called *automaton weak invertibility*, 27].
- All 1-Lipschitz functions $Z_{p}^{n}\to Z_{p}^{n}$ have zero topological entropy (thus, zero metric entropy).
- All 1-Lipschitz ergodic maps $F:Z_{p}^{n}\to Z_{p}^{n}$ are uniquely ergodic.
- None of the 1-Lipschitz ergodic maps $F:Z_{p}^{n}\to Z_{p}^{n}$ is weak mixing.
- Every orbit of every 1-Lipschitz ergodic map $F:Z_{p}^{n}\to Z_{p}^{n}$ is dense.

When n=1 the following is true [28] [Theorem 6]:

**Theorem** **3.** *Let $f:Z_{p}\to Z_{p}$ be surjective and 1-Lipschitz. The following propositions are equivalent:*

(i) *f is minimal;*
(ii) *f is conjugate to the translation τ:x↦x+1 on $Z_{p}$;*
(iii) *f is uniquely ergodic;*
(iv) *f is ergodic.*

In subsequent steps, we will need the following sufficient conditions of measure- preservation/ergodicity for 1-Lipschitz functions $Z_{p}\to Z_{p}$, [23] [Lemma 4.41]:

**Lemma** **1.** *Given a 1-Lipschitz function $f:Z_{p}\to Z_{p}$ and p-adic integers c, d, c≢0(modp), the function g(x)=d+cx+p·f(x) is 1-Lipschitz measure-preserving and the function h(x)=c+x+p·Δf(x) is 1-Lipschitz ergodic. *(Here, Δ is a difference operator Δf(x)=f(x+1)−f(x) by definition.)

## 4. Completely Consistent Functions

Causal functions over discrete time $N_{0}=\left\{0,1,2,\ldots \right\}$ from Section 2 are the maps *f* from the set W(ℐ) of all infinite words over a finite alphabet *ℐ* to the set W(𝒪) of all infinite words over a finite alphabet *𝒪* which are 1-Lipschitz with respect to standard non-Archimedean metric *d* on the words, d(f(u),f(v))≤d(u,v), or, which is the same if and only if words f(u) and f(v) have a common prefix of length at least *k* whenever respective words u and v have a common prefix of length *k*.

As a composition of automaton functions is an automaton function, the following example introduces an important class of functions which are automaton functions for every *p* (by Proposition 1) and, moreover, which at the same time can be considered as continuous real functions.

**Example** **2** (Polynomials over Z are automata functions)**.** *A polynomial map f:z↦f(z) where f(x)∈Z[x] is an automaton function; f is never a finite automaton function if degf≥2.*

That is, as the set $Z_{p}\cap Q$ of all rational *p*-adic integers is dense both in $Z_{p}$ with respect to the *p*-adic metric on $Z_{p}$ for every *p* and with respect to usual real metric on R, the map induced by a polynomial f∈Z[x] is well-defined both on $Z_{p}$ for all *p* and on R; i.e., the map f:z↦f(z), $\left(z\in Z_{p}\cap Q\right)$, can be uniquely extended both to continuous maps f:u↦f(u), ($u\in Z_{p}$), for all *p*, and to a continuous map f:y↦f(y), (y∈R). This is because any polynomial map is a composition of additions and multiplications, and these operations are well-defined and continuous both on all $Z_{p}$ and on R with respect to corresponding metrics and agree on $Z_{p}\cap Q$.

### 4.1. Universally Causal Functions

The maps $f:N_{0}\to Z$ defined by polynomials over Z are examples of functions which we call *universally causal*; these are the functions which, loosely speaking, are causal with respect to all finite alphabets 𝒜 and ℬ such that #𝒜=#ℬ=r for whatever r∈{2,3,4,…} is taken. Here is a formal definition.

**Definition** **6** (Universally causal functions)**.** *A causal function $f:{\left(a_{i}\right)}_{i=0}^{\infty }\mapsto {\left(\varphi_{i}^{f}\left(a_{i}\right)\right)}_{i=0}^{\infty }$ whose domain is all sequences $a={\left(a_{i}\right)}_{i=0}^{\infty }$ over 𝒜 and whose codomain is all sequences $b={\left(b_{i}\right)}_{i=0}^{\infty }$ over ℬ (see Section 1) is called* universally causal *if #𝒜=#ℬ=r>1, and there exist bijections α:𝒜↔{0,1,…,r−1} and β:ℬ↔{0,1,…,r−1} such that the induced map $\tilde{f}:Z_{r}\to Z_{r}$ defined by $\tilde{f}:\sum_{i=0}^{\infty }\alpha \left(a_{i}\right)r^{i}\mapsto \sum_{i=0}^{\infty }\beta \left(\varphi_{i}^{f}\left(a_{0},\ldots ,a_{i}\right)\right)r^{i}$, c.f., (1), satisfies the following conditions:*

(i) *$\tilde{f}\left(N_{0}\right)\subset Z$, where $N_{0}$, the rational non-negative integers, are all r-adic integers whose canonical r-adic representations contain only a finite number of nonzero terms; and Z, the rational integers, are either non-negative rational integers or negative rational integers. The latter are all r-adic integers whose canonical r-adic representations contain only a finite number of terms other than $\left(r-1\right)r^{i}$.*
(ii) *$\tilde{f}\left(m\right)\equiv \tilde{f}\left(n\right)\;\left(\mathrm{mod}\;q\right)$ once m≡n(modq), where $m,n,q\in N_{0}$, q>1.*

The class of universally causal functions is much wider than than that of functions defined by polynomials over Z. Actually, up to the bijections α, β, the universally causal functions constitute a class of the so-called *pseudo-polynomials*, Ref. [29] or *universal functions* [30]; these are maps $g:N_{0}\to Z$ which satisfy (ii) from Definition 6.

**Theorem** **4** (On pseudo-polynomials)**.** *A map $g:N_{0}\to Z$ is a pseudo-polynomial if and only if g can be represented as*

(7) g(z)=c0+∑i=1∞ci·lcm{1,2,…,i}·zi=c0+∑i=1∞ci·eψ(i)·xi,

*where $c_{i}\in Z$, lcm{1,2,…,i} is the least common multiple of the numbers 1,2,…,i, and $\psi \left(i\right)=\sum_{q\leq i,\;q\;\mathrm{prime}}\left\lfloor \log_{q}i\right\rfloor \ln q$ is the second Chebyshev function, i=1,2,…* (recall that ψ(i)=i+o(i)).

In the literature, often only the functions of the form (7) which are not polynomials are called pseudo-polynomials, but in the current paper, we call pseudo-polynomials all functions of that form. The class of pseudo-polynomials is wide and is a subject of study for a number theorists, who focus mostly on Ruzsa’s conjecture, which is about the sufficient conditions for when a pseudo-polynomial is a polynomial; see, e.g., [31]. Classical examples of pseudo-polynomials which are not polynomials are $\sum_{i=0}^{\infty }x^{\underline{i}}$ and $\sum_{i=0}^{\infty }{\left(-1\right)}^{i}x^{\underline{i}}$, where $x^{\underline{i}}$ is the *i*-th falling factorial power, $x^{\underline{i}}=x\left(x-1\right)\cdots \left(x-i+1\right)$, if i>0 and $x^{\underline{0}}=1$.

**Note** **3.** *The following is noteworthy.*

- *Even if all but a finite number of* $c_{i}$ *in (7) are 0, i.e., if g is a polynomial, then g is not necessarily a polynomial with integer coefficients, although g is polynomial over* Q. *For instance, put* $c_{4}=1$ *and put* $c_{i}=0$ *for* i≠4.
- *If all but a finite number of* $c_{i}$ *are 0, the function* *g is well-defined on* R*; that is, f can be uniquely expanded to a map* R→R *which is continuous with respect to the real metric.*
- *For every* p>1*, the map g can be uniquely expanded to 1-Lipschitz (thus, automatic) map* $Z_{p}\to Z_{p}$*, cf., (6) from Theorem 1.*

### 4.2. The Main Theorem on Complete Consistency

Therefore, polynomials of the form (7) satisfy Conditions 1 for every prime *p*. It turns out that the converse statement is also true. Note that a function which satisfies the conditions for all prime *p* must be universally causal, i.e., it must be a pseudo-polynomial; however, the only pseudo-polynomials which are well-defined on R are polynomials since if an infinite number of $c_{i}$ in (7) are nonzero then the series diverges at; for example, z=−1 as the common term at z=−1 is ${\left(-1\right)}^{i}c_{i}\cdot \mathrm{lcm}\left\{1,2,\ldots ,i\right\}$ and thus does not go to 0 as i→∞. However, this argument does not prove the converse claim since, for instance, if *g* is a pseudo-polynomial which is not a polynomial, then the composition $g(z^{2})$ is also a pseudo-polynomial, but the map $z\mapsto g(z^{2})$ is well-defined on Z. Nonetheless, the following theorem holds true.

**Theorem** **5** (Functions which satisfy Conditions 1)**.** *A continuous function f:R→R satisfies Conditions 1 if and only if f is a polynomial of the form (7); i.e., when all but a finite number of $c_{i}$ in (7) are zero.*

**Proof.**  According to Theorem 4, every polynomial *g* over Q of the form (7) satisfies (i) from Conditions 1, cf., (ii) of Definition 6. Therefore, *g* also satisfies (ii) from Conditions 1 since g(Q)⊂Q as *g* is a polynomial over Q.

To prove the converse claim, note that the map $u:Z_{p}\to Z_{p}$ is 1-Lipschitz if and only if $\Delta^{i}u\left(z\right)/i\in Z_{p}$ for all $z\in Z_{p}$ and all i∈N, cf., [23] [Proposition 3.38] or [32] [Proposition 3.1]. Here, Δ is the (forward) difference operator, i.e., $\Delta^{1}u\left(z\right)=\Delta u\left(z\right)=u\left(z+1\right)-u\left(z\right)$, $\Delta^{i+1}u\left(z\right)=\Delta \left(\Delta^{i}u\left(z\right)\right)$. Therefore, we have the following:

(8) $$\frac{\Delta^{i}f\left(z\right)}{i}\in Z,\;\mathrm{for}\;\mathrm{all}\;z\in Z,i\in N.$$

Further, from (ii) of Conditions 1, it follows (by Note 1) that $\frac{1}{h}\cdot \left(f\left(z+h\right)-f\left(z\right)\right)=q\left(z,h\right)\in Q$ for all z,h∈Q and h≠0 since $z,h\in Z_{p}\cap Q$ for all but not more than a finite number of primes *p*. However, f(z+h)=∑i=0∞hiΔif(z) where the series converges *p*-adically for all but not more than a finite number of primes *p* as $\Delta^{i}f\left(z\right)$ tends *p*-adically to 0 according to Theorem 1; cf., (i) of Conditions 1. Thus, the series converges to some $q^{\prime }\left(z,h\right)\in Q$ by (ii) of Conditions 1, and, therefore, the series converges in R to that rational number $q^{\prime }\left(z,h\right)$. We have

(9) 1h(f(z+h)−f(z))=1h∑i=1∞hiΔif(z)=∑i=1∞h−1i−1Δif(z)i,

where the series in the right hand part converges in R to the rational number q(z,h)∈Q; therefore, the absolute value |h−1i−1Δif(z)i| must tend to 0 in R as i→∞. Represent the following:

h−1i−1=h1−1h2−1⋯hi−1−1

From here, it follows that

h−1i−1≥1−hi−1i−1>0foralli=2,3,…;−1<h<0

As for −1<h<0 rational, it holds

$$\lim_{i\to \infty }{\left|1-\frac{h}{i-1}\right|}^{i-1}=e^{-h}>0,$$

from the convergence of the series in the right hand part of (9), and it it follows necessarily that $\lim_{i\to \infty }\frac{\Delta^{i}f\left(z\right)}{i}=0$; therefore, according to (8), given z∈Z, then $\Delta^{i}f\left(z\right)=0$ for all sufficiently large *i*. In particular, $\Delta^{i}f\left(0\right)=0$ for all sufficiently large *i*. As f(x)=∑i=0∞xiΔif(0) and, in view of Conditions 1 (i), the series in the right hand part converges *p*-adically in $Z_{p}$, then, according to Note 2, we finally conclude that *f* is a polynomial over Q; hence, a polynomial of the form (7). □

**Definition** **7** (Totally consistent functions)**.** *Further in the paper, functions described by Theorem 5 are called* totally consistent; *𝒞(R) denotes the class of all totally consistent functions.*

**Note** **4.** *In view of Theorem 1, the statement of Theorem 5 holds true for continuous real functions* $R^{m}\to R^{n}$ *as well. The proof is a minor modification of the proof of the said theorem and thus is omitted.*

**Note** **5.** *From the proof of Theorem 5, it follows that relaxation of Conditions 1 to functions f whose domain contains a real interval rather than coincides with the whole* R *does not widen the class of functions.*

### 4.3. The Free Choice of Discreteness/Continuity

We stress once again that *in the measurement of values of physical quantities, the rational p-adic integers $Z_{p}\cap Q$ are indistinguishable from rational numbers Q since every real number can be approximated by a rational p-adic integer with any desirable accuracy*. Note also that polynomials over Z are totally consistent; cf. Example 2. The theorem by M. I. Chlodovsky states that a continuous real-valued function on a real interval which does not contain integers can be uniformly approximated by polynomials over Z [33,34]. Therefore, according to Theorem 5, *any continuous real function on the real interval [α,β] where 0<α<β<1 can be uniformly approximated* (with respect to a real metric) *by completely consistent functions*, i.e., by functions from ${𝒞}_{\mathrm{all}\;\mathrm{primes}}\left(R\right)$. On the other hand, Theorems 1 and 5 imply that *any p-adic 1-Lipschitz function $f:Z_{p}\to Z_{p}$ can be uniformly approximated* (with respect to the *p*-adic metric) *by completely causal functions, regardless of which prime p is taken*.

Indeed, according to (6), the function *f* can be represented by the Mahler expansion f(z)=∑i=0∞bip⌊logpi⌋zi where $b_{i}\in Z_{p}$. According to Theorem 5, given n∈N, we must find a polynomial g(x)=c0+∑i=1∞ci·lcm{1,2,…,i}·xi where all $c_{i}\in Z$ such that $f\left(z\right)\equiv g\left(z\right)\;\left(\mathrm{mod}\;p^{n}\right)$ for all $z\in Z_{p}$. As $\mathrm{lcm}\left\{1,2,\ldots ,i\right\}=\prod_{\left(q\right)}q^{m_{i,q}}$ where $q^{m_{i,q}}$ is the largest power of a prime *q* that does not exceed *i*, then $m_{i,q}=\left\lfloor \log_{q}i\right\rfloor$, and therefore $c_{i}\cdot \mathrm{lcm}\left\{1,2,\ldots ,i\right\}=c_{i}p^{\left\lfloor \log_{p}i\right\rfloor }a_{i}$ where $a_{i}=\mathrm{lcm}\left\{1,2,\ldots ,i\right\}/p^{\left\lfloor \log_{p}i\right\rfloor }$ is in Z and is coprime to *p*. Hence, given $b_{i}\in Z_{p}$, a congruence $b_{i}\equiv c_{i}a_{i}\;\left(\mathrm{mod}\;p^{n}\right)$ has an integer solution $c_{i}\in Z$. Put $c_{i}=0$ for all *i* such that $\lfloor \log_{p}i\rfloor \geq n$, and let $c_{0}\in Z$ be the least non-negative residue of $b_{0}\in Z_{p}$ modulo $p^{n}$. Then, the so-defined polynomial *g* is the one we need.

All the considerations already outlined in this paper may be taken as evidence in favour of the following plausible statement which answers the question to which the whole book [2] is devoted:

**Interpretation** **1** (Observer’s free choice of discreteness/continuity)**.** *Due to the inevitable nonzero error in the measurements of values of physical quantities, an observer’s conclusion whether Nature on the smallest of the scales is discrete or continuous completely depends on the observer’s free choice of metric with respect to which the observer processes the measured numerical data. Moreover, the very “degree of the discreteness”, the number* *p, is subject to observer’s free choice.*

### 4.4. The Free Choice of Chaoticity/Predictability

The next important question which should be addressed is related to the ‘t Hooft causality postulate and can be posed as follows: Can an observer determine through numerical observational data whether Nature on the smallest of scales is random or absolutely predictable? In what follows, the second term is understood as causality, i.e., if an observer probes a system by exposing it to some impacts, reactions of the system coincide whenever impacts coincide up to a precision of measurement equipment; that is, *the same causes imply same effects*, so the behaviour of the system is completely predictable since a cause results in a unique effect within the measurement precision. The *randomness means that the “same” causes may result in different effects*. Specifically, causes whose numerical values are indistinguishable in measurement since the values coincide up to the precision of measurement equipment may result in effects which are distinguishable by measurement, i.e., differences of numerical values of respective effects exceed the measurement error. This is why we treat what follows randomness as chaos in a broad meaning since the definitive feature of chaos is its extreme sensitivity to negligible distortions/perturbations.

Recall that there are many nonequivalent mathematical notions of chaos; see, e.g., the expository paper [35]. One of the most common of these definitions is in the work of R. L. Devaney [36] [Definition 8.5] which reads as follows:

**Definition** **8** (Devaney’s chaos on metric spaces)**.** *Let F:X→X be a continuous function on a metric space X equipped with a metric d. The function F is said to be* chaotic *if it satisfies the following three conditions:*

(i) Sensitive dependence on initial conditions: *There is δ>0 such that, for any x∈X and any neighbourhood A⊂X of x, there exists y∈A and $n\in N_{0}$ such that $d(F^{n}\left(x\right),F^{n}\left(y\right))>\delta$.*
(ii) Topological transitivity: *Given any pair of open subsets U,V⊂X, there exists k∈N such that $F^{k}\left(U\right)\cap V\neq \emptyset$.*
(iii) Density of periodic points: *The set of all periodic points of F is dense in X (a point x∈X is called periodic if $F^{k}\left(x\right)=x$ for some k∈N).*

It is known that conditions (i)–(iii) are *not independent*. In [37], it is proven that sensitive dependence on the initial conditions is a redundant element in Devaney’s definition because it follows from topological transitivity and denseness of the periodic points; in [38], it is shown by construction of counter examples, that neither topological transitivity nor denseness of the periodic points follow from the remaining two properties. In [39], it is proven that chaos, according to Devaney’s definition, may exist in bounded but noncompact spaces without any nonperiodic orbits. For bounded metric spaces, however, the following theorem is true:

**Theorem** **6** (C. Knudsen, [39])**.** *Let F, X, d be as that in Definition 8; let X be bounded; let ${f=F|}_{Y}$ be a restriction of F to a dense subset Y of X. Then, we obtain the following:*

- *F:X→X is topologically transitive, if and only if f:Y→Y is topologically transitive;*
- *F:X→X exhibits sensitive dependence on the initial conditions, if and only if f:Y→Y exhibits sensitive dependence on the initial conditions.*

The following definition of chaos on a bounded metric space is from Knudsen.

**Definition** **9** (Knudsen’s chaos on bounded metric spaces [39])**.** *Let F be a continuous transformation of a bounded metric space X. If F has a dense orbit in X and if F exhibits sensitive dependence on the initial conditions, then F is said to be* chaotic.

We stress that to the best of our knowledge, *all definitions of chaos on metric spaces contain sensitive dependence on initial conditions as an inherent property*; other conditions vary, but the sensitive dependence condition is always present, [40]. For other various types of chaos on compact metric spaces X, see [35]. We only mention that a continuous map F:X→X is called *topologically chaotic* if topological entropy of *F* is positive. The topological chaos implies *Li-Yorke chaos*, which is yet one more widely known type of chaos, for whose definition the reader is referred to [35]. In addition, positive topological entropy implies *distributional chaos* of type DC2, [41]. Chaos can also be defined in terms of measure-preserving transformations of measure spaces rather than of metric spaces; see [41].

The “chaos-like” behaviour may also be expressed in terms of “blending capability” which we first illustrate by an example taken from [42]. If in a cocktail shaker of volume 1 there are 10 shares of gin and 90 shares of vermouth then, after *ergodic* shaking, in every volume *V* of the shaker there will be 10 shares of gin and 90 shares of vermouth *on average*, whereas after *strong-mixing* shaking, in every *V* there will be approximately 10 shares of gin and 90 shares of vermouth; after *weak-mixing* shaking, *in every V with the exception of some rare instants* there will be 10 shares of gin and 90 shares of vermouth. Formally, a measure-preserving transformation *F* is by definition strong mixing if $\lim_{n\to \infty }\mu \left(F^{-n}\left(A\right)\cap B\right))=\mu \left(A\right)\mu \left(B\right)$ for every μ-measurable subsets A,B. Thus, if μ is a probability measure, the strong-mixing transformation, after being applied a sufficiently large number of times, makes any two “events” A,B “independent” in the probabilistic meaning. As mentioned in Section 3.4, a 1-Lipschitz measure-preserving map can be neither strong nor weak mixing; only the ergodicity is possible.

Finalising the considerations of chaos, we claim that *1-Lipschitz functions $F:Z_{p}^{n}\to Z_{p}^{n}$ are deterministic and nonchaotic with respect to chaos of any type*. Indeed, due to the 1-Lipschizness, these functions exhibit no sensitive dependence on initial conditions, and their topological entropy is zero; hence, any metric entropy is zero; cf. Section 3.4. Moreover, as measure-theoretical chaos is defined only for measure-preserving maps, and as a 1-Lipschitz map $F:Z_{p}^{n}\to Z_{p}^{n}$ preserves the Haar probability measure if and only if it is an isometry, it can be easily shown that *F* is chaotic with respect to no type of measure-theoretic chaos defined in [41]. One may say, therefore, that totally consistent functions (see Definition 6) are the best candidates to be called *superdeterministic*. The latter term also must not be treated in the meaning which is common for physical theories [5] but rather as a mathematical notion to stress the “extremely nonchaotic” behaviour of the functions.

On the other hand, one may also say that totally consistent functions are similar to *Ianus Bifrons*: being deterministic with respect to a *p*-adic metric for every *p*, *the totally consistent functions can nevertheless be chaotic if considered as real functions on a real interval*. Let us consider an illustrative example.

A well-known “canonical” example of real chaotic maps, the logistic map L(x)=2x(1−x), maps a real closed interval [0,1] to [0,1]. The map *L* has positive entropy log2. On the other hand, *L* is a polynomial with integer coefficients; hence, it is a totally consistent function, thus its entropy as a *p*-adic 1-Lipschitz map z↦2z(1−z) (both topological and metric with respect to Haar probability measure) is 0, and $L:Z_{p}\to Z_{p}$ is not sensitive to initial conditions. The map *L* on $Z_{2}$ is not measure-preserving with respect to the Haar probability measure on $Z_{2}$; it has the only point of attraction (namely, 0) to which all orbits converge; thus, *L* is not topologically transitive on $Z_{2}$.

However, the map *L* is *ergodic on the 3-adic sphere $S_{1/27}\left(0\right)$* of radius 1/27 centred at 0 since 0 is a fixed point of *L* and $L^{\prime }\left(0\right)=2$ is a generator of the group of units modulo 9; see [23] [Theorem 4.79] or [43] [Theorem 5.7]. Specifically, the sphere $S_{1/27}\left(0\right)$ is a disjoint union of two 3-adic balls $B_{1/81}\left(27\right)$ and $B_{1/81}\left(54\right)$, the sphere is invariant under the action of *L* on $Z_{3}$, and the sphere is measurable with respect to the Haar probability measure on $Z_{3}$. Thus, the probability measure on $Z_{3}$ induces a probability measure on $S_{1/9}\left(0\right)$ with respect to which the action of *L* on the sphere is measure-preserving and ergodic. The set of all rational 3-adic numbers from $S_{1/27}\left(0\right)$ which lie in the real closed interval [0,1] is dense in [0,1] with respect to the real metric. Therefore, as 3-adic rational integers are indistinguishable from real numbers by measurement due to inevitable nonzero error, the map *L* can be judged as measure-preserving and ergodic.

Now consider the map *L* on the 3-adic sphere $S_{1/27}\left(1\right)$. The sphere is a disjoint union of balls $B_{1/81}\left(28\right)$ and $B_{1/81}\left(55\right)$. The sphere is invariant under the action of *L* on $Z_{3}$ and is measurable with respect to the probability measure on $Z_{3}$. The map *L* on $S_{1/27}\left(1\right)$ *is measure-preserving with respect to the induced probability measure but is not ergodic* by the criterion of ergodicity on *p*-adic spheres (see [23] [Theorem 4.79] or [43] [Theorem 5.7]) since $L^{\prime }\left(1\right)=-2$ is not a generator of the group of units modulo 9. The set $S_{1/27}\left(1\right)\cap \left[0,1\right]$ is dense in [0,1] with respect to the real metric; therefore, by the reasoning similar to that as above, the map *L* can be judged as measure-preserving but not ergodic.

Finally, *the map $L:Z_{p}\to Z_{p}$ is measure-preserving for no p* as *L* is not a bijective modulo *p*; cf. Theorem 2. However, the set $Z_{p}\cap Q\cap \left[0,1\right]$ is also dense both in $Z_{p}$ and in [0,1] with respect to the *p*-adic and to the real metrics accordingly, so the values of the map *L* that takes on $Z_{p}\cap Q\cap \left[0,1\right]$ define a unique map both on [0,1] and on $Z_{p}$ for every *p*. However, an observer’s measurement data may only be rational numbers due to the inevitable nonzero measurement error, and any rational number from [0,1] can be approximated with arbitrarily high accuracy (with respect to the real metric) by numbers from $Z_{p}\cap Q\cap \left[0,1\right]$ regardless of whichever *p* is taken. In other words, numbers from $Z_{p}\cap Q\cap \left[0,1\right]$ (as well as from $S_{1/27}\left(1\right)\cap \left[0,1\right]$, or from $S_{1/27}\left(0\right)\cap \left[0,1\right]$) are indistinguishable from numbers in Q∩[0,1] and from numbers in [0,1] by measurements due to nonzero measurement error, but the choice of metric (and of the dense subset) with respect to which the measured numbers are processed is crucial for the observer’s conclusion whether the obtained data are completely random or satisfy a strictly deterministic law.

All these facts can be judged as evidence in favour of the following assertion.

**Interpretation** **2** (Observer’s free choice of determinism/randomness)**.** *Due to the inevitable nonzero error in measurements of values of physical quantities, an observer’s conclusion as to whether Nature on the smallest of the scales is superdeterministic or random completely depends on the observer’s free choice of metric with respect to which the observer processes the measured numerical data*.

### 4.5. p-Consistent Functions

In view of the *finiteness assumption* (cf. the text which follows Definition 1), Conditions 1 may appear to be too restrictive since according to physical reasons, the number of “elementary causes” and “elementary effects” cannot be arbitrarily large; therefore, it does not exceed some *p*. This is a motivation to introduce the following class of causal functions, the (univariate) *p-consistent functions*: given a prime *p*, we denote via ${𝒞}_{p}\left(R\right)$ the class of all continuous (with respect to the usual metric on R) functions $\overset{˘}{f}:R\to R$ such that the following conditions are satisfied:

(i) $\overset{˘}{f}\left(Z_{p}\cap Q\right)\subset Z_{p}\cap Q$;
(ii) There exists a *p*-adic 1-Lipschitz function $f:Z_{p}\to Z_{p}$ such that the following are obtained:
  - $f\left(Z_{p}\cap Q\right)\subset Z_{p}\cap Q$
  - $f\left(z\right)=\overset{˘}{f}\left(z\right)$ for every $z\in Z_{p}\cap Q$

The multivariate *p*-consistent functions $R^{m}\to R^{n}$ can be defined similarly.

Loosely speaking, the functions from ${𝒞}_{p}\left(R\right)$ “are living simultaneously in two worlds”, the Archimedean one and the non-Archimedean one: any $\overset{˘}{f}\in {𝒞}_{p}\left(R\right)$ defines a unique 1-Lipschitz (i.e., automaton) function $f:Z_{p}\to Z_{p}$ since $Z_{p}\cap Q$ is dense in $Z_{p}$ with respect to *p*-adic metric, and vice versa, any *f* defines a unique continuous real function $\overset{˘}{f}:R\to R$ since $Z_{p}\cap Q$ is dense in R with respect to the real metric (this is why in what follows, we use the same symbol *f* for $\overset{˘}{f}$ as well).

The functions from ${𝒞}_{p}\left(R\right)$ may suit the best for physical modelling of causal dependencies both at the macro- and micro- scales since *values of $f\in {𝒞}_{p}\left(R\right)$ on, e.g., $N_{0}$, completely define the function f on R*.

From this definition, it immediately follows that *any function from ${𝒞}_{p}\left(R\right)$ can be represented via a Mahler series (6) where all $c_{i}$ are in $Z_{p}\cap Q$; the series converges both on R and on $Z_{p}$ with respect to the real and, accordingly, to the p-adic metric*. It would be interesting to find necessary and sufficient conditions on the coefficients $c_{i}$ when the series (6) defines a ${𝒞}_{p}\left(R\right)$-function. The general conditions are not yet known, but nevertheless it is clear that the class ${𝒞}_{p}\left(R\right)$ is rich; for instance, it contains not only polynomials over $Z_{p}\cap Q$ but also some rational functions.

**Example** **3.** *Given polynomials u,v∈Z[x] such that v(z)≢0(modp) for all $z\in Z_{p}$ and v(x)≠0 for all x∈R, the rational function f(x)=u(x)/v(x) is in $C_{p}\left(R\right)$. The rational functions f* are differentiable with respect to both the *p*-adic metric and the real metric; moreover, ${\overset{˘}{f}}^{\prime }=f^{\prime }$ everywhere on $Z_{p}\cap Q$ and $f^{\prime }\in {𝒞}_{p}\left(R\right)$*; c.f., [44] or [23] [Section 3.10.2].*

For k∈N, we denote as ${𝒞}_{p}^{k}\left(R\right)$ (respectively via ${𝒞}_{p}^{\infty }\left(R\right)$) the subclass of all functions which are *k*-times (respectively, infinitely many times) differentiable with respect to both *p*-adic and real metric, whose derivatives are also in ${𝒞}_{p}\left(R\right)$. Put ${𝒞}_{p}^{0}\left(R\right)={𝒞}_{p}\left(R\right)$. *It is natural to ask, therefore, whether there exist functions in ${𝒞}_{p}\left(R\right)$ which are not rational functions*. The answer is affirmative.

**Theorem** **7.** *There exist functions in ${𝒞}_{p}^{\infty }\left(R\right)$ which are not rational functions.*

**Proof.**  The theorem can be proven by employing ideas from [45,46]. The set $Z_{p}\cap Q$ is countable; let us enumerate its elements as $z_{1},z_{2},\ldots$. Define by simultaneous induction a sequence of functions $g_{0},g_{1},g_{2},\ldots$ and integers $m_{0}<m_{1}<m_{2}<\cdots$ as follows: put $g_{0}\left(x\right)=0$, $m_{0}=1$. For n≥1, consider the following polynomial over the ring $Z_{p}\cap Q$:

$$h_{n}\left(x\right)=\prod_{i=1}^{n}\left(r_{i}-x\right)=a_{n,0}+a_{n,1}x+\cdots +a_{n,n-1}x^{n-1}+{\left(-1\right)}^{n}x^{n}.$$

Put

$$g_{n}\left(x\right)=\frac{p^{n}h_{n}\left(x\right)}{\left(p^{2n}+1\right)\left\lceil \right|a_{n,0}\left|+\right|a_{n,1}|m_{n-1}+|a_{n,n-1}{\left|m_{n-1}^{n-1}+m_{n-1}^{n}\right\rceil }_{p}}$$

where |·| is the real absolute value and ${\left\lceil r\right\rceil }_{p}$ for r∈R is the smallest ℓ∈N such that r≤ℓ if ℓ≢0(modp), or ${\left\lceil r\right\rceil }_{p}=\ell +1$ if ℓ≡0(modp). Then, $g_{n}\left(x\right)$ is a polynomial over $Z_{p}\cap Q$ and $∥g_{n}\left(c\right)∥<p^{-n}$ for every c∈C whose complex absolute value $∥c∥\leq m_{n-1}$.

Now, if *n* is even, let $m_{n}$ be first integer larger than $m_{n-1}$ such that $\sum_{i=0}^{n}g_{i}\left(m_{n}\right)\geq 2$. If *n* is odd, let $m_{n}$ be the first integer larger than $m_{n-1}$ such that $\sum_{i=0}^{n}g_{i}\left(m_{n}\right)\leq -2$. Since the leading coefficient of $h_{n}\left(x\right)$ is ${\left(-1\right)}^{n}$, these conditions are always true if $m_{n}$ is large enough. After defining all $g_{n}\left(x\right)$, put $g\left(x\right)=\sum_{i=1}^{\infty }g_{i}\left(x\right)$. Then, the following is true:

- The sum $g\left(x\right)=\sum_{i=1}^{\infty }g_{i}\left(x\right)$ converges uniformly in the open complex disk $D_{m_{n}}\left(0\right)$ of radius $m_{n}$ centred at 0 for all n∈N because, except for the first *n* terms, every term $g_{i}\left(c\right)$ is bounded absolutely by $p^{-i}$, and the sum of these converges.
- Because the uniform convergence in an open subset of C preserves analyticity, the function *g* is analytic on every $D_{m_{n}}\left(0\right)$ and so also on the whole C.
- $g\left(Z_{p}\cap Q\right)\subset Z_{p}\cap Q$ since for every $z=z_{k}\in Z_{p}\cap Q$, all $g_{j}\left(z\right)=0$ for j≥k.
- In the sequence ${\left(g\left(m_{k}\right)\right)}_{k=1}^{\infty }$, the terms having odd indices *k* are less than −1, whereas the terms having even indices *k* are greater than 1 since $\sum_{i=0}^{n}g_{i}\left(m_{n}\right)\geq 2$ for even *n* and $\sum_{i=0}^{n}g_{i}\left(m_{n}\right)\leq -2$ for odd *n*, and the remaining terms in $g(m_{n})$ cannot change the whole sum for more than 1.

Thus, the function *g* is well-defined on the whole R; *g* is a continuous function with respect to the real metric, and according to the intermediate value theorem, the function *g* has a zero between $m_{n}$ and $m_{n+1}$ for all sufficiently large n∈N. Therefore, *g* has infinitely many zeroes in R and thus cannot be of the form u(x)/v(x), where u(x),v(x)∈Z[x]. The function *g* according to this construction is a complex analytic function which is analytic on the whole C; thus, the restriction of *g* on R is a function R→R which is infinitely many times differentiable everywhere in R, and each derivative is continuous with respect to real metric and thus is uniquely defined by its values on $Z_{p}\cap Q$, as $Z_{p}\cap Q$ is dense in R with respect to the real metric.

On the other hand, given $a,b\in Z_{p}\cap Q$, a≠b, there are unique k,n∈N such that $a=z_{k}$, $b=z_{n}$ with respect to the numeration of numbers in $Z_{p}\cap Q$. If n>k, then $g\left(a\right)=\sum_{i=0}^{n-1}g_{i}\left(a\right)$, $g\left(b\right)=\sum_{i=0}^{n-1}g_{i}\left(b\right)$; therefore, ${\left|g\left(a\right)-g\left(b\right)\right|}_{p}\leq {\left|a-b\right|}_{p}$ since $\sum_{i=0}^{n-1}g_{i}\left(x\right)$ is a polynomial over $Z_{p}\cap Q$; thus, a unique continuation of *g* to the whole $Z_{p}$ is a *p*-adic 1-Lipschitz function. Let ${\bar{g}}_{k}=g\;\mathrm{mod}\;p^{k}$ be a polynomial over $N_{0}$ obtained by the reduction modulo $p^{k}$ of the function *g* (it is clear that then $\deg{\bar{g}}_{k}\leq k$ via the construction of *g*). Then, the function $g:Z_{p}\to Z_{p}$ can be uniformly approximated by the polynomials ${\bar{g}}_{k}$ with respect to the *p*-adic sup-norm which is defined as follows: Given a *p*-adic 1-Lipschitz functions $u:Z_{p}\to Z_{p}$, the *p*-adic sup-norm is $\max\{|u\left(z\right){|}_{p}:z\in Z_{p}\}$. Therefore the function $g:Z_{p}\to Z_{p}$ is a *ℬ*-function, the Stone–Weierstrass completion of the polynomials over $N_{0}$ with respect to the said *p*-adic sup-norm; thus, *g* is infinitely many times differentiable with respect to the *p*-adic metric, all derivatives are *ℬ*-functions, and thus the derivatives are uniquely defined by their values on $Z_{p}\cap Q$, as $Z_{p}\cap Q$ is dense in $Z_{p}$ with respect to the *p*-adic metric; see [44] [Proposition 4.4.] or [23] [Section 3.10.2, Proposition 3.59].

Therefore, *g* is infinitely many times differentiable both on R and on $Z_{p}$, and the values of the derivatives both with respect to the real and to the *p*-adic metric coincide on $Z_{p}\cap Q$. This finally proves that *g* is a $C_{p}^{\infty }\left(R\right)$-function. □

The ${𝒞}_{p}\left(R\right)$-functions exhibit a sort of “hologram-likeness”. *The values a ${𝒞}_{p}\left(R\right)$-function takes on arbitrarily small real interval, completely define the function on R and on $Z_{p}$*. Recall that a complete hologram can be restored from a small piece of a holography plate.

**Theorem** **8** (Hologram-likeness of ${𝒞}_{p}\left(R\right)$-functions)**.** *Let $f,g\in {𝒞}_{p}\left(R\right)$ and let (α,β)⊂R be any open interval; then f=g if and only if f(x)=g(x) for all x∈(α,β)⊂R* (*equivalently, for all $x\in \left(\alpha ,\beta \right)\cap Z_{p}\cap Q$*).

**Proof.**  For n∈N, $d\in \{1,\ldots ,p^{n}-2\}$ put $z_{n,d}=\frac{d}{1-p^{n}}$; then, $z_{n,d}\in \left(-1,0\right)\subset R$, $d\in Z_{p}\cap Q$. If $d=\xi_{0}+\xi_{1}p+\cdots \xi_{n-1}p^{n-1}$ is the base-*p* expansion of *z* then

(10) $$z_{n,d}=\xi_{0}+\xi_{1}p+\cdots \xi_{n-1}p^{n-1}+\xi_{0}p^{n}+\xi_{1}p^{n+1}+\cdots \xi_{n-1}p^{2n-1}+\cdots$$

is a *p*-adic canonical form of $z_{n,d}$ as ${\left(1-p^{n}\right)}^{-1}=1+p^{n}+p^{2n}+p^{3n}+\cdots \in Z_{p}$; cf., Section 3.2. From 10, it immediately follows that the set *𝒵* of all these $z_{n,d}$ is dense in $Z_{p}$. Therefore, f=g on $Z_{p}$ if and only if f=g on *𝒵*, but f=g on $Z_{p}$ if and only if f=g on R.

Let $\alpha ,\beta \in Z_{p}\cap Q$, α<β. Put γ=−1 if α−β≤−1; let $\gamma =\frac{1}{1-p^{t}}$ be such that 0>γ>α−β if α−β>−1 for a suitable t∈N. It is clear from what we have already proven that f(x)=g(x) for all x∈R if and only if f(x)=g(x) for all $x\in (\gamma^{-1}\left(\beta -\alpha \right),0)$, as $(\gamma^{-1}\left(\beta -\alpha \right),0)⊃\left(-1,0\right)$. □

**Interpretation** **3** (“Causality” vs. “locality”)**.** *The proof of Theorem 8 shows that the “local” behaviour of ${𝒞}_{p}\left(R\right)$-functions completely defines their “global” behaviour. Given values of ${𝒞}_{p}\left(R\right)$-function takes on an arbitrarily small neighbourhood of an arbitrary point, the values the function takes at all other points can be “restored uniquely”. If the points of R are treated as “positions” and values of the function as “measurement data” of a physical system to which the function is ascribed, then* the data an observer obtains by probing a system in a given position let him completely predict values of physical quantities obtained by measurements at all other positions.

This property of ${𝒞}_{p}\left(R\right)$-functions is especially important since various classes of real functions can be approximated by ${𝒞}_{p}\left(R\right)$-functions.

**Theorem** **9** (Approximations of real functions by ${𝒞}_{p}\left(R\right)$-functions).

(i) *Any continuous function g:[a,b]→R can be uniformly approximated on [a,b]⊂R by ${𝒞}_{p}^{\infty }\left(R\right)$-functions.*
(ii) *Any continuous function g:[a,b]→R can be uniformly approximated on [a,b]⊂R by ${𝒞}_{p}^{\infty }\left(R\right)$-functions which are automaton functions of time-reversible automata.*
(iii) *Any continuous function g:[a,b]→R can be uniformly approximated by ergodic automata functions from ${𝒞}_{p}^{\infty }\left(R\right)$.*
(iv) *Any continuous function g:R→R that vanishes at infinity can be uniformly approximated on R by ${𝒞}_{p}^{\infty }\left(R\right)$-functions. *(recall that a continuous function g:R→R vanishes at infinity, if, for every ε>0, there exists a compact set K⊂R such that |g(x)|<ε for all x∈R\K).
(v) *Any continuous function g:R→R that vanishes at infinity can be uniformly approximated on R by ${𝒞}_{p}^{\infty }\left(R\right)$-functions which are automaton functions of time-reversible automata.*
(vi) *Any square-integrable function g:R→R *(and moreover, any function g:R→R that is integrable with its *n*-th power for some n∈N) *can be uniformly approximated by ${𝒞}_{p}^{\infty }\left(R\right)$-functions which are automaton functions of time-reversible automata.*

**Proof.**  The class ${𝒞}_{p}^{\infty }\left(R\right)$ contains all polynomial functions over Z. Chlodovsky theorem yields that a continuous real-valued function, which is defined on a real interval that does not contain an integer, can be uniformly approximated by polynomials over Z [33,34]. If the interval [a,b] contains integers, take $\alpha ,\beta \in Z_{p}\cap Q$ such that the interval $\left[a^{\prime },b^{\prime }\right]=\left[\alpha a+\beta ,\alpha b+\beta \right]$ contains no integers (e.g., take m∈N such that $|b-a|<p^{m}-1$ and put $\alpha =1/(p^{m}-1)$). Given a continuous function g:[a,b]→R, the function $g(\alpha^{-1}\left(x-\beta \right))$ can be uniformly approximated by polynomials $u_{i}\left(x\right)\in Z\left[x\right]$ on $\left[a^{\prime },b^{\prime }\right]$ by Chlodovsky’s theorem; thus, the function *g* can be uniformly approximated by polynomials $u_{i}\left(\alpha x+\beta \right))$ on [a,b]. However, ${\hat{u}}_{i}\left(x\right)=u_{i}\left(\alpha x+\beta \right)$ is a polynomial over $Z_{p}\cap Q$ in variable *x* since $\alpha ,\beta \in Z_{p}\cap Q$; thus, ${\hat{u}}_{i}\in C_{p}^{\infty }\left(R\right)$. This proves claim (i).

To prove claim (ii), consider the function $\tilde{g}\left(x\right)=\frac{g(x)-x}{p}$. In view of (i), since $\tilde{g}\left(x\right)$ is continuous on [a,b], $\tilde{g}$ can be uniformly approximated by ${𝒞}_{p}^{\infty }\left(R\right)$-functions $u_{i}$; thus, *g* can be uniformly approximated by functions $x+pu_{i}\left(x\right)$ which are also in ${𝒞}_{p}^{\infty }\left(R\right)$. However, given any 1-Lipschitz function $u:Z_{p}\to Z_{p}$, the function z+pu(z) is 1-Lipschitz measure-preserving according to Lemma 1. Thus, the ${𝒞}_{p}^{\infty }\left(R\right)$-functions $x+pu_{i}\left(x\right)$ are automata functions of time-reversible automata.

To prove claim (iii), note that the function *g* can be uniformly approximated by polynomials $w_{j}\left(x\right)$ over $Z_{p}\cap Q$; c.f., the proof of (i). Then, the difference equation $\frac{w_{j}\left(x\right)-x-1}{p}=\Delta {\tilde{w}}_{j}\left(x\right)$ has a solution ${\tilde{w}}_{j}\left(x\right)$ which is a polynomial since $\frac{w_{j}\left(x\right)-x-1}{p}$ is. These ${\tilde{w}}_{j}\left(x\right)$ can be uniformly approximated by polynomials $u_{ji}\left(x\right)$ over $Z_{p}\cap Q$; c.f., the proof of (i). Therefore, *g* can be uniformly approximated by polynomials over $Z_{p}\cap Q$ of the form 1+x+p·Δu(x) which are all ergodic according to Lemma 1.

To prove claim (iv), consider functions of the form $\frac{ru(x)}{1+pv{\left(x\right)}^{2}}$ where $r\in Z_{p}\cap Q$, u(x),v(x)∈Z[x], and degu(x)≤degv(x). All these functions vanish at infinity, are ${𝒞}_{p}^{\infty }\left(R\right)$-functions (c.f., Example 3) and separate points. Therefore, the R-algebra A generated by the set *A* of all these functions satisfies conditions of the Stone–Weierstrass theorem for locally compact spaces, i.e., the algebra is dense with respect to the topology of the uniform convergence in the Banach algebra of all real-valued continuous functions on R which vanish at infinity. However, the set *A* is dense in A.

In order to prove claim (v), note that in view of the proof of claim (iv), it suffices to approximate uniformly on R the functions of the form $h\left(x\right)=\frac{ru(x)}{1+pv{\left(x\right)}^{2}}$, where $r\in Z_{p}\cap Q$, u(x),v(x)∈Z[x], and 1≤degu(x)≤degv(x), by ${𝒞}_{p}^{\infty }\left(R\right)$-functions which are automaton functions of time-reversible automata. Represent $h\left(x\right)=\frac{x}{1+pv{\left(x\right)}^{2}}+\frac{p\tilde{u}\left(x\right)}{p(1+pv{\left(x\right)}^{2})}$, then $\tilde{u}\left(x\right)\in Z_{p}\left[x\right]$, $\deg\tilde{u}\left(x\right)\leq \deg v\left(x\right)$. Given $c_{i}\in Z_{p}\cap Q$, the function $\frac{x}{1+pv{\left(x\right)}^{2}}+c_{i}\cdot \frac{p\tilde{u}\left(x\right)}{1+pv{\left(x\right)}^{2}}$ vanishes at infinity; moreover, this function is a ${𝒞}_{p}^{\infty }\left(R\right)$-function, and it is a measure-preserving 1-Lipschitz function $Z_{p}\to Z_{p}$ since it is bijective modulo *p*, and its derivative modulo *p* vanishes nowhere; c.f., [44] [Corollary 3.3] or [23] [Theorem 4.45]. Taking a sequence ${\left(c_{i}\right)}_{i=0}^{\infty }$ over $Z_{p}\cap Q$ that converges to 1/p in R, we conclude that the function h(x) can be uniformly approximated on R by ${𝒞}_{p}^{\infty }\left(R\right)$-functions which are measure-preserving 1-Lipschitz functions $Z_{p}\to Z_{p}$; that is, automaton functions of time-reversible automata.

It is well known that functions which are integrable with their *n*-th powers, for some n∈N, can be uniformly approximated by Schwartz functions; but the latter are smooth and vanish at infinity. With (v), this proves claim (vi) and the theorem. □

**Example** **4.** *Wave function Ψ(x,t) vanishes at infinity since it must satisfy the condition $\lim_{t\to \pm \infty }\Psi \left(x,t\right)=0$; see, e.g., [47] [Section 1.4]. Thus, wave functions can be uniformly approximated by automaton functions of time-reversible automata.*

**Interpretation** **4** (Observer’s free choice of arrow of time)**.** *Due to the inevitable nonzero error in measurements of values of physical quantities, an observer’s conclusion on the direction of “arrow of time” completely depends on the observer’s free choice of metric with respect to which the observer processes the measured numerical data: according to claims (ii) and (v)–(vi) of Theorem 9, “causes” can be recovered from “effects”, with any desirable accuracy. The “entropic arrow of time” also depends on the choice of metric since the value of entropy does as well; c.f., Section 4.4*.

**Theorem** **10** (On finite automata ${𝒞}_{p}^{1}\left(R\right)$-functions)**.** *Let a finite automaton function $f\in {𝒞}_{p}^{1}\left(R\right)$; i.e., let f be differentiable both over R and over $Z_{p}$; let $f^{\prime }\in {𝒞}_{p}\left(R\right)$. Then, f is an affine function over $Z_{p}\cap Q$; i.e., f(x)=ax+b for suitable $a,b\in Z_{p}\cap Q$. Vice versa, all these affine functions are finite automaton functions from ${𝒞}_{p}^{\infty }\left(R\right)$.*

**Proof.**  Given a 1-Lipschitz function *f* for $n\in N_{0}$, $k\geq \lfloor \log_{p}n\rfloor +1$, consider functions $f_{n,k}:Z_{p}\to Z_{p}$ which are defined as follows:

(11) $$\begin{array}{c} f_{n,k}\left(z\right)=\frac{1}{p^{k}}\left(f\left(n+p^{k}z\right)-\left(f\left(n\right)\;\mathrm{mod}\;p^{k}\right)\right)=\\ \;\;\;\;\;\;\frac{f\left(n+p^{k}z\right)-f\left(n\right)}{p^{k}}-\frac{f\left(n\right)-f\left(n\right)\;\mathrm{mod}\;p^{k}}{p^{k}}=\frac{f\left(n+p^{k}z\right)-f\left(n\right)}{p^{k}}-f_{n,k}\left(0\right), \end{array}$$

for all $z\in Z_{p}$. The function *f* is an automaton function of a finite automaton if and only if the collection ℱ of function $f_{n,k}$ (where $n\in N_{0}$, k∈N={1,2,3,…}, $k\geq \lfloor \log_{p}n\rfloor +1$) contains only a finite number of pairwise distinct functions. Note that $f_{n,k}$ is the automaton function that corresponds to the automaton $A\left(s\left(n_{k}\right)\right)=\langle F_{p},𝒮,F_{p},S,O,s\left(n_{k}\right)\rangle$, where $s(n_{k})\in 𝒮$ is the state the automaton $A=A_{f}=\langle F_{p},S,F_{p},𝒮,O,s_{0}\rangle$ reaches after it has been fed by the input word $n_{k}$ (of length $p^{k}$) that corresponds to the base-*p* expansion of *n* (so the word $n_{k}$ may contain some leading zeros that correspond to higher order digits of the expansion). That is, there are N,K∈N such that for every $n\in N_{0},k\in N$, one finds $\overset{ˇ}{n}\leq N$, $\overset{ˇ}{k}\leq K$ such that $f_{n,k}\left(z\right)=f_{\overset{ˇ}{n},\overset{ˇ}{k}}\left(z\right)$ for all $z\in Z_{p}$.

Take $z_{k}=\frac{h}{p^{2k}-1}$ where h∈N. Note that $z_{k}\in Z_{p}\cap Q$ and that $\lim_{k\to \infty }p^{k}z_{k}=0$ both with respect to real metric and to the *p*-adic metric. Then,

(12) $$\frac{f_{n,k}\left(z_{k}\right)}{z_{k}}=\frac{f\left(n+p^{k}z_{k}\right)-f\left(n\right)}{p^{k}z_{k}}-\frac{f\left(n\right)-f\left(n\right)\;\mathrm{mod}\;p^{k}}{p^{k}z_{k}},$$

where $\lim_{k\to \infty }\frac{f\left(n+p^{k}z_{k}\right)-f\left(n\right)}{p^{k}z_{k}}=f^{\prime }\left(n\right)$ both with respect to the real metric and to the *p*-adic metric. Thus, from (11) it follows that $\lim_{k\to \infty }^{p}\left(f_{n,k}\left(u\right)-f_{n,k}\left(0\right)\right)=uf^{\prime }\left(n\right)$ for every $u\in Z_{p}$, $n\in N_{0}$. However, $f^{\prime }\left(n\right)$ is a derivative at $n\in N_{0}$ both with respect to the real metric and to the *p*-adic metric; however, $f^{\prime }\left(n\right)$ may take only a finite number of values due to the finiteness of the number of pairs n,k which enumerate pairwise distinct $f_{n,k}$. Therefore, $f^{\prime }\left(z\right)$ may take not more than a finite number of values on $Z_{p}$ since any $z\in Z_{p}$ is a *p*-adic limit of some sequence over $N_{0}$, and $f^{\prime }$ is a continuous function $Z_{p}\to Z_{p}$ according the conditions of the theorem. Hence, $f^{\prime }$ may take not more than a finite number of values on $Z_{p}\cap Q$ and thus on R since $Z_{p}\cap Q$ is dense in R and since $f^{\prime }$ is a continuous real function according the conditions of the theorem. Therefore, the derivative $f^{\prime }$ is a constant function over R and thus over $Z_{p}\cap Q$ and over $Z_{p}$; that is, f(x)=ax+b for some $a,b\in Z_{p}\cap Q$. Proposition 1 proves the converse claim of the theorem. □

**Note** **6.** *The theorem remains true for multivariate*  ${𝒞}_{p}\left(R\right)$*-maps* $F:Z_{p}^{m}\to Z_{p}^{n}$ *as well: affine maps over* $Z_{p}\cap Q$ *are the only maps which satisfy the multivariate version of Theorem 10. This can be proven by a similar argument, the details of which are omitted.*

**Interpretation** **5** (Finiteness implies linearity)**.** *This result may serve as a sort of hint as to why the mathematical formalism of quantum mechanics is the theory of linear operators over Hilbert space. As all “real-world” systems have a finite number of states, then when the duration of the temporal interval measured in the smallest (say, Planck) time units becomes comparable to the number of states, the finiteness reveals itself as the linearity.*

## 5. In the Middle of the Scales

In Section 1, we conjectured that if both “continuous” and “discrete” theories adequately describe physical reality at respective “ends of the scale”, the theories must “meet one another somewhere in the middle of the scale”. *In this Section, we argue that the “meeting point in the middle of the scale” is the wave function*. To do this, we first need to formalise the notion of an observer; actually, we will consider observers of two kinds, each for the respective ends of the scale.

### 5.1. Observation and Measurement at the Ends of the Scale

To begin with, let us introduce two types of observers, the *Big-endian* and the *Little-endian*. The names of the two observers are more related to big-end and little-end orders the bytes of representation of a number are read in computer science and less with *Gulliver’s Travels* by Jonathan Swift. Given a large non-negative number having a very long base-*p* expansion, the Big-endian is capable of observing only the highest order digits of the expansion, i.e., he knows the order of magnitude of the number and (up to a nonzero error) a mantissa since the Big-endian is not able to see the rightmost digits of the numbers. Conversely, the Little-endian sees the rightmost digits of the number, starting with the smallest order digit, but has no idea what are the leftmost digits and the order of magnitude of the number (although he assumes that the order is finite but very large). One may call the Big-endian a macro-observer and the Little-endian a micro-observer. However, both observers measure observable values which are rational *p*-adic integers. As already mentioned, real numbers are indistinguishable during measurements from rational *p*-adic integers $Z_{p}\cap Q$ due to the inevitable nonzero measurement error with respect to real metrics. This is why we assume that numerical values of observable are in $Z_{p}\cap Q$, and the Little-endian sees the first terms of the canonical *p*-adic expansion of the observable value, whereas Big-endian sees the highest order digits of the base-*p* expansion of the same value as of a real number. We explain this more formally.

It is known (see, e.g., [10]) that $z\in Z_{p}\cap Q$ if and only if *z* can be represented as $z=c+\frac{d}{p^{t}-1}$ for some t∈N and $d\in \{1,2,\ldots ,p^{t}-1\}$, c∈Z, or, if and only if the *p*-adic canonical representation of *z* is eventually periodic

(13) $$\begin{array}{c} z=\alpha_{0}+\alpha_{1}p+\cdots +\alpha_{r-1}p^{r-1}+\left(\beta_{0}+\beta_{1}p+\cdots +\beta_{t-1}p^{t-1}\right)p^{r}+\\ \;\;\;\;\;(\beta_{0}+\beta_{1}p+\cdots +\beta_{t-1}p^{t-1})p^{r+t}+\left(\beta_{0}+\beta_{1}p+\cdots +\beta_{t-1}p^{t-1}\right)p^{r+2t}+\cdots \end{array}$$

for suitable $\alpha_{j},\beta_{i}\in \left\{0,1,\ldots ,p-1\right\}$, $r\in N_{0}$, t∈N (the sum $\alpha_{0}+\alpha_{1}p+\cdots +\alpha_{r-1}p^{r-1}$ is absent in the above expression once r=0). In this case, the base-*p* representation of the fractional part of *z* as of a real number is as follows:

(14) $$z\mathrm{mod}1=0.{\left({\hat{\beta }}_{t-1-\bar{r}}{\hat{\beta }}_{t-2-\bar{r}}\ldots {\hat{\beta }}_{0}{\hat{\beta }}_{t-1}{\hat{\beta }}_{t-2}\ldots {\hat{\beta }}_{t-\bar{r}}\right)}^{\infty }\mathrm{mod}1,$$

where $\hat{\beta }=p-1-\beta$ for β∈{0,1,…,p−1}, and $\bar{r}$ is the least non-negative residue of *r* the modulo *t* if t>1 or $\bar{r}$ is zero otherwise.

To illustrate what Big-endian observations and Little-endian observations are, let r=1, t≫1, $\alpha_{0}=1$, $\beta_{t-2}=\beta_{t-1}=0$; thus, both “-endians” measure physical quantity *z* that takes values in [0,1]. Then, as none of the observers is able to measure the value *z* with a nonzero error, the Big-endian will obtain only the digits ${\hat{\beta }}_{t-2},{\hat{\beta }}_{t-3},\ldots ,{\hat{\beta }}_{t-n}$ for some n<t; meanwhile, the Little-endian will obtain $\beta_{0},\beta_{1},\ldots ,\beta_{m}$ for some m<t−1. Thus, the only information about *z* which possibly is common for both observers is the values $\beta_{t-\ell },\beta_{t-\ell -1},\ldots ,\beta_{k}$ for some k>0, ℓ>1. The two observers may communicate with each other and thus make only common guesses about what *z* is. Moreover, both do not know what *t* is; therefore, as t≫1, the only thing that both observers may know for sure is that 1≥z≥1−1/p. Note that *there are no “hidden variables” in this scenario* since both observers may unboundedly increase the precision of their measurement despite neither being able to measure quantities with a nonzero error.

### 5.2. p-adic Clocks

In this section, we introduce a *p*-adic model of the instrument which measure and indicates time, a *p-adic clock*; then, we prove that there exist only one clock, which is the same for all Little-endians and Big-endian, the *universal clock*.

**Figure 3:**
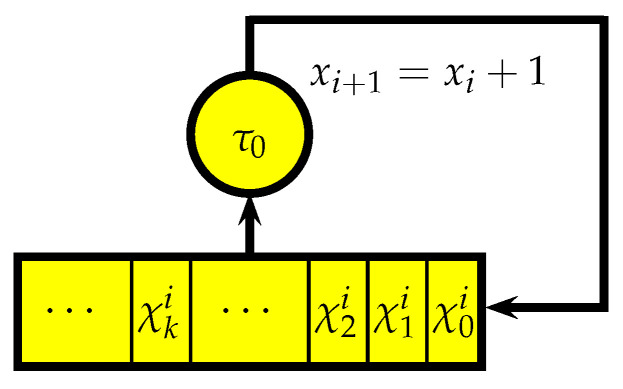
The *p*-adic clock.

A timekeeping element of the contemporary physical clock is a harmonic oscillator of a particular frequency, which is assumed to be a positive integer showing the number of periods per unit interval; therefore, the shortest time interval which can be measured is a reciprocal of the frequency. In order to measure the value of time elapsed, one merely counts the number of periods from one moment of time to another and represents this non-negative integer in some base, say, *p*, where *p* is the frequency of the oscillator. In what follows, we assume that *p* is a prime as to not overload the exposition with unimportant technical details. Thus, a model of such clock can be represented using the *p-adic odometer*, a dynamical system $f=\tau_{p}:z\mapsto z+1$ on the space of *p*-adic integers $Z_{p}$. If the initial point is $x_{0}\in Z_{p}$, e.g., $x_{0}=0$, put $f^{0}\left(x_{0}\right)=x_{0},f^{1}\left(x_{0}\right)=f\left(x_{0}\right)=x_{1},\ldots ,f^{i}\left(x_{0}\right)=f\left(f^{i-1}\left(x_{0}\right)\right)=x_{i}$, and then the base-*p* expansion of $x_{i}$ represents the time elapsed, $i=\sum_{j=0}^{\lfloor \log_{p}i\rfloor +1}\chi_{j}^{i}\cdot p^{j}$, where $\chi_{j}^{i}=\delta_{j}\left(i\right)\in \left\{0,1,\ldots ,p-1\right\}$ is the *j*-th digit of the base-*p* expansion of *i*. In loose terms, the *p*-adic clock is simply a counter whose face consists of windows; at each time moment *i*, each *j*-th window shows $\delta_{j}\left(i\right)$. It is convenient to assume that the number of windows is infinite to have the time elapsed be unrestricted; thus, we obtain the dynamical system $\tau_{p}$ on $Z_{p}$. Note that the initial state $x_{0}$ may be taken arbitrarily and not necessarily as $x_{0}=0$; then, to get the base-*p* representation of time elapsed since the initial moment, one has to perform subtraction $x_{i}-x_{0}$ in $Z_{p}$. The *p*-adic clock is depicted in Figure 3. To the right, the content of the registry is similar to a standard representation of time in decimal (rather than *p*-ary) fractions of a second (millisecond, microsecond, nanosecond, …) with Planck time at the rightmost position; meanwhile, to the left are decimal multiples of a second (petasecond, exasecond, …).

Speaking loosely, the registry in Figure 3 is like a face of a mechanical counter consisting of cogwheels. The period of the sequence of states of the rightmost cell of the registry (which can be judged as the rightmost cogwheel) is *p*, the period of the sequence of states of the second rightmost cell is $p^{2}$ since the figure in that cell changes once in a period of the rightmost cell, etc. The latter property is a definitive property of an ergodic transformation on $Z_{p}$; cf. Theorem 2. Therefore, all ergodic 1-Lipschitz transformations on $Z_{p}$ should be considered to be clocks, cf. (ii) of Theorem 3, as they can be "adjusted’" one to another since they all are conjugate to the *p*-adic odometer.

If the initial state of the odometer is taken to be 0 (i.e., each cell of the registry depicted by Figure 3 is 0), then after n∈N time units elapse, the registry will contain the base-*p* expansion of the number *n* since $\tau_{p}^{n}\left(0\right)=n$. Let us now take any ergodic 1-Lipschitz map $f:Z_{p}\to Z_{p}$, and any $t\in Z_{p}$ and any sequence ${\left(n_{i}\right)}_{i=0}^{\infty }$ over $N_{0}$ which converges *p*-adically to *t* (such a sequence exists as $N_{0}$ is dense in $Z_{p}$). It turns out then that for any $z\in Z_{p}$, the *p*-adic limit $\lim_{i\to \infty }^{p}f^{n_{i}}\left(z\right)$ exists; denote this limit via $f^{t}\left(z\right)$, then $\left(z;t\right)\mapsto f^{t}\left(z\right)$ is a 1-Lipschitz map $Z_{p}^{2}\to Z_{p}$ which is measure-preserving with respect to *t*; see [23] [Propositions 4.87–4.88, 4.90]. Therefore, *p-adic time t is well-defined*. For instance (see [23] [Example 4.89]), given an ergodic affine map f(z)=az+b on $Z_{p}$, the two-variate function $f^{t}\left(z\right)$ is of the form $f^{t}\left(z\right)=bt+z$ if a=1, and

$$f^{t}\left(z\right)=b\cdot \frac{a^{t}-1}{a-1}+a^{t}z,$$

if a≠1. Note that if the affine map z↦az+b is ergodic then b≢0(modp) and a≡1(modp) (see [23] [Theorem 4.36]); thus, both $a^{t}$ and $\frac{a^{t}-1}{a-1}$ are well-defined *p*-adic integers for every $t\in Z_{p}$.

The problem which immediately arises is that *p*-adic time *t* is well-defined for every $t\in Z_{p}$, but if *q* is a prime number distinct from *p*, the *p*-adic time *t* may be meaningless for a *q*-adic observer, the *q*-adic Little-endian, not to mention the Big-endian. Fortunately, however, *there is a clock (and therefore time) which is common both for all Little-endians and Big-endian. This clock/time is unique up to the direction of the time arrow*. It is clear that the clock, which is common for all *p*-adic Little-endians and Big-endian, must be a totally consistent function. The following theorem holds:

**Theorem** **11.** *Totally consistent functions which are measure-preserving for all prime p are exactly the functions x↦±x+c, where c∈Z; only the functions $\tau_{\pm }\left(x\right)=x\pm 1$ are ergodic for all prime p.*

This means that the “universal clock” is a standard odometer which runs forward ($\tau_{+}\left(x\right)=x+1$) or backward ($\tau_{-}\left(x\right)=x-1$).

**Proof** **of Theorem 11.** According to Theorem 5, any totally consistent function *g* is a polynomial; therefore, to be measure-preserving on $Z_{p}$, *g* must be (1) bijective modulo *p* and (2) its derivative $g^{\prime }\left(x\right)$ must vanish modulo *p* nowhere for all prime *p*; see, e.g., [23] [Theorem 4.45]. As *g* is 1-Lipschitz on $Z_{p}$ for all prime *p*, and $g^{\prime }\left(x\right)$ is a polynomial, the derivative exists and takes values from $Z_{p}$ for all prime *p*; hence, $g^{\prime }\left(Z\right)\subset Z$. Therefore, (as $g^{\prime }\left(x\right)$ is a polynomial), condition (2) implies that $g^{\prime }\left(Z\right)\in \left\{1,-1\right\}$, which means that $g^{\prime }$ is a constant, ±1. This means that *g* is the affine function, namely, either g(x)=−x+c or g(x)=x+c for some c∈Z (since g(0) must be an integer as g(Z)⊂Z due to total consistency). This proves the claim concerning measure-preservation.

The ergodicity claim follows from the ergodicity criterion for affine maps z↦az+b which implies that if the map is ergodic on $Z_{p}$ then a≡1(modp) and b≢0(modp); see [23] [Theorem 4.36]. As these conditions must hold for all prime *p*, we conclude that a=1 and b∈{1,−1}. □

**Interpretation** **6** (Free choice of temporal ordering at the smallest of scales)**.** *The only clock that is common for “both ends of the scale” is the standard odometer* $\tau \left(t\right)=t_{0}+t$ *which shows the time* $t-t_{0}\in R$ *elapsed since the moment* $t_{0}\in R$*. All observers acquire the value of the time elapsed up to a nonzero error with respect to the corresponding metrics. Therefore, in a contrast to a real observer (the Big-endian) the p-adic observers (the Little-endians) generally cannot determine with the “time stamps” of events which one of the two events happened earlier and which one later since there is no order on the field of p-adic numbers which agrees with field operations.*

**Note** **7.** *It is known that generally there is no ordering of events in quantum mechanics; see, e.g., [48].*

### 5.3. Digitalization

Initial automaton is a model of a (generally open) physical system prepared in some fixed state; the system is exposed by an experimenter to a time series of “elementary impacts” and thus produces the time series of “elementary reactions”. The impacts/reactions occurs at discrete instants of time since time is assumed to be discrete; for example, at Planck’s scale, the smallest time interval is Planck time $5.391247\left(60\right)\times 10^{-44}$ s. Concrete values of that smallest time interval depend on the process which is modelled (e.g.,. in smart contracts of digital economy the smallest interval is usually assumed to be 24 h) and are not specified; the definitive feature of the model is that “time flow” consists of “indivisible time intervals”.

The experimenter prepares a number of identical systems in the same state and probes them by exposing them to different impacts, observing reactions and thus obtaining a number of experimental points (〈impact〉; 〈reaction〉), where 〈★★★〉 are measured values of components of the impact–reaction pair. The experimenter then treats any measured value as a real number up to a nonzero real error.

In order to not overload the exposition, in what follows we consider a one-dimensional case mostly when the values 〈impact〉 and 〈reaction〉 are numbers rather than vectors. Up to normalisation, we may assume that the measured numerical values are all in the unit real interval [0,1]; thus, the experimenter obtains a number of experimental points in the real unit square $\left[0,1\right]\times \left[0,1\right]=I^{2}\subset R^{2}$. Namely, given an automaton A, let $f=f_{A}:Z_{p}\to Z_{p}$ be its automaton function (i.e., a 1-Lipschitz map). Consider a subset ℰ(f) of all the following points of the Euclidean unit square $I^{2}=\left[0,1\right]\times \left[0,1\right]\subset R^{2}$:

$$e_{k}^{f}\left(z\right)=\left(\frac{z\;\mathrm{mod}\;p^{k}}{p^{k}};\frac{f\left(z\right)\;\mathrm{mod}\;p^{k}}{p^{k}}\right)\in I^{2},$$

**Figure 4:**
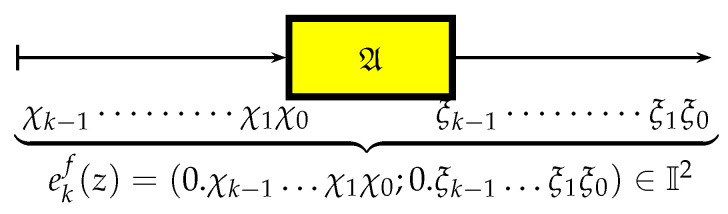
A point in the unit square $I^{2}\subset R^{2}$ produced by the automaton A.

$z\in Z_{p}$, k=1,2,…. Here, $z\;\mathrm{mod}\;p^{k}=\sum_{i=0}^{k-1}\chi_{i}p^{i}$ if $z\in Z_{p}$ is represented by its canonical form $z=\sum_{i=0}^{\infty }\chi_{i}p^{i}$, ($\chi_{i}\in F_{p};i=0,1,2,\ldots$). Note that $f\left(x\right)\;\mathrm{mod}\;p^{k}$ corresponds to a *k*-letter output word $\xi_{k-1}\cdots \cdots \cdots \xi_{1}\xi_{0}$ of the automaton which is fed by the *k*-letter input word $\chi_{k-1}\cdots \cdots \cdots \chi_{1}\chi_{0}$ which corresponds to $x\;\mathrm{mod}\;p^{k}$; cf. Figure 4.

Further, although all the word lengths *k* are finite, the clustering is equivalent to sending k→∞. Therefore, the clustering is equivalent to taking limit points of the closure 𝒫(f) of the set ℰ(f) with respect to the standard topology of $R^{2}$. We call 𝒫(f) a *plot* of *f*. Speaking very loosely, the plot is a picture the experimenter obtains as an output of the experiment which consists of a number of individual probes of a physical system which is prepared in the same state before each probe. Note that *the set of cluster points of the pictures for both experimenters, the Little-endian and the Big-endian, obtained as result of the experiment look very similar for the both since Little-endian makes the word lengths as long as possible to construct the cluster points while Big-endian is only capable of obtaining the points which correspond to sufficiently long words, i.e., the points which are close to the cluster points. This fact is crucial for the future construction of **wave function** by the both experimenters as well as for the **uncertainty relation** on which the both agree*.

Let us describe this procedure more formally. For $s=\sum_{j=-k}^{\infty }\zeta_{j}p^{j}\in Q_{p}$, ($\zeta_{j}\in \left\{0,1,\ldots ,p-1\right\},j\in Z$), let ${\left[s\right]}_{p}=\zeta_{0}+\zeta_{1}p+\zeta_{2}p^{2}+\cdots \in Z_{p}$ and ${\left\{s\right\}}_{p}=\zeta_{-k}p^{-k}+\cdots +\zeta_{-1}p^{-1}$ be the integral and fractional parts of *s*, respectively. Recall that any complex character of additive group $Q_{p}^{+}$ of the field $Q_{p}$ of *p*-adic numbers is of the form $\chi_{r}\left(s\right)=e^{2\pi i{\left\{sr\right\}}_{p}}$, where $r\in Q_{p}$; $\chi_{r}$ is a continuous group epimorphism into the group of complex roots of unity (which is isomorphic to the group $Q^{+}/Z^{+}$). Take r=1, denote $\chi_{1}$ via χ; given a 1-Lipschitz map $f:Z_{p}\to Z_{p}$, consider the mappings

$${\overset{ˇ}{f}}_{k}:e^{2\pi i{\left\{p^{-k}z\right\}}_{p}}\mapsto e^{2\pi i{\left\{p^{-k}f\left(z\right)\right\}}_{p}},\;\;\;\left(z\in Z_{p}\right),$$

for all $k\in N_{0}$. As every ${\overset{ˇ}{f}}_{k}$ maps points of the unit circle S into points of S, the pairs $\left(e^{2\pi i{\left\{p^{-k}z\right\}}_{p}};e^{2\pi i{\left\{p^{-k}f\left(z\right)\right\}}_{p}}\right)$ constitute a set of points on the unit torus $T^{2}=S\times S$. The unit square $I^{2}$ is a universal cover of the torus $T^{2}$; this way, the points $e_{k}^{f}\left(z\right)\in I^{2}$ are identified with the points $\left(e^{2\pi i{\left\{p^{-k}z\right\}}_{p}};e^{2\pi i{\left\{p^{-k}f\left(z\right)\right\}}_{p}}\right)\in T^{2}$, and in what follows, we do not differ between the point sets and speak either of the points on the surface of the torus $T^{2}$ or on the square $I^{2}$, whichever is more convenient.

**Definition** **10** (Plots of automata)**.** *Given an automaton A, let $f=f_{A}:Z_{p}\to Z_{p}$ be the automaton function. The closure 𝒫(f)=𝒫(A) of all the points $e_{k}^{f}=\left(\frac{z\;\mathrm{mod}\;p^{k}}{p^{k}};\frac{f\left(z\right)\;\mathrm{mod}\;p^{k}}{p^{k}}\right)$ in the square $I^{2}$ (or of all the points $\left(e^{2\pi i{\left\{p^{-k}z\right\}}_{p}};e^{2\pi i{\left\{p^{-k}f\left(z\right)\right\}}_{p}}\right)$ in the torus $T^{2}$), where k∈N, $z\in Z_{p}$ is called a (one-dimensional)* plot *of the automaton A or, similarly, of the automaton function $f=f_{A}$. The set ${𝒫}^{\prime }\left(f\right)={𝒫}^{\prime }\left(A\right)$ of all the limit points of the plot, the* derived set *of the set 𝒫(f)=𝒫(A), is called the* limit plot *of the automaton A (of the automaton function $f_{A}$).*

Recall that the *limit point*, *accumulation point*, or *cluster point* is a synonymic notion of the point such that every neighbourhood of which contains points other than that point. Recall also that the derived set of a closed set is also closed; thus, ${𝒫}^{\prime }\left(f\right)={𝒫}^{\prime }\left(A\right)$ is closed. Being closed, the set 𝒫(A) is measurable with respect to the Lebesgue measure on $R^{2}$; denote as α(A)=α(f) the measure of 𝒫(A). Respective notions for the general *n*-dimensional case, n>1, are defined as follows: for $z\in Z_{p}$, k,n∈N, n>1 denote

$$e_{k,n}^{f}\left(z\right)=\left(\frac{z\mathrm{mod}p^{k}}{p^{k}},\frac{f\left(z\right)\mathrm{mod}p^{k}}{p^{k}},\ldots ,\frac{f^{n-1}\left(z\right)\mathrm{mod}p^{k}}{p^{k}}\right)\in I^{n}\subset R^{n}.$$

The respective notation in this case is ${𝒫}_{n}\left(f\right)={𝒫}_{n}\left(A\right)$, ${𝒫}_{n}^{\prime }\left(f\right)={𝒫}_{n}^{\prime }\left(A\right)$, $\alpha_{n}\left(A\right)$, etc. We usually omit the index *n* when n=2.

**Theorem** **12** (The automata 0-1 law, [49])**.** *Given the arbitrary automaton A, the following alternative holds: either α(A)=0* (*equivalently, 𝒫(A) is nowhere dense in $I^{2}$*)*, or α(A)=1* (*equivalently, $𝒫\left(A\right)=I^{2}$*).

**Note** **8.** *Recall that nowhere dense sets can nevertheless have positive Lebesgue measures, for instance, the “fat” Cantor sets (e.g., the Smith-Volterra-Cantor set), which are also known as* ϵ*-Cantor sets; see e.g., [50]; however, this is not the case for the set* 𝒫(A)*. The Lebesgue measure of this set is 0 if and only if it is nowhere dense.*

Theorem 12 is true in the multidimensional case as well. We will say briefly that a 1-Lipschitz map $f:Z_{p}^{n}\to Z_{p}^{n}$ (or respective automaton whose automaton function is *f*) is *measure-0* in dimension *n* if $\alpha_{n}\left(f\right)=0$, and *measure-1* otherwise. It turns out that *all polynomials over Z whose degree is greater than 1 are measure-1 in all dimensions*. Actually, for f∈Z[x], a much stronger result is true: if degf≥2, then the distribution of points $e_{k,n}^{f}\left(z\right)$ in the unit hypercube $I^{n}$ tends to uniform as k→∞, for every n∈{2,3,4,…}. Specifically, the following theorem holds:

**Theorem** **13** ([11])**.** *Let f be a polynomial over Z, degf≥2. Then, the sequence ${\left(e_{k,n}^{f}\left(z\mathrm{mod}p^{k}\right)\right)}_{k=1}^{\infty }$ of random vectors weakly converges as k→∞ to a random vector having a continuous uniform distribution in ${\left[0,1\right)}^{n}$.*

Theorem 13 may be interpreted as showing another way by which chaos emerges.

**Interpretation** **7** (Emergence of chaos: The two ways)**.**

- ***1-st**: Chaos emerges from infinite “chaotic sequences” such as random real numbers by iterating them via Bernoulli-shift-like mappings, logistic mappings, etc; that is, when it is assumed a priori that “chaos does exist immanently”.*
- ***2-nd**: Chaos emerges from the “lack of knowledge what elementary causes happened at the very beginning”; that is, if a Big-endian observer is incapable of determining what the digits* $\xi_{0},\xi_{1},\ldots ,\xi_{k-1},\ldots$ *are of the input* $z=\sum_{j=0}^{\infty }\xi_{j}p^{j}\in Z_{p}$ *of the causal functionf* if *k* i*s small enough.*

Note that in the second case, the Little-endian observer *is* capable of determining the digits $\xi_{k}$ if *k* is “not too large”, so these digits are *not* hidden parameters. Nonetheless, further in the paper, we show that a specific uncertainty relation holds both for the Little-endian and Big-endian observers.

Note also that polynomials over Z whose degrees are greater than 1 are automaton functions of *infinite* automata; cf., Example 2. However, *any automaton function $f:Z_{p}\to Z_{p}$ of an infinite automaton can be uniformly approximated on $Z_{p}$ by automaton functions of finite automata*, for instance, by the functions $f_{n}:z\mapsto f\left(z\right)\mathrm{mod}p^{n}$. This fact, together with the finiteness assumption of Section 2, emphasises a distinguished role the finite automata play in further considerations; thus, we now pay special attention to finite automata.

**Theorem** **14** (see [23] [Section 11.1.2])**.** *Finite automata are measure-0 in all dimensions.*

**Example** **5.** *Automata may be infinite and measure-0; constants may be measure-1:*

- *The automaton whose automaton function is $f\left(z\right)=z+(z^{2}\mathrm{OR}\left(-\frac{1}{3}\right))$, ($z\in Z_{2}$), is infinite and measure-0. Here, OR is bit-by-bit logical ∨ with no carries to higher order bits; that is, if $z=\sum_{j=0}^{\infty }\zeta_{j}2^{j}$, then $z\mathrm{OR}\left(-\frac{1}{3}\right)=\sum_{j=0}^{\infty }\zeta_{2j}2^{2j}$ as $-\frac{1}{3}=\sum_{j=0}^{\infty }2^{2j}$ is a canonical 2-adic representation of $-\frac{1}{3}\in Z_{2}\cap Q$.*
- *The automaton whose automaton function is f(z)=C where C is a p-adic integer whose canonical representation corresponds to a Champernowne word is a measure-1 automaton. Recall that a Champernowne word is a word obtained via concatenation of the base-p expansions of numbers 1, 2, 3, 4, 5, 6, …; for instance, the 2-adic Champernowne word is 10111001101011….*

In short, Theorems 12 and 14 imply that plots of finite automata cannot contain “figures” but may contain “lines”. *These lines are of the utmost importance in further considerations since they may naturally be treated as “experimental curves” obtained by probing a physical system* both by Little-endian and Big-endian observers. It turns out that smooth lines from limit plots of finite automata are *windings of torus*; therefore, the lines may be treated as *sine waves*, so *the smooth lines in the limit plot of a finite automaton constitute a collection of sine waves*. Moreover, *the waves are limit plots of finite affine automata*. Now, we express these facts rigorously.

Recall that a *knot* is a smooth embedding of a circle S into $R^{3}$ and a *link* is a smooth embedding of several disjoint circles in $R^{3}$; cf. [51]. We will consider only special types of knots and links, namely, torus knots and torus links. Informally, a torus knot is a smooth closed curve without intersections which lies completely in the surface of a torus $T^{2}\subset R^{3}$, and a link (of torus knots) is a collection of (possibly knotted) torus knots; see, e.g., [52] [Section 26] for formal definitions.

We also need a notion of a winding of a torus. Formally, a winding of a torus is any geodesic on a torus. Recall that geodesics on torus $T^{2}$ are images of straight lines in $R^{2}$ under the mapping (x;y)↦(xmod1;ymod1) of $R^{2}$ onto $T^{2}=R^{2}/Z\times Z$; cf., e.g., [53] [Section 5.4].

**Definition** **11** (Winding of the torus)**.** *A* winding of the torus *is an image of a straight line in $R^{2}$ under the map mod1:(x;y)↦(xmod1;ymod1) of the Euclidean plane $R^{2}$ onto the 2-dimensional real torus $T^{2}=R^{2}/Z\times Z=S\times S\subset R^{3}$. If the line is defined by the equation y=ax+b, we say that a is a* slope *of the winding C(a,b). We denote via C(∞,b) a winding which corresponds to the line x=b, the* meridian *, and say that the slope is ∞ in this case. Windings C(0,b) of slope 0 (i.e., the ones that correspond to straight lines y=b) are called* parallels.

In dynamics, windings of torus $T^{2}$ are viewed as orbits of *linear flows on the torus*; that is, of dynamical systems on $T^{2}$ defined by a pair of differential equations of the form $\frac{dx}{dt}=\beta ;\frac{dy}{dt}=\alpha$ on $T^{2}$ and thus by a pair of parametric equations x=(βt+τ)mod1;y=(αt+σ)mod1 in Cartesian coordinates; cf., e.g., [54] [Section 4.2.3].

**Note** **9.** *It is well known that a winding defined by the straight line* y=ax+b *is dense in* $T^{2}$ *if and only if* −∞<a<+∞ *and the slope* $a=\frac{\alpha }{\beta }$ *is irrational; see, e.g., [54] [Proposition 4.2.8] or [53] [Section 5.4].*

**Figure 5:**
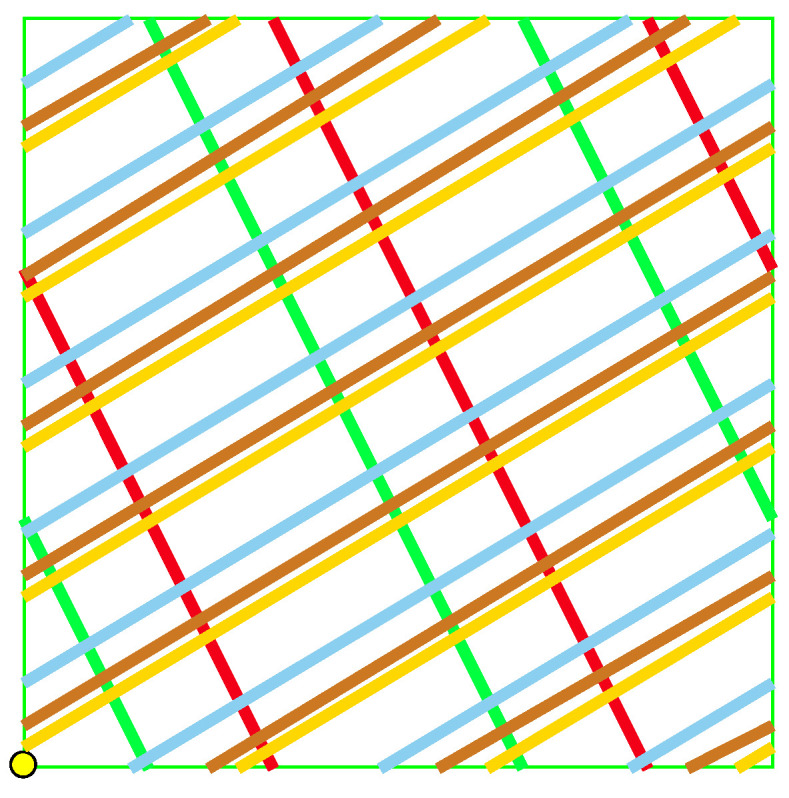
Limit plot in $R^{2}$ of an automaton having two affine subautomata.

**Figure 6:**
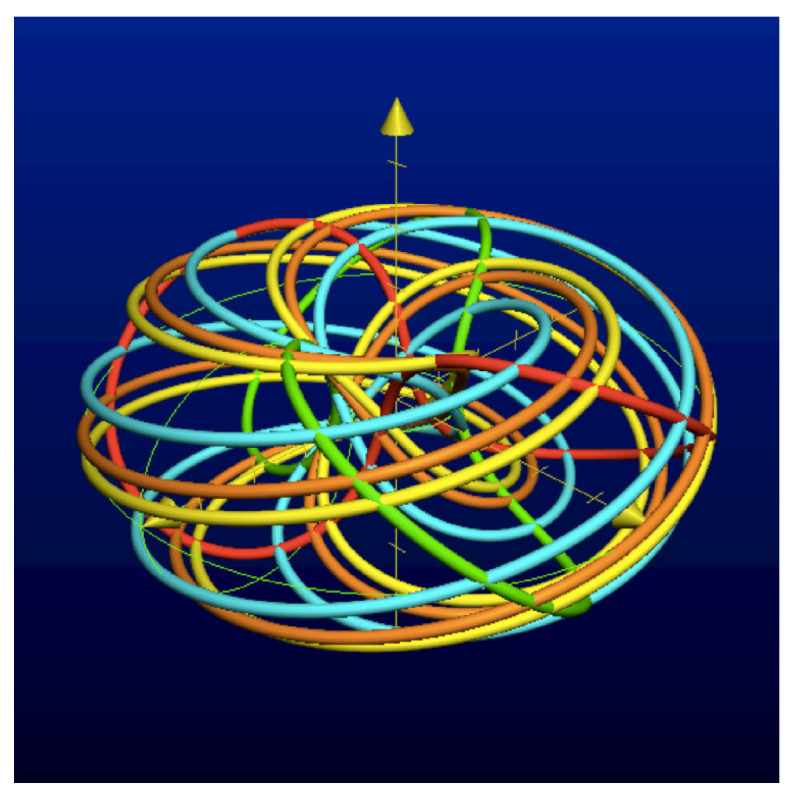
Limit plot of the same automaton on the torus $T^{2}$ in $R^{3}$.

Theorem 15 which follows states that $C^{2}$-smooth lines (i.e., those which are twice differentiable and have continuous second derivatives) in ${𝒫}^{\prime }\left(f_{A}\right)$ are windings of the torus $T^{2}$ provided the automaton A is finite; cf., Figure 5 and Figure 6.

**Theorem** **15** ([10])**.** *Let $f:Z_{p}\to Z_{p}$ be an automaton function of a finite automaton; let g be a $C^{2}$-function with domain [a,b]⊂[0,1)⊂R and range [0,1)⊂R. Let the graph G(g)={(x;g(x)):x∈[a,b]} of the function g lie completely in 𝒫(f). Then, there exist $a,b\in Q\cap Z_{p}$ such that g(x)=(ax+b)mod1 for all x∈[a,b]; moreover, there is a winding of the torus $T^{2}$ which lies completely in 𝒫(f) and which contains the graph G(g) of the function g. There are not more than a finite number of pairwise distinct windings of the unit torus $T^{2}$ in ${𝒫}^{2}\left(f\right)$; all of these are images of real affine functions x↦ax+b for $a,b\in Z_{p}\cap Q$ under the mapping $\;\mathrm{mod}\;1:R^{2}\to T^{2}$.*

**Note** **10.** *The* $C^{2}$*-smoothness condition can be relaxed:* $C^{1}$*-smoothness is sufficient to ensure the affinity; see [55].*

Although Theorem 15, after proper restatement, holds for *m*-variate 1-Lipschitz maps $f:Z_{p}^{m}\to Z_{p}^{m}$ as well, see [10], we restrict considerations in the rest part of the paper mostly by a univariate case for simplicity.

The torus link which is a limit plot of a finite automaton affine function f:z↦az+b on $Z_{p}$ is completely described by the following theorem:

**Theorem** **16** ([10])**.** *Given a finite automaton affine function f:z↦az+b on $Z_{p}$, *(i.e., such that $a,b\in Z_{p}\cap Q$)*, represent a,b as irreducible fractions: $a=\frac{\alpha }{\beta };b=\frac{\alpha^{\prime }}{\beta^{\prime }}$, where $\alpha ,\beta ,\alpha^{\prime },\beta^{\prime }\in Z$, $\beta ,\beta^{\prime }≢0\;\left(\mathrm{mod}\;p\right)$. Then, the limit plot ${𝒫}^{\prime }\left(f\right)$ on the torus $T^{2}$ is a torus link which consists of N torus windings whose slope is a, where $N={\mathrm{mult}}_{p}\frac{\beta^{\prime }}{d}$ is a multiplicative order of p modulo $\frac{\beta^{\prime }}{d}$, $d=\gcd(\beta ,\beta^{\prime })$ is the greatest common divisor of $\beta ,\beta^{\prime }$, and N=1 if $\frac{\beta^{\prime }}{d}=1$. Every torus winding is a graph of the complex-valued function ψ(ρ,k):R→C on the torus $T^{2}$ for a suitable $k=0,1,\ldots ,{\mathrm{mult}}_{p}\frac{\beta^{\prime }}{d}-1$, where $\psi \left(\rho ,k\right)=e^{i(\frac{\alpha }{\beta }\rho -2\pi p^{k}\frac{\alpha^{\prime }}{\beta^{\prime }})}$, (ρ∈R).*

In cylindrical coordinates, every torus winding x↦ax+b of a torus that is obtained by revolving around *Z*-axis of a circle that is coplanar with the axis and has radius *r* and a centre at the distance *R* from the origin can be represented by the following parametric equations

(15) $$\left[\begin{array}{c} r_{0}\\ \theta \\ z \end{array}\right]=\left[\begin{array}{c} R+r\cos\left(ax+b\right)\\ x\\ r\sin\left(ax+b\right) \end{array}\right],\;x\in R.$$

If $a\in Z_{p}\cap Q$, then *a* is irreducible fraction α/β where α,β∈Z and p∤β; then, corresponding winding winds β times around the *Z*-axis and |α| times around a circle in the interior of the torus, whereas the sign of α determines whether the rotation is clockwise or counter-clockwise. Hence, “physical meaning” that can be ascribed to the coefficient $a=\frac{\alpha }{\beta }$ of the affine map z↦az+b, ($z\in Z_{p}$), which is a finite automaton function of affine automaton if and only if $a,b\in Z_{p}\cap Q$, is *frequency* (or, as a *wavenumber*, under a proper choice of units). The choice of sign + or − depends only on what direction of rotation is assumed to be “positive” or “negative”; thus, *polarization* and *spin* can be ascribed to the sign of *a* in relevant models.

Theorem 16 in view of representation (15) implies that the limit plot of a finite automaton whose function is z↦az+b, (where $a,b\in Z_{p}\cap Q$, *z* runs over $Z_{p}$) is in one-to-one correspondence to a complex-valued function $\psi :R\times N_{0}\to C$:

(16) $$\psi \left(x,k\right)=e^{i(ax-2\pi p^{k}b)},\;\mathrm{where}\;x\in R,k\in N_{0}$$

It is worth noting that the function ψ(x,k) is well-defined for all k∈Z since *p* is the invertible modulo $\beta^{\prime }/d$ and thus $e^{-2\pi ip^{k}b}$ is well defined for every k∈Z; cf., Theorem 16.

**Figure 7:**
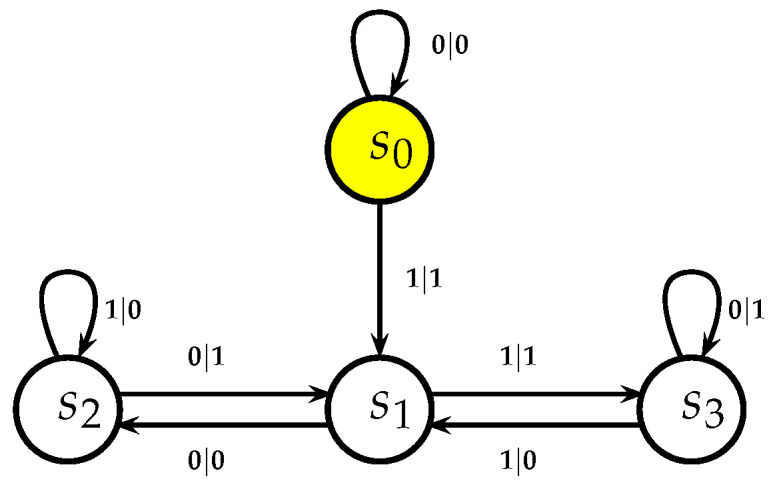
The automaton function is $z\mapsto -\frac{1}{3}z$; the minimal subautomaton function is $z\mapsto -\frac{1}{3}z-\frac{2}{3}$; ($z\in Z_{2}$), $s_{0}$ and $s_{1}$ are respective initial states.

**Figure 8:**
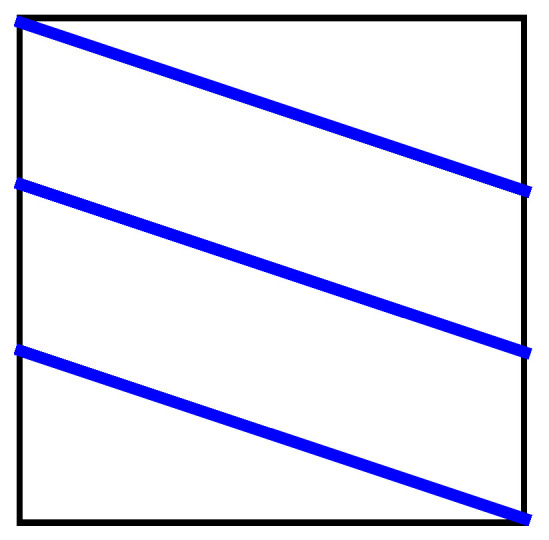
Limit plots of the automaton and of its minimal subautomaton coincide.

**Note** **11.** *According to Theorem 16, different affine functions* z↦az+b *may have identical limit plots. For instance, all the functions* f(z)=z+c *where* $c\in Z_{p}\cap Q$ *have identical limit plots which correspond to the function* $\psi \left(x\right)=e^{ix}$*. Note also that whenever a limit plot of a finite automaton* A *is the same as that of the finite automaton whose automaton functionfis affine,* f(z)=az+b, *there exist aminimal subautomatonof* A *(i.e., the one having no subautomata other than itself) which has exactly the same limit plot; see Figure 7 and Figure 8. A finite automaton is minimal if and only if its reduced state transition diagram is totally connected: Given two states* s,t∈𝒮*, there is finite word w such that when the automaton in state s accepts the word w, the automaton changes its state to t. If an automaton reaches a state which belongs to its (minimal) subautomaton, the automaton will never reach a state which does not belong to the subautomaton.*

**Figure 9:**
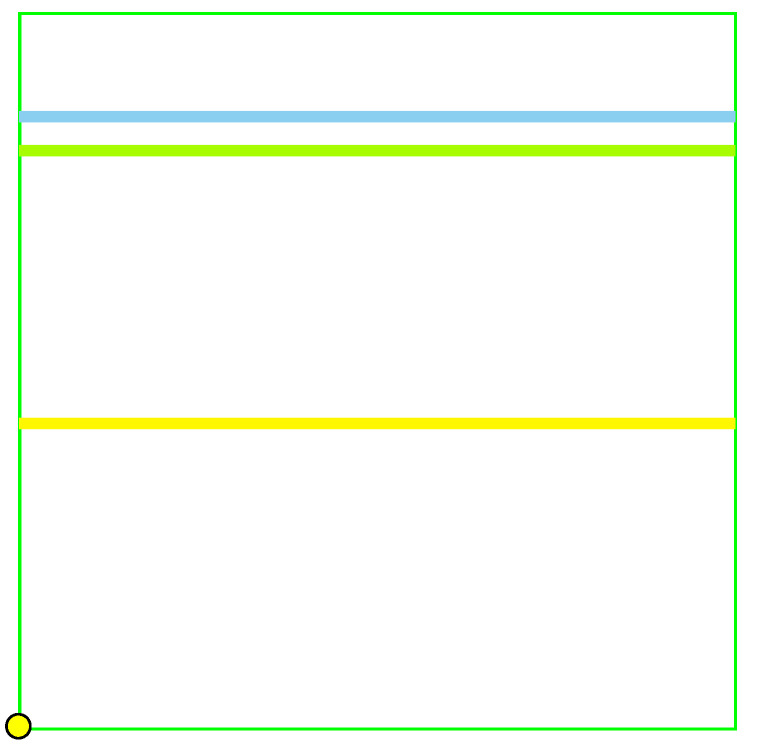
Limit plot of the function f(z)=2/7 ($z\in Z_{2}$), in $I^{2}$.

**Figure 10:**
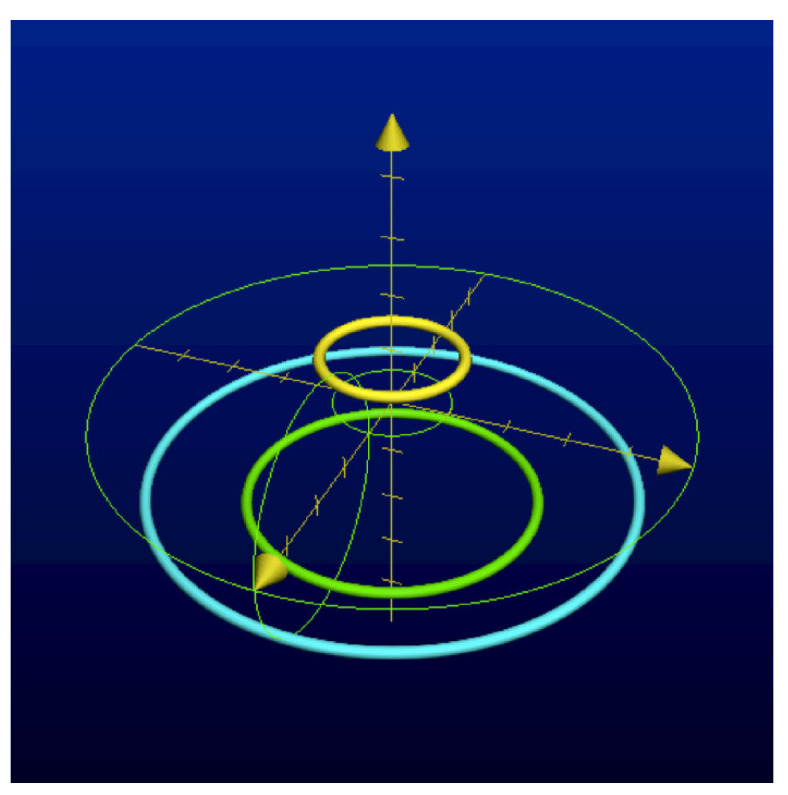
Limit plot of the same function on the torus $T^{2}$.

**Example** **6** (Limit plots of the automata)**.** *Figure 9 and Figure 10 show the limit plot of a constant function which is an automaton function of finite* autonomous *automaton; autonomous automata may be judged as models of either* isolated *or* closed *physical systems. Parallel lines shown by Figure 9 may be ascribed to energy levels.*

**Figure 11:**
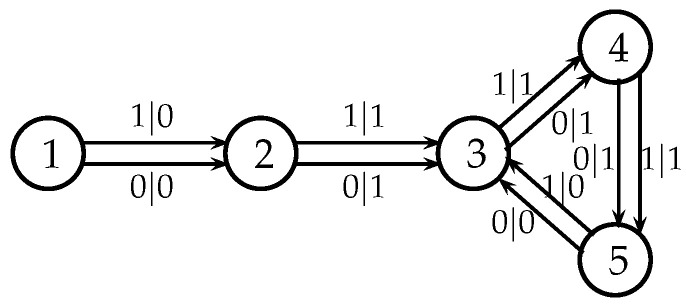
State transition diagram of the autonomous automaton whose automaton function $f:Z_{2}\to Z_{2}$ is a constant: f(z)=2/7, ($z\in Z_{2})$. State 1 is initial.

**Figure 12:**
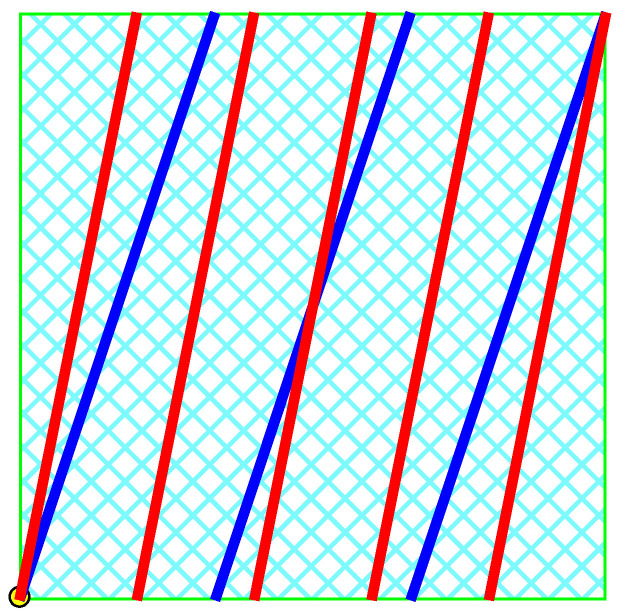
Limit plot of the automaton having two subautomata whose functions are z↦3z and z↦5z, ($z\in Z_{2}$).

**Figure 13:**
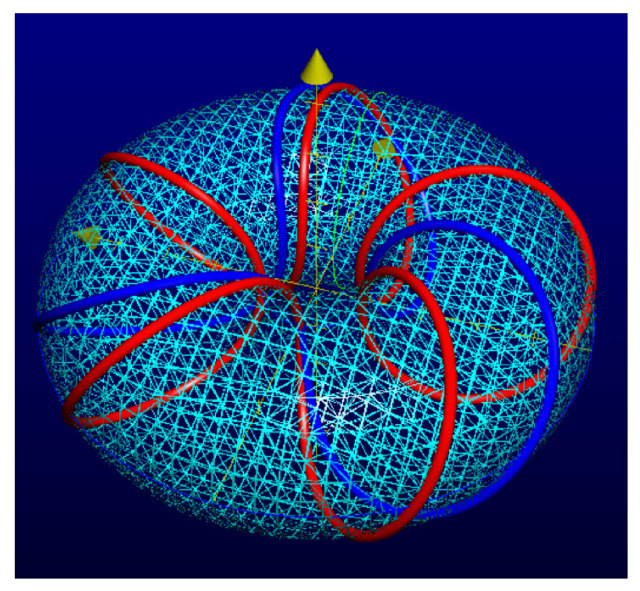
Limit plot of the same automaton on the torus $T^{2}\subset R^{3}$. The surface of the torus is made visible by cross-hatching.

**Figure 14:**
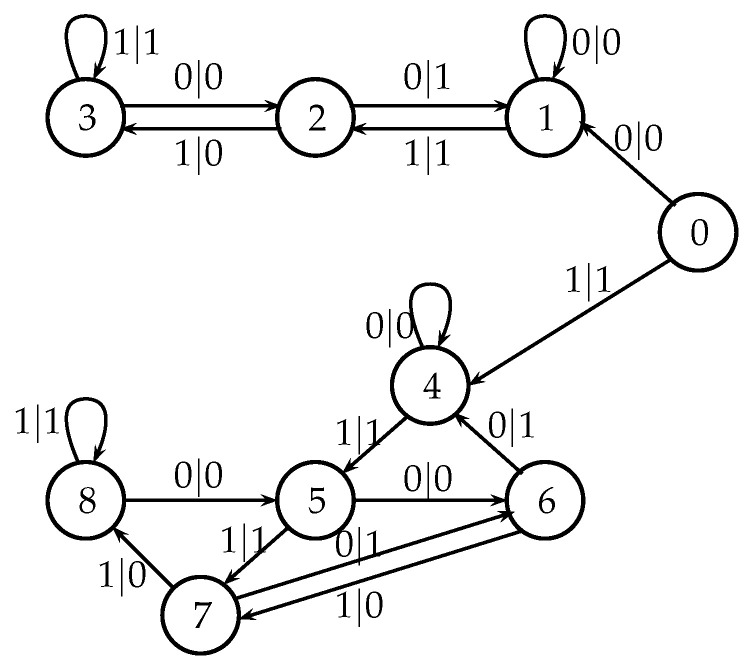
State transition diagram of the automaton having two minimal subautomata whose automata functions are z↦3z and z↦5z, $z\in Z_{2}$. The initial state is 0.

*The remaining examples are* nonautonomous *automata; these can serve as models of* open *physical systems. Figure 9 and Figure 10 depict limit plots produced of an autonomous automaton whose state transition diagram depicts Figure 11. Figure 12 and Figure 13 show the limit plot of an automaton having two minimal subautomata; the state transition diagram of the automaton is shown in Figure 14.*

**Figure 15:**
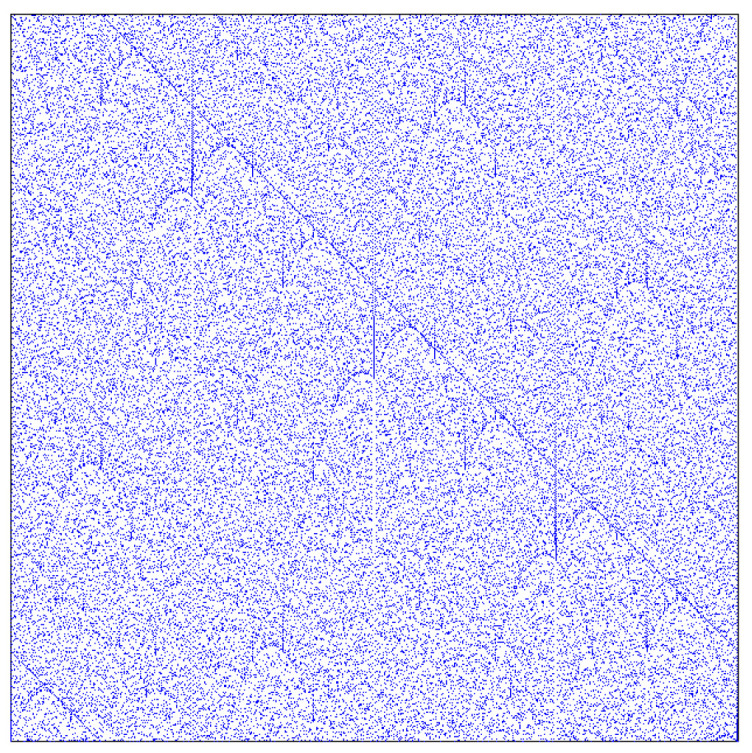
Plot of a finite automaton which is an approximation of a measure-1 automaton whose automaton function is $z\mapsto 1+3z+2z^{2}$, ($z\in Z_{2}$).

*Figure 15 represents a plot of a finite automaton which approximates a measure-1 (and thus infinite) automaton whose automaton function is $z\mapsto 1+3z+2z^{2}$, ($z\in Z_{2}$). Note the pronounced straight lines in the plot; these lines constitute the limit plot of a minimal subautomaton.*

**Figure 16:**
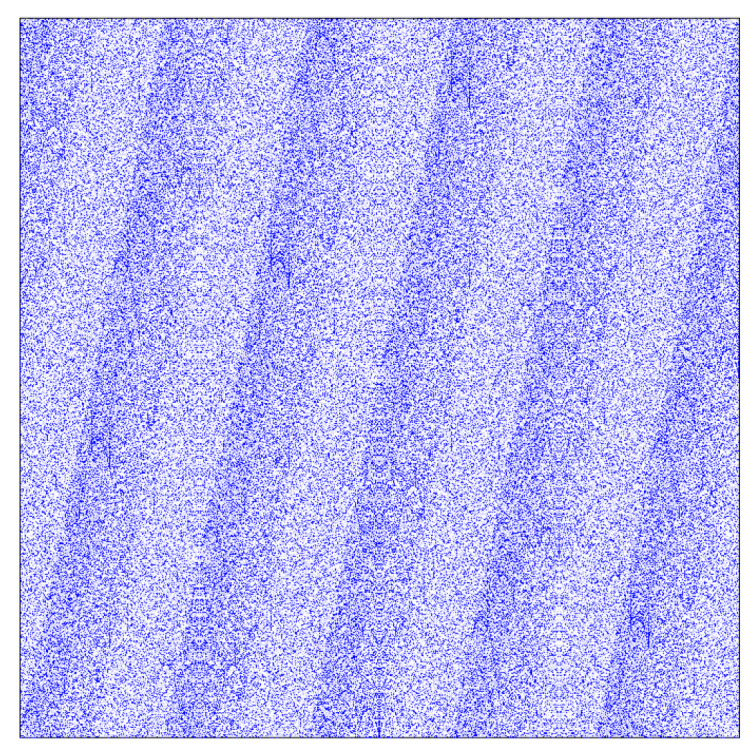
Plot of a measure-0 automaton having the only minimal subautomaton whose automaton function is z→5z, ($z\in Z_{2}$).

*Figure 16 depicts a plot of a measure-0 (but infinite) automaton which has the only minimal finite affine subautomaton; the automaton function of the latter subautomaton is z↦5z, ($z\in Z_{2}$). The limit plot of the latter automaton are red lines; cf., Figure 12; the state transition diagram is the lower part of the diagram shown in Figure 14.*

*Basically, the limit plot of a finite automaton whose minimal subautomata are affine consists of families of parallel straight lines in the unit square or, respectively, of links of the torus windings whose slopes are in $Z_{p}\cap Q$; cf., Figure 5, Figure 6, Figure 12, and Figure 13. The the minimal subautomata from the first example “exhibit nonzero phase shifts”, while for the ones from the second example, the “phase shifts” are 0. Both examples are automata having two minimal affine subautomata. The minimal subautomata from the first example (Figure 5 and Figure 6) have limit plots defined by the functions $f_{1}\left(z\right)=-2z+\frac{1}{3}$ (red and green windings) and $f_{2}\left(z\right)=\frac{3}{5}z+\frac{2}{7}$, (yellow, brown, and blue windings), respectively, $z\in Z_{2}$. The minimal subautomata from the second example (Figure 12 and Figure 13) have limit plots defined by the respective functions z↦3z (blue lines) and z↦5z (red lines), $z\in Z_{2}$.*

**Figure 17:**
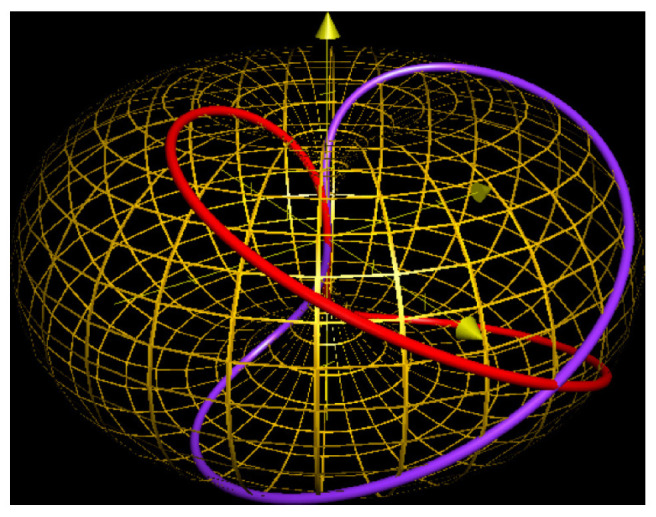
Limit plot of a finite automaton whose automaton function is z↦((zAND1)−((NOT(z))AND1))·z, ($z\in Z_{2}$) on the (horn) torus.

**Figure 18:**
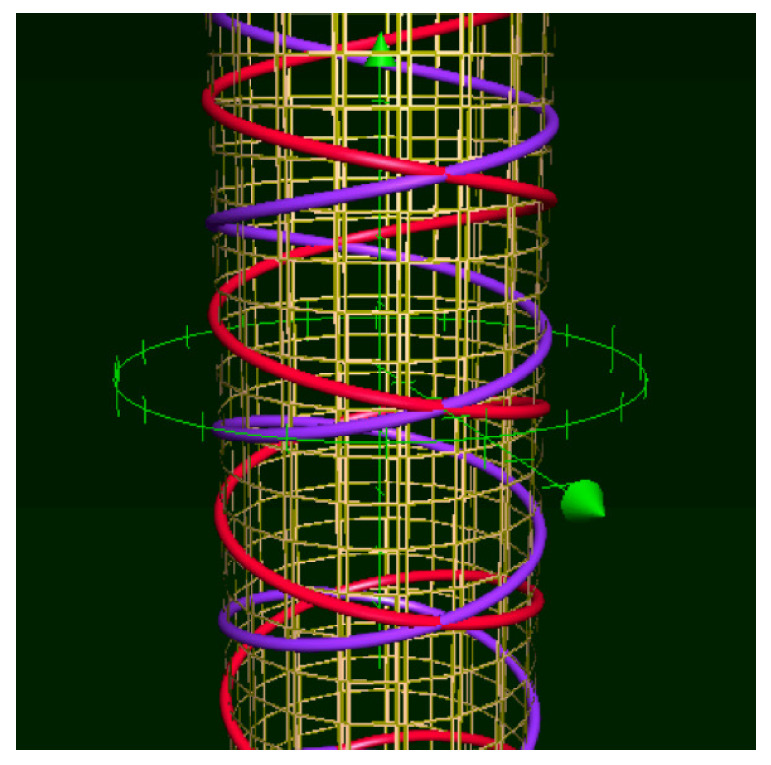
Solenoid that is a limit plot of the automaton having the same automaton function f(z)=((zAND1)−((NOT(z))AND1))·z, ($z\in Z_{2}$).

*The limit plot of a finite affine automaton whose automaton function is z↦az+b in the unit square $I^{2}$ consists of parallel straight lines with slope $a=\alpha /\beta \in Z_{p}\cap Q$; thus, the plot may be considered not only on the torus obtained by “gluing together” opposite sides of the square but also on a cylinder obtained by “gluing together” only a pair of opposite sides of the square. This way, one obtains* solenoid *rather than a torus link. This representation of a limit plot is also convenient in some cases. For instance, Figure 17 and Figure 18 depict the limit plot of the automaton whose automaton function is f(z)=((zAND1)−((NOT(z))AND1))·z, where AND and NOT are respectively bitwise logical “and” and bitwise logical “not” operations on base-2 expansions of numbers (with no carries), while “·” and “−” are usual multiplication and subtraction of numbers (with carries).*

**Figure 19:**
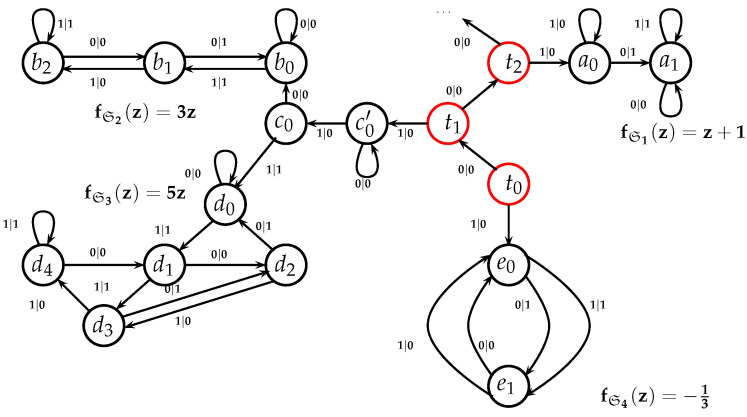
General automaton whose minimal subautomata are all finite and affine.

*Figure 19 represents the state transition diagram of a general automaton all whose minimal automata are finite and affine.*

### 5.4. Wave Functions Emerging from Automata

This section discusses the main notion of quantum theory, the wave function. Our goal is to derive wave functions from causal functions; that is, from automata. Functions (16) are building blocks of the construction of the wave function on the base of causal maps. To begin, we briefly outline the general idea of the construction.

**Figure 20:**
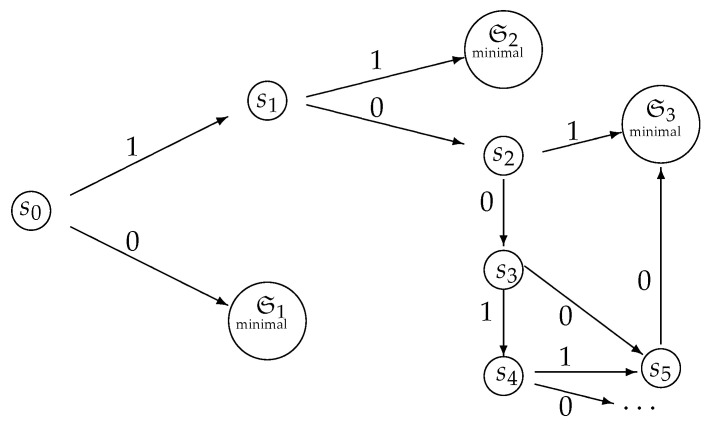
Example state transition diagram of 2-adic automaton having minimal subautomata (output symbols of labels of arrows are omitted). $s_{0}$ is the initial state. The respective probabilities of reaching subautomata $S_{1}$, $S_{2}$, and $S_{3}$ are 1/2, 1/4, and 11/64=1/8+1/32+1/64.

Recall that the reduced state transition diagram of a finite automaton is a digraph in which each path ultimately reaches a minimal subautomaton. There are no outgoing paths from subautomata. By feeding the automaton with random long words, to each minimal subautomaton we assign a probability for when the automaton reaches states which belong to the subautomaton; cf., Figure 20. Let automaton A be such that, being fed by random long words, the automaton at some finite step reaches, with a probability 1, a state which belongs to a minimal automaton which is finite and affine. The limit plot of every such subautomaton is described by a complex-valued function of the form (16).

To every minimal subautomaton that is finite and affine we ascribe its limit plot. There are only countably many such limit plots since there are only countably many such affine functions $Z_{p}\to Z_{p}$ that are automata functions of these subautomata: Due to the finiteness of the subautomata, coefficients of these affine functions must belong to the set $Z_{p}\cap Q$ which is countable. As every two minimal subautomata have no common states due to the minimality and as to every minimal subautomaton it is assigned a probability of reaching the subautomaton, to every limit plot one assigns a probability to “observe” that limit plot in the experiment, i.e., to obtain accumulation points in the unit square which constitute that limit plot. The probability is equal to a sum of all probabilities to reach the minimal subautomata having that plot. Therefore, these probabilities constitute a distribution assigned to the automaton; a *characteristic function* of that distribution is a (generally infinite) series whose terms are functions $\psi \left(x,k\right)=e^{i(ax-2\pi p^{k}b)}$ multiplied by values of respective probabilities; cf., (16) (there is a vast literature on characteristic functions of probability distributions; see, e.g., [56]). We argue that this characteristic function of the distribution may be treated as a wave function.

Proceeding to a formal rigorous construction, let us review a few preliminary conventions:

- We do not distinguish affine automata whose limit plots coincide, so the actual probability distribution related to the automaton is distribution of classes of finite affine subautomata having coinciding limit plots;
- We use terms “*p*-adic integer”, “infinite word over *p*-symbol alphabet”, and “infinite path in a state transition diagram” as synonyms; see Section 3.1, Section 3.2 and Section 3.3.

A word of caution: there is a one-to-one correspondence between all paths of length *k* in the state transition diagram and all numbers from $\left\{0,1,\ldots ,p^{k}-1\right\}$; however, to every number from $N_{0}=\left\{0,1,2,\ldots \right\}$, there corresponds an infinite number of paths: Every such path has a prefix which is simply a base-*p* expansion of a number and a suffix which consists of zeros only; cf., Section 3.1.

Given an automaton A, let S be its subautomaton. Let W(S) be the set of all infinite paths starting from the initial state of A in a state transition diagram of A which reach states of S at finite steps. Note that if a path *w* reaches S at *k*-th step, then all paths which correspond to infinite words having the same prefix of length *k* reach S at the *k*-th step; therefore, the *p*-adic integers which correspond to these paths constitute a *p*-adic ball of radius $p^{-k}$. Therefore, *all p-adic integers that correspond to infinite paths which reach the subautomaton S at finite steps constitute a disjoint union B(S) of balls of nonzero radii; hence, B(S) is a μ-measurable subset of $Z_{p}$ with respect to the Haar measure on $Z_{p}$ which is normalised so that $\mu (Z_{p})=1$. This way to S is assigned a probability μ(S)=μ(B(S))*.

Note that the set W(S) does not depend on a concrete state transition diagram of the automaton A, but to be more definite, one may assume that the state transition diagram of the automaton is reduced; thus, given an automaton function, the reduced state transition diagram of respective automaton is unique; cf., Section 3.3. In this case, some care should be taken speaking of paths since some arrows in the reduced state transition diagram may actually be loops; see, e.g., Figure 19. The paths (which we write from left to right) that begin at the initial state $t_{0}$ and have prefixes 0111, 01011, 010011, 0100011, … all reach the subautomaton $S_{3}$ on the fourth, fifth, sixth, seventh,.. steps respectively, so the probability to reach the subautomaton $S_{3}$ is 1/16+1/32+1/64+1/128+⋯=1/8 and $B(S_{3})$ is a disjoint union of balls $B_{1/16}\left(14\right)$, $B_{1/32}\left(26\right)$, $B_{1/64}\left(50\right)$, …, $B_{1/2^{k}}\left(2+3\cdot 2^{k-2}\right)$, … where k=4,5,6,….

Given two minimal subautomata S and T of the automaton A that are finite and affine, by virtue of the minimality one has B(S)∩B(T)=∅; thus, the probability that a random infinite path starting from the initial state reaches at a finite step some minimal subautomaton of the automaton A is the sum ∑μ(B(S)) taken over all minimal subautomata S which are finite and affine. We call an automaton A *ultimately affine* if the probability is 1. Note that if an ultimately affine automaton is infinite, then, according to König’s lemma (also known as Beth’s tree theorem) [57], there are infinite paths that never reach states belonging to these minimal subautomata. These paths constitute a μ-measurable subset in $Z_{p}$ but the measure of the subset is 0 since the subset is a complement to a countable union of balls whose measure is 1. For instance, the path 111… in the state transition diagram depicted by Figure 2 never reaches a minimal subautomaton (which has only one state, namely, $s_{1}$), but all other paths reach the subautomaton at finite steps, so the probability to reach that minimal subautomaton is 1.

**Definition** **12** (Plot equivalence of automata)**.** *Call the finite affine automata S and T* plot equivalent *$S\equiv_{P}T$ if their respective functions ψ:R×Z→C defined by (16) coincide; that is, if their limit plots coincide, $P^{\prime }\left(S\right)=P^{\prime }\left(T\right)$, i.e., if the limit plots are links of the same number of torus windings with a common slope.*

Given $a,b\in Z_{p}\cap Q$, denote via $S_{a,b}$ an automaton whose automaton function is z↦az+b. Let $\left[S_{a,b}\right]$ be the set of all minimal subautomata of A that are plot-equivalent to $S_{a,b}$. By virtue of the minimality, given $S,T\in [S_{a,b}]$, the subautomata S and T have no common states; therefore, B(S)∩B(T)=∅; that is, the probability

$$q_{\left[S_{a,b}\right]}=\sum_{S\in [S_{a,b}]}\mu \left(B\left(S\right)\right)$$

is well-defined. Given $a,b\in Z_{p}\cap Q$, the equivalence relation $\equiv_{P}$ induces an equivalence relation on the set of all pairs $\left(a;b\right)\in \left(Z_{p}\cap Q\right)\times \left(Z_{p}\cap Q\right)$ which we denote by the same symbol, i.e., $\left(a;b\right)\equiv_{P}\left(c;d\right)$ if and only if $S_{a,b}\equiv_{P}S_{c,d}$.

Let Spec(A) be the set of all equivalence classes defined by minimal subautomata of A which are finite and affine. Then, the series

(17) $$\Psi_{A}\left(\rho ,k\right)=\sum_{\left[S_{a,b}\right]\in \mathrm{Spec}\left(A\right)}q_{\left[S_{a,b}\right]}e^{i(a\rho -2\pi p^{k}b)}$$

converges absolutely for all ρ∈R, k∈Z and therefore defines a complex-valued function $\Psi_{A}\left(\rho ,k\right)$. Call the function $\Psi_{A}$ a *sharp wave function* assigned to the automaton A.

**Theorem** **17** (On automata having a prescribed wave function)**.** *Given non-negative real numbers $q_{1},q_{2},\ldots$ such that $\sum_{j=1}^{\infty }q_{j}=1$ and finite affine automata $S_{j}=S_{a_{j},b_{j}}$,*($a_{j},b_{j}\in Z_{p}\cap Q$, j=1,2,…)* which are pairwise plot-nonequivalent, there exists an ultimate affine automaton A such that $\mathrm{Spec}\left(A\right)=\{\left[S_{j}\right]:j=1,2,\ldots \}$, $q_{j}=q_{\left[S_{a_{j},b_{j}}\right]}$, and $\Psi_{A}\left(\rho ,k\right)=\sum_{j=1}^{\infty }q_{j}e^{i(a_{j}\rho -2\pi p^{k}b_{j})}$.*

To prove the theorem we require a lemma.

**Lemma** **2** (All discrete random variables can be modelled on $Z_{p}$)**.** *Given convergent series $\sum_{j=0}^{\infty }q_{j}=1$ of positive real numbers $q_{j}\in R_{\geq 0}$ there exist pairwise disjoint open sets $W_{j}\subset Z_{p}$ such that the normalised Haar measure μ of $W_{j}$ is $q_{j}$, j=0,1,2,….*

**Proof of** **Lemma 2.** Most likely, the lemma is known, but as the author is aware of no proper reference, a proof follows. Consider the *Monna map* $\mathrm{mon}\left(z\right)=\sum_{i=0}^{\infty }\alpha_{i}p^{-i-1}=0.\alpha_{0}\alpha_{1}\alpha_{2}\ldots \in \left[0,1\right]\subset R$ where $z=\sum_{i=0}^{\infty }\alpha_{i}p^{i}$ is a *p*-adic canonical expansion of $z\in Z_{p}$. Note that $\mathrm{mon}\left(B_{1/p^{k}}\left(a\right)\right)=\left[0.\alpha_{0}\alpha_{1}\ldots \alpha_{k-1},0.\alpha_{0}\alpha_{1}\ldots \alpha_{k-1}+p^{-k}\right]\subset \left[0,1\right]$, where $a=\sum_{i=0}^{k-1}\alpha_{i}p^{i}\in Z_{p}$; that is, the Monna map mon maps *p*-adic balls $B_{1/p^{k}}\left(a\right)\subset Z_{p}$ of radii $1/p^{k}$ centred at $a\in Z_{p}$ onto closed subintervals of length $1/p^{k}$ of the unit interval [0,1]; note that $\lambda \left(\mathrm{mon}\left(B_{1/p^{k}}\left(a\right)\right)\right)=\mu \left(B_{1/p^{k}}\left(a\right)\right)$ where μ is the Haar measure on $Z_{p}$ normalised so that $\mu (Z_{p})=1$, and λ is Lebesgue measure on the unit real interval [0,1], i.e., the length of the closed interval.

Split the unit interval [0,1] into pairwise disjoint open intervals $Q_{j}$ such that the length of the *j*-th interval $Q_{j}$ is $q_{j}$; namely, let $Q_{1}=\left(0,q_{1}\right)$, $Q_{2}=\left(q_{1},q_{1}+q_{2}\right)$, $Q_{3}=\left(q_{1}+q_{2},q_{1}+q_{2}+q_{3}\right)$, …; then, $Q={⋃}_{j=0}^{\infty }Q_{j}$ is λ-measurable and λ(Q)=1.

For each $Q_{j}$ let $B_{j}$ be a set of all balls of nonzero radii such that $\mathrm{mon}\left(B\right)\subset Q_{j}$ for every $B\in B_{j}$. As any two *p*-adic balls either disjoint or one is a subset of another one, the set $B_{j}$ is a countable disjoint union of balls of nonzero radii. Thus, $B_{j}$ is open as each *p*-adic ball of nonzero radius is clopen; hence, $B_{j}$ is μ-measurable. As every point from $Q_{j}$ lies in mon-image of some ball from $B_{j}$, we conclude that $\mu \left(B_{j}\right)=q_{j}$ and $\mu \left({⋃}_{j=1}^{\infty }B_{j}\right)=\sum_{j=1}^{\infty }\mu \left(B_{j}\right)=1$ as $B_{j}\cap B_{k}=\emptyset$ when j≠k by the construction. □

**Proof of** **Theorem 17.** This proof follows immediately from the proof of Lemma 2. Every $B_{j}$, j=1,2,… is a countable disjoint union of balls $B_{1/p^{r_{jm}}}\left(a_{jm}\right)$, m=1,2,3,…, centred at $a_{jm}=\sum_{k=0}^{r_{jm}-1}\alpha_{j,m,k}p^{k}\in Z_{p}$. Let branches of a *p*-adic tree be $\alpha_{j,m,0}\alpha_{j,m,1}\cdots \alpha_{j,m,r_{jm}-1}$, and let leafs be $B_{1/p^{r_{jm}}}\left(a_{jm}\right)$, j,m=1,2,…. In this digraph, replace all leafs $B_{1/p^{r_{jm}}}\left(a_{jm}\right)$ with state transition diagrams of automata $S_{m}\in \left[S_{j}\right]$. Thus, the constructed digraph is a state transition diagram of the automaton A which is the ultimate affine and such that $\Psi_{A}\left(\rho ,k\right)=\sum_{j=1}^{\infty }q_{j}e^{i(a_{j}\rho -2\pi p^{k}b_{j})}$. □

**Note** **12.** *From the proof of Theorem 17 it follows that the ultimate affine automaton may be either measure-0 or measure-1. The first case occurs when, for example, the series* $\sum_{j=0}^{\infty }q_{j}$ *is finite; therefore the automaton* A *is finite and thus measure-0. The measure-1 case occurs when, for example, all coefficients* $a_{j}\in Z_{p}\cap Q$ *constitute a dense subset in* R *and all* $b_{j}=0$.

In what follows, we will need a slightly generalised version of Lemma 2:

**Corollary** **1** (Generalized Lemma 2)**.** *Given convergent series $\sum_{j=0}^{\infty }q_{j}=q\leq 1$ of positive real numbers $q_{j}\in R_{\geq 0}$, there exist pairwise disjoint open sets $W_{j}\subset Z_{p}$ such that the normalized Haar measure μ of $W_{j}$ is $q_{j}$, j=0,1,2,….*

**Proof of** **Corollary 1.** Take [0,q] instead of [0,1] in the proof of Lemma 2 and modify the argument in an obvious way. □

Sharp wave functions may be considered as wave functions with respect to *discrete time* since the map $e^{2\pi ib}\mapsto e^{2\pi ip^{k}b}$ is equivalent to a *k*-digit *shift* of the base-*p* representation of *b* and a reduction modulo 1 of the resulting number. As *k* is the order of time elapsed (and is measured by *p*-adic clock see Section 5.2 and Figure 3) since the moment the automaton reaches a state from its minimal affine subautomaton whose automaton function is z↦az+b, a sharp wave function may be judged as the one the Little-endian can construct by observing reactions of a physical system at the smallest of scales.

We argue that a wave function with respect to *continuous time* can also be constructed by using ultimate affine automata. The core idea of the construct is using the beta representations of numbers rather than the base-*p* expansions. The beta representations of real numbers were first introduced by A. Rényi in 1957 and since then have attracted substantial attention in ergodic theory and symbolic dynamics; see, e.g., monograph [21].

Recall that given real β>1, a *β-representation* of real b≥0 is an infinite word $\chi_{0}\chi_{1}\cdots$ over the alphabet B={0,1…,⌊β⌋} such that $b=\sum_{j=-k}^{\infty }\chi_{k+j}\beta^{-k-j}$. Note that we consider β-representations of real b≥0 and not only of real b∈[0,1] as in [21]. Of course, in (17), we always may assume that b∈[0,1]; however, to assign real numbers to paths in state transition diagrams of automata we need beta representations of numbers from $N_{0}$ which then are converted into real numbers in a way similar to what we used in Section 5.3 by exploiting *p*-adic representations.

Specifically, we first use β instead of *p*. Thus, each arrow in a state transition diagram of the automaton whose input and output alphabets are ℬ, is labelled by a pair χ|ξ, where χ,ξ∈B; for an infinite path which starts from an initial state, there corresponds an infinite word $w=\chi_{0}\chi_{1}\cdots$ over alphabet ℬ; for *w*, we place a corresponding (⌊β⌋+1)-adic integer $\sum_{j=0}^{\infty }\chi_{j}{\left(\left\lfloor \beta \right\rfloor +1\right)}^{j}$. To construct a plot, we convert these (⌊β⌋+1)-adic integers into sequences of real numbers $\chi_{0}\beta^{-1}$, $\chi_{1}\beta^{-1}+\chi_{0}\beta^{-2}$, $\chi_{2}\beta^{-1}+\chi_{1}\beta^{-2}+\chi_{0}\beta^{-3}$, …, thus obtaining points $\left(\chi_{k-1}\beta^{-1}+\cdots +\chi_{0}\beta^{k-2};\xi_{k-1}\beta^{-1}+\cdots +\xi_{0}\beta^{k-2}\right)\in R^{2}$. To put it in other words, we simply use β-representations for input/output words of the automaton A when constructing a plot of the automaton, but the automaton function is still a 1-Lipschitz map from (⌊β⌋+1)-adic integers to (⌊β⌋+1)-adic integers. This way, we construct a sharp wave function $\Psi_{A}\left(\rho ,k\right)=\sum_{\left[S_{a,b}\right]\in \mathrm{Spec}\left(A\right)}q_{\left[S_{a,b}\right]}e^{i(a\rho -2\pi {\left(\left\lfloor \beta \right\rfloor +1\right)}^{k}b)}$ (cf., (17)), which is a well-defined complex valued-function of ρ∈R and k∈Z; then, we replace (⌊β⌋+1) by β in the formula, thus resulting in another complex-valued function of ρ∈R and k∈Z. The crucial point is that if 1<β≪2, i.e., if β=1+τ where 0<τ≪1, then $\beta^{k}={\left(1+\tau \right)}^{k}\approx 1+k\tau$. When τ is small (e.g., if $\tau =5.391247\left(60\right)\times 10^{-44}$ s, the Planck time) then for the Big-endian observer who is incapable of performing measurements with that accuracy (which is currently only about $10^{-20}$ s), kτ∈R is indistinguishable from continuous time. Thus, we obtain a *fuzzy wave function*

(18) $${\tilde{\Psi }}_{A}\left(\rho ,t\right)=\sum_{\left[S_{a,b}\right]\in \mathrm{Spec}\left(A\right)}q_{\left[S_{a,b}\right]}e^{i(a\rho -2\pi tb)}$$

which is ascribed to the automaton A. The function is well-defined for all ρ,t∈R since the series converges absolutely. From this point, the *sharp wave function (which is a discrete time function) can be viewed as an approximation of a fuzzy wave function (which is a continuous time function)*. Note that *since ⌊β⌋=⌊1+τ⌋=1, i.e., ℬ is a 2-letter alphabet, then necessarily p=2; see sharp wave function Formula (17)*.

The term “approximation” here is not rigorous (although some hint is already given by Example 4); to prove this statement with a full rigour is a separate problem which will be considered in the future. In the current paper, we only find an exact representation for β=1+τ under the *finiteness assumption* of Section 2, but before doing this, we illustrate the usage of that β-representation using the analogy of film which is discussed in Section 2. Each frame of a film contains a number of details, but to cause an illusion of motion to a viewer, only a small share of the whole number of details is changed from one frame to the next frame; the smaller the share is, the slower the motion appear to the a viewer. For a Little-endian viewer, the share is p−1 since he uses the base-*p* representation of numbers; in the case when the share is τ, one has the (1+τ)-representation. If 0<τ≪1, we have the case of a Big-endian viewer.

It is important to stress that *to represent numbers from $N_{0}$ in the base β, we use only non-negative powers of β in order to guarantee the uniqueness of β-representation for each number from $N_{0}$* since if negative powers of β=1+τ when τ≪1 are allowed in β-representations, then every number from $\left(0,\tau^{-1}\right)$ has a continuum of distinct β-representations provided $\tau <\frac{\sqrt{5}-1}{2}$ [58]. However, in such a case, the very problem of assigning a number to a finite path in a state transition diagram becomes ill-posed. Under said convention, the following theorem is true:

**Theorem** **18** (Finiteness assumption implies $\beta =\sqrt[{N}]{2}$)**.** *Let 1<β<2. If an automaton that performs the addition of β-representations of numbers from $N_{0}$ is finite then necessarily $\beta =\sqrt[{N}]{2}$ for some N∈N. For each N∈N, the addition of numbers from $N_{0}$ that are represented by $\sqrt[{N}]{2}$-representations can be performed with a finite automaton.*

**Proof.**  Number 1 admits the only β-representation $1=1+0\cdot \beta +0\cdot \beta^{2}+\cdots$ in non-negative powers of β as β>1. A finite automaton ultimately maps periodic sequences onto ultimately periodic sequences; therefore, if a finite automaton that maps pairs of infinite words into infinite words over the alphabet B={0,1} and performs 1+1=2, then necessarily

$$\begin{array}{ll} 2= & \alpha_{0}+\alpha_{1}\beta +\cdots +\alpha_{n-1}\beta^{n-1}+\\ & \;(\gamma_{0}+\gamma_{1}\beta +\cdots +\gamma_{s-1}\beta^{s-1})\beta^{n}+\left(\gamma_{0}+\gamma_{1}\beta +\cdots +\gamma_{s-1}\beta^{s-1}\right)\beta^{2n}+\cdots =\\ & \alpha_{0}+\alpha_{1}\beta +\cdots +\alpha_{n-1}\beta^{n-1}+\left(\gamma_{0}+\gamma_{1}\beta +\cdots +\gamma_{s-1}\beta^{s-1}\right)\beta^{n}\left(1+\beta^{n}+\beta^{2n}+\cdots \right), \end{array}$$

where $\alpha_{i},\gamma_{j}\in \left\{0,1\right\}$. As the series $1+\beta^{n}+\beta^{2n}+\cdots$ diverges, then all $\gamma_{j}=0$; hence,

(19) $$2=\alpha_{0}+\alpha_{1}\beta +\cdots +\alpha_{k-1}\beta^{k-1}+\beta^{k},$$

for suitable k≤n−1, $\alpha_{i}\in \left\{0,1\right\}$. If $\alpha_{0}=1$, then the right-hand side of (19) is not equal to the left-hand side; therefore, $\alpha_{0}=0$, and by substituting β=1+τ and collecting terms of positive degrees in τ we obtain the following (by binomial theorem):

$$2=\alpha_{1}\beta +\cdots +\alpha_{k-1}\beta^{k-1}+\beta^{k}=\left(\alpha_{1}+\cdots +\alpha_{k-1}+1\right)+\tau u\left(\tau \right),$$

where u(x) is a polynomial of variable *x* whose coefficients are in $N_{0}$. Hence, $1=\alpha_{1}+\cdots +\alpha_{k-1}+\tau u\left(\tau \right)$, where $\alpha_{j}\in \left\{0,1\right\}$, j=1,2,…,k−1.

If u(x) is a nonzero polynomial, then τu(τ)>0; thus, as $\alpha_{1}+\cdots +\alpha_{k-1}\in N_{0}$, we must conclude that $\alpha_{1}+\cdots +\alpha_{k-1}=0$: Otherwise, the right-hand side in $1=\alpha_{1}+\cdots +\alpha_{k-1}+\tau u\left(\tau \right)$ is strictly greater than is the left-hand side. Therefore, all $\alpha_{j}=0$ and thus $2=\beta^{k}$, i.e., $\beta =\sqrt[{k}]{2}$.

If u(x) is a zero polynomial, then necessarily $\alpha_{1}+\cdots +\alpha_{k-1}=1$. Therefore, there must be exactly one nonzero $\alpha_{j}$; hence, $2=\beta^{j}+\beta^{k}$, where 0<j<k. However, $2\neq \beta^{j}+\beta^{k}$ since β>1; so we get a contradiction.

The converse statement of the theorem is obvious since the addition of numbers represented by $\sqrt[{N}]{2}$-expansions is an “addition with carry to the *N*-th digit”; for example, when N=2 one has

$$\begin{array}{cc} + & \end{array}\begin{array}{ccccccccccccc} \ldots 1 & & 1 & & 1 & & 1 & & 1 & & 1 & & 1\\ \ldots 0 & & 0 & & 0 & & 0 & & 0 & & 0 & & 1\\ \ldots 0 & & 1 & & 0 & & 1 & & 0 & & 1 & & 0 \end{array}\begin{array}{cccccccccc} & = & & - & & \sqrt{2} & & - & & 1\\ & = & & 1 & & & & \\ & = & & - & & \sqrt{2} & & & \end{array}$$

□

It is worth warning the reader that Theorem 18 is *not* about the calculation of Planck time, whose value depends on the choice of units. In short, *Theorem 18 is about how much information one needs to have both worldviews, that of the Little-endian and the Big-endian, agree.* Specifically, Theorem 18 implies that the fuzzy wave function is the one which corresponds to an automaton over a $2^{N}$-symbol alphabet; that is, to the automaton whose function is $f:Z_{2}^{N}\to Z_{2}^{N}$, i.e., a *N*-variate 2-adic 1-Lipschitz map; see Section 3.3. Actually, *f* is a 1-Lipschitz map $Z_{2}\left(\sqrt[{N}]{2}\right)\to Z_{2}\left(\sqrt[{N}]{2}\right)$, where $Z_{2}\left(\sqrt[{N}]{2}\right)$ is the ring of integers of the field $Q_{2}\left(\sqrt[{N}]{2}\right)$; we leave further discussion of theory to future papers.

We remind the reader that for multivariate *p*-adic 1-Lipschitz maps, most theorems that have been proven or mentioned in this paper hold true; in particular, Theorem 15 holds true. Given a real function $G:H\to R^{n}$ whose domain is $H\subset R^{m}$, by the *graph of the function* (on the torus $T^{m+n}$), we mean the point subset $G_{H}\left(g\right)=\left\{\left(\vec{x}\mathrm{mod}1;G\left(\vec{x}\right)\mathrm{mod}1\right):\vec{x}\in H\right\}\subset T^{m+n}$. Note that if $\vec{y}=\left(y_{1};\ldots ;y_{k}\right)\in R^{k}$, then $\vec{y}\mathrm{mod}1$ stands for $\left(y_{1}\mathrm{mod}1;\ldots ;y_{k}\mathrm{mod}1\right)$.

**Theorem** **19** ([10])**.** *Let A be a finite automaton over the alphabet {0,1,…,p−1}, let A have m inputs and n outputs, and let $G=\left(G_{1};\ldots ;G_{n}\right):\left[\vec{a},\vec{b}\right]=\left[a_{1},b_{1}\right]\times \cdots \times \left[a_{m},b_{m}\right]\;{\left[0,1\right)}^{n}$* (*where $\left[a_{k},b_{k}\right]\subset \left[0,1\right)$, $G_{i}:\left[\vec{a},\vec{b}\right]\to \left[0,1\right)$, k=1,2,…,m*)* be a two-times differentiable function such that all its second partial derivatives are continuous on $\left[\vec{a},\vec{b}\right]$. If G(G) is a subset in a plot $P\left(A\right)\subset T^{m+n}$ of the automaton A, then there exist an m×n matrix $D=(d_{kj})$ and a vector $\vec{c}=\left(c_{1};\ldots ;c_{n}\right)$ such that $d_{kj}\in Q\cap Z_{p}$, $c_{j}\in Q\cap Z_{p}\cap \left[0,1\right)$* (*k=1,2,…,m; j=1,2,…,n*)* and $G\left(\vec{x}\right)=\left(\vec{x}D+\vec{c}\right)\mathrm{mod}1$ for all $\vec{x}\in \left[\vec{a},\vec{b}\right]$. There are not more than a finite number of D and $\vec{c}$ such that $d_{kj}\in Q\cap Z_{p}$, $c_{j}\in Q\cap Z_{p}\cap \left[0,1\right)$* (*k=1,2,…,m; j=1,2,…,n*) * and $G_{\left[\vec{a},\vec{b}\right]}\left(\left(\vec{x}D+\vec{c}\right)\mathrm{mod}1\right)\subset P\left(A\right)$ for some $\left[\vec{a},\vec{b}\right]\subset {\left[0,1\right)}^{m}$; moreover, if $G_{\left[\vec{a},\vec{b}\right]}\left(\vec{x}A+\vec{c}\right)\subset P\left(A\right)$ for some $\left[\vec{a},\vec{b}\right]\subset {\left[0,1\right)}^{m}$ then $G_{R^{m}}\left(\left(\vec{x}D+\vec{c}\right)\mathrm{mod}1\right)\subset P\left(A\right)\subset T^{n+m}$.*

The theorem implies that in the multivariate case, the sharp wave function is of the following form:

$$\Psi_{A}\left(\vec{x},r\right)=\sum_{\left[S_{A,\vec{b}}\right]\in \mathrm{Spec}\left(A\right)}q_{\left[S_{A,\vec{b}}\right]}e^{i(\vec{x}A-2\pi p^{r}\vec{b})};\;\left(\vec{x}\in R^{m};\vec{b}\in R^{n};r\in Z\right).$$

Therefore, *Theorem 18 implies that a univariate fuzzy wave function is actually a multivariate sharp wave function*; however, it is for a large number of dimensions. For instance, if $\sqrt[{N}]{2}=1+\tau$ where τ is of order of Planck time, then $N\approx \frac{\ln2}{\tau }\approx 10^{43}$; that is, the automaton function of respective automaton is a 1-Lipschitz map $Z_{2}^{10^{43}}\to Z_{2}^{10^{43}}$. This means that the matrices A in the above formula for the sharp wave function $\Psi_{A}\left(\vec{x},r\right)$ are $10^{43}\times 10^{43}$; that is, each of the matrices contains more entries than the number of atoms in the universe. An infinite-dimensional space is an adequate model for a $10^{43}$-dimensional space; this is why both the Big-endian and Little-endian would agree that wave functions “live” in Hilbert spaces. We postpone to a future paper more rigorous statements and proofs on how pure and fuzzy wave functions are related one to another; here, we only explain why both functions, which may be judged as “physical”, are elements of Hilbert space $\ell^{2}\left(\mathrm{Spec}\left(A\right)\right)$ of square-summable complex sequences whose terms are indexed by elements of the set Spec(A) (which is countable) since a “physical” wave function must be square-summable and the sum of squares of probability amplitudes must be 1. Recall that any separable Hilbert space is metrically isomorphic to $\ell^{2}$ and that the Fourier transform on the circle is such an isomorphism between the Hilbert space of square-integrable functions on [0,1]=I and the space $\ell^{2}\left(Z\right)$ of square-summable complex sequences whose terms are enumerated by integers. It is not difficult to construct sharp wave functions which can be judged as “physical” with this meaning. Indeed, take any sequence $q_{1},q_{2},\ldots$ of positive real numbers such that $\sum_{j=1}^{\infty }q_{j}=1$, and the series $\sum_{j=1}^{\infty }{\sqrt{q}}_{j}$ of positive square roots converges; by using Theorem 17, construct the automaton A. Then, function $\sum_{j=1}^{\infty }{\sqrt{q}}_{j}e^{i(a_{j}\rho -2\pi p^{k}b_{j})}$ is the one we are seeking.

We finalise the subsection with the following interpretation.

**Interpretation** **8** (Discrete spectrum; continuous spectrum)**.** *The measure-0 ultimate affine automata may be treated as models of physical systems having discrete (energy, frequency, …) spectra, while measure-1 ultimate affine automata may be treated as models of physical systems having continuous spectra.*

### 5.5. Uncertainty

In this subsection, we formally derive an uncertainty relation which holds for wave functions of automata. We stress, once again, that *despite the Litle-endian being capable of performing observation at the smallest scale and the Big-endian not being able to do so, the uncertainty relation, which can be treated as a time-energy uncertainty, holds for both observers, i.e., for Little-endian as well as for Big-endian; thus, no hidden parameters are assumed*.

The uncertainty relation we are going to deduce is an *entropic* one. A number of research papers have been devoted to discussing entropic uncertainty relations; see, e.g., the expository paper [59] and the references therein. The entropic uncertainty relation derived below is of a novel type since it relates the time during which a system reaches a “pure state" that can be ascribed to a minimal affine subautomaton and the state (i.e., an element of Spec(A)) itself. Note that as the Little-endian is capable of performing measurements at the smallest of scales, the time a system reaches a state that belongs to some minimal automaton is not 0, i.e., the “wave function collapse" is not momentary, it takes some minimal time intervals (e.g., some Planck time). Note that the collapse of wave functions as a finite-time process is discussed in the literature; see, e.g., [60].

To start with, we need to restate some results from Section 5.4 in terms of *prefix codes* since in what follows, we use some basic properties of the codes which may be found, e.g., in the book [61].

**Definition** **13** (Prefix code)**.** *A nonempty set C of finite nonempty words over a finite alphabet 𝒜 that consists of p>1 letters is called a* prefix code *if each word from C is a prefix of no other word from C.*

Let words from the nonempty set G of finite nonempty words over 𝒜 be ordered with respect to a nondecreasing order of their lengths, and let $\ell_{i}$ be the length of the *i*-th word (so $\ell_{1}\leq \ell_{2}\leq \cdots$). *The set G is a prefix code if and only if the following Kraft–McMillan inequality holds*:

(20) $$\sum_{i=1}^{\infty }p^{-\ell_{i}}\leq 1.$$

**Note** **13.** *From the proof of Theorem 17, it follows that the *branches of the state transition diagram constitute a prefix code since each word which corresponds to a branch of length k reaches some minimal affine subautomaton exactly at the k-th step, thus, the word cannot be a prefix of any other word which corresponds to another branch*. Note that words begin from the root of the tree, and the root is the initial state in the state-transition diagram. From the construction, it follows that the Kraft–MacMillan inequality for that code is equality. However, by using Corollary 1 rather than Lemma 2 in the proof, one constructs a prefix code such that $\sum_{i=1}^{\infty }p^{-\ell_{i}}=q\leq 1$ for any given 0<q≤1. In this case, the rest infinite paths of the complete p-adic tree that lead to no minimal finite affine subautomaton constitute a set of Haar measure 1−q. The automaton having such a state transition diagram will reach minimal subautomata which are finite and affine with probability 0<q≤1 rather than exactly 1. In that case, to automaton A, there corresponds a sharp wave function of the form (17) such that $\sum_{\left[S_{a,b}\right]\in \mathrm{Spec}\left(A\right)}q_{\left[S_{a,b}\right]}=q$ which therefore is normalisable. *For not to overload the exposition, in what follows we mostly deal with the case when q=1, i.e., with ultimately affine automata A, cf. Section 5.4.**

Let *X* be a random variable on the prefix code $G={\left(w_{i}\right)}_{i=1}^{\infty }$*X*; we denote via $q_{i}=\mathrm{Prob}\left(X=w_{j}\right)$ the probability that *X* is equal to the word $w_{i}$. By definition [61], the *entropy H(X) of the random variable X* is $H\left(X\right)=-\sum_{i=1}^{\infty }q_{i}\log_{p}q_{i}$, whereas the *mean* length of the codeword is $E\left(X\right)=\sum_{i=0}^{\infty }q_{i}\ell_{i}$.

There exists a prefix code such that $\ell_{i}=\left\lceil \log_{p}\left(1/q_{i}\right)\right\rceil$ for which the right-hand side inequality in (21) below holds (that right-hand side inequality is not true in general). The left-hand side inequality in (21) below holds whenever H(X)<+∞ and E(X)<+∞, becoming an equality if and only if $q_{i}=p^{-\ell_{i}}$ [61] [Theorem 4.3].

(21) H(X)≤E(X)<H(X)+1,

The time which a (both sharp and fuzzy) wave function takes to collapse can be expressed via the length of a word which reaches a state from some minimal affine subautomaton since the length of the word is the order of time expressed in the respective base; see the explanations in Section 5.3. This is why *in what follows, we deal with the lengths of the words rather than with time itself*. Note that when dealing with the lengths of the words, we may restrict considerations to the words over the alphabet {0,1,…,p−1} where *p* is a prime since fuzzy wave functions are constructed by using words over the alphabet {0,1}; see Section 5.4. The only difference between sharp and fuzzy wave function constructions for p=2 are the numerical values that are assigned to words by both the observers: The Little-endian assigns numbers to words by reading the words as the base-2 expansions of numbers whereas the Big-endian reads these words as (1+τ)-representations of numbers for 0<τ≪1. We stress that in what follows, “mean time of collapse” is synonymous with “mean word length” although the *actual* mean time of collapse *measured by the both observers* is different due to the inevitable nonzero measurement error. For instance, to the word of length *k* whose prefix is k−1 zeros and whose suffix is 1, the Little-endian assigns the value $2^{k}$, whereas the Big-endian assigns the value ${\left(1+\tau \right)}^{k}$, which for small τ and not too large *k*, is indistinguishable for this observer from 1 due to the measurement error. To put it in other words, the Little-endian’s measurements of time elapsed are much more accurate than are the Big-endian’s; the time within which the wave function collapses is large for the Little-endian, whereas that time is zero for the Big-endian up to the measurement accuracy of his equipment; although both the clocks the observers use are 2-adic, according to Theorem 18, the Big-endian can observe digits in the windows that are to the left of the (N−1)-th window at the face of the clock for *N* large, whereas Little-endian observes digits to the left of the lowest order position, i.e., from the rightmost window (cf., Figure 3). Nevertheless, we are going to show that *“time-energy” uncertainty in terms of the length of words in the state transition diagrams of automata holds for both observers*.

Let A be an ultimately affine automaton, cf. Section 5.4. Define the *automaton entropy* as

$$H_{A}=-\sum_{\left[S_{a,b}\right]\in \mathrm{Spec}\left(A\right)}q_{\left[S_{a,b}\right]}\log_{p}q_{\left[S_{a,b}\right]}.$$

For every $\left[S_{a,b}\right]\in \mathrm{Spec}\left(A\right)$, the probability $q_{\left[S_{a,b}\right]}$ is equal to the sum of all $p^{-\Lambda (w)}$, where Λ(w) is the length of a finite word *w* that reaches some state that belongs to some subautomaton from $\left[S_{a,b}\right]$ exactly at the Λ(w)-th step; the summation is over all these words. Let $C[S_{a,b}]$ be a code whose codewords are all these words *w*; then, $\sum_{\left(w\in \left[S_{a,b}\right]\right)}p^{-\Lambda (w)}=q_{\left[S_{a,b}\right]}$. Note that $\sum_{\left[S_{a,b}\right]\in \mathrm{Spec}\left(A\right)}\sum_{\left(w\in \left[S_{a,b}\right]\right)}p^{-\Lambda (w)}=\sum_{\left[S_{a,b}\right]\in \mathrm{Spec}\left(A\right)}q_{\left[S_{a,b}\right]}=1$, the codes $C[S_{a,b}]$ are disjointed for different $\left[S_{a,b}\right]\in \mathrm{Spec}\left(A\right)$, and the union of all these codes for all $\left[S_{a,b}\right]\in \mathrm{Spec}\left(A\right)$ is a prefix code C(A) such that $\sum_{w\in C(A)}p^{-\Lambda (w)}=1$. According to the above convention, the mean time T(A) of wave function collapse is the mean length of a codeword of the code C(A):

$$T\left(A\right)=\sum_{w\in C(A)}\Lambda \left(w\right)p^{-\Lambda (w)}$$

The inequality (21) implies that

$$T\left(A\right)\geq H_{T}=-\sum_{w\in C(A)}p^{-\Lambda (w)}\log_{p}\left(p^{-\Lambda (w)}\right)=\sum_{w\in C(A)}\Lambda \left(w\right)p^{-\Lambda (w)}=T\left(A\right),$$

where $H_{T}$ is the entropy of the code C(A). Therefore, *the mean time of collapse of the automaton wave function is equal to the entropy of the code C(A)*.

For every n∈N, let $T_{n}$ be a set of all codewords of length *n* from the code C(A). If $T_{n}\neq \emptyset$, then $T_{n}$ is a prefix code. All of these codes are disjointed, and their union is C(A). Therefore, $P_{N}=\sum_{n=1}^{N}\sum_{w\in T_{n}}p^{-n}$ is the probability that the wave function collapses for a time not greater than *N*. Let $H_{\mathrm{time}\leq N}\left(A\right)$ be the entropy of the prefix code $T\left(N\right)={⋃}_{n=1}^{N}T_{n}$; that is, $H_{\mathrm{time}\leq N}\left(A\right)=-\sum_{w\in T(N)}p^{-\Lambda (w)}\log_{p}\left(p^{-\Lambda (w)}\right)=\sum_{w\in T(N)}\Lambda \left(w\right)p^{-\Lambda (w)}$. As the probability assigned to w∈C(A) is $p^{-\Lambda (w)}$ and as $\sum_{w\in C(A)}p^{-\Lambda (w)}=1$, then for *N* not less than the length of the shortest word from C(A)), it holds that $H_{\mathrm{time}\leq N}\left(A\right)+H_{A}>0$. Moreover, as $H_{\mathrm{time}\leq +\infty }\left(A\right)=\sum_{w\in C(A)}\Lambda \left(w\right)p^{-\Lambda (w)}\geq \sum_{w\in C(A)}p^{-\Lambda (w)}=1$, then

$$H_{\mathrm{time}⩽+\infty }\left(A\right)+H_{A}⩾1.$$

If to minimal affine subautomata there are ascribed “energy levels” (e.g., if in the subautomata functions z↦az+b, the coefficients *a* are different and b=0) these inequalities may be judged as time-energy uncertainty relation since *if an observer measures the time which a wave function takes to collapse, he does not know for sure to which of the states the wave function has collapsed; on the other hand, if he knows to which of the states the wave function has collapsed, he does not know for sure how much time the collapse has taken*.

In a general case, these inequalities cannot be sharpened. Since ${⋃}_{n=1}^{\infty }T_{n}=C\left(A\right)$, then C(A) can be split arbitrarily into the disjointed union of sets $D_{1},D_{2},\ldots$, and as each of $D_{j}$ is itself a prefix code, there is an automaton D such that $C\left[S_{a,b}\right]=D_{j}$, (j=1,2,…). Indeed, the entropy $H_{\mathrm{time}\leq +\infty }\left(A\right)$ is determined by the code C(A) only, whereas $H_{A}$ is determined completely by the partition of the code C(A) into arbitrary nonempty subsets and by the “assigning of limit plots” to each of the subsets.

The codeword lengths in C(A) can be arbitrary as well.

**Theorem** **20** (On maximal prefix codes [62])**.** *For every non-decreasing map ℓ:N→N such that $\sum_{n=1}^{\infty }p^{-\ell (n)}=d\leq 1$ there exists a (maximal) prefix code $C=\{w_{n}:n\in N\}$ such that $\Lambda \left(w_{n}\right)=\ell \left(n\right)$, for all n∈N.*

That is, one can take any such code *C* for d=1, split all its codewords into a partition P(C) of nonempty subsets, assign to every subset S∈P(C) a limit plot of a finite automaton $S_{a,b}\left(S\right)$ arbitrarily, and construct a respective automaton A so that C(A)=C and all finite paths in every *S* lead to the $S_{a,b}\left(S\right)$.

We have that $H_{A}\in \left[0,+\infty \right]$, $H_{\mathrm{time}\leq +\infty }\left(A\right)\in \left[1,+\infty \right]$, T(A)∈[1,+∞] (as $T\left(A\right)=H_{T}=H_{\mathrm{time}\leq +\infty }\left(A\right)$), and nothing more definite can be said in the general case. It is possible that $H_{\mathrm{time}\leq +\infty }\left(A\right)=1$. For instance, let p=2, and let C(A)={1,01,001,0001,…}. Then, the entropy $H_{A}$ may be equal to 1 if different limit plots are assigned to different balls $B_{2^{-n-1}}\left(2^{n}\right)$. The entropy $H_{A}$ may be zero if the limit plots that are assigned to all these balls are equal one to another. One may split the set of all these balls into a partition of pairwise disjointed nonempty subsets and assign to each ball a limit plot so that to all balls from a subset, the same limit plot is assigned, but to balls that belong to different subsets, one assigns different limit plots. In all these cases, $H_{\mathrm{time}\leq +\infty }\left(A\right)=1$ (as the entropy is equal to T(A)), but the entropies $H_{A}$ are different.

Finally, consider *generating series $f_{A}\left(x\right)=\sum_{w\in C(A)}x^{\Lambda (w)}=\sum_{n=1}^{\infty }t_{n}x^{n}$, where $t_{n}$ is the number of all words of length n in the prefix code C(A)*. As $f_{A}\left(1/p\right)=1$, then for the radius $R_{A}$ of convergence of the series, it holds that $R_{A}\geq 1/p$, with $f_{A}\left(1/p\right)=1$. Hence, *the function $f_{A}\left(x\right)$ is differentiable at all points from $\left(-R_{A},R_{A}\right)$, but if $R_{A}=1/p$, then the derivative $f_{A}^{\prime }\left(x\right)$ may not exist at x=1/p or may go to +∞. However, $f_{A}^{\prime }\left(1/p\right)=p\cdot T\left(A\right)$, i.e., the derivative $f_{A}^{\prime }\left(1/p\right)$ determines the entropy $H_{\mathrm{time}\leq +\infty }\left(A\right)$*.

## 6. Discussion

In the paper, a number of mathematical statements are rigorously proven which, as a whole, advocate that answers to the questions as whether Nature at the smallest of scales is discrete or continuous, random and chaotic, or deterministic and predictable, solely depend on the free choice of metric, real or *p*-adic, with respect to which numerical experimental data are processed. The core idea is that rational *p*-adic integers, i.e., irreducible fractions whose denominators are coprime to *p*, are indistinguishable by measurement from real numbers due to the inevitable nonzero measurement error. The paper is motivated by the ideas of I. Volovich on *p*-adic mathematical physics, cf., [6], by G. ‘t Hooft’s cellular automaton interpretation of quantum mechanics, cf., [7], and (to some extent) by recent papers on superdeterminism by J. Hance, S. Hossenfelder, and T. Palmer, [3,5]. As a whole, the paper is information-theoretic by nature, so the results of the paper concerning causality, wave functions, entropic time-energy uncertainty relation, etc., which are rigorously deduced in the paper, may be considered as a contribution to J. Wheeler’s *it from bit* doctrine, cf., [14].

## Data Availability

Additional data can be found at https://www.researchgate.net/profile/Vladimir-Anashin/research.
